# Eating, Sleeping, Consoling for Neonatal Opioid Withdrawal (ESC-NOW): a Function-Based Assessment and Management Approach study protocol for a multi-center, stepped-wedge randomized controlled trial

**DOI:** 10.1186/s13063-022-06445-z

**Published:** 2022-08-09

**Authors:** Leslie W. Young, Songthip Ounpraseuth, Stephanie L. Merhar, Alan E. Simon, Abhik Das, Rachel G. Greenberg, Rosemary D. Higgins, Jeannette Lee, Brenda B. Poindexter, P. Brian Smith, Michele Walsh, Jessica Snowden, Lori A. Devlin, for the Eunice Kennedy Shriver National Institute of Child Health and Human Development Neonatal Research Network and the NIH Environmental influences on Child Health Outcomes (ECHO) Program Institutional Development Awards States Pediatric Clinical Trials Network

**Affiliations:** 1 Department of Pediatrics, Larner College of Medicine at the University of Vermont, 111 Colchester Ave Smith 5, Burlington, VT 05401 USA; 2 University of Arkansas for Medical Sciences, 4301 W. Markham St., Little Rock, AR 72205 USA; 3 Department of Pediatrics, University of Cincinnati, 3333 Burnet Ave ML7009, Cincinnati, OH 45229 USA; 4 Environmental Influences on Child Health Outcomes (ECHO) Program, Office of the Director, the National Institutes of Health, Three White Flint North 11601 Landsdown Street, Office 3D16, North Bethesda, MD 20852 USA; 5 RTI International, 3040 East Cornwallis Road, Research Triangle Park, NC 27709-2194 USA; 6 Duke University School of Medicine Duke Clinical Research Institute, 300 W. Morgan St, Durham, NC 27701 USA; 7 College of Health and Human Services George Mason University, 4400 University Drive 2G7 Peterson Family Health Science Hall Room 5415 Fairfax, Virginia, 22030 USA; 8 University of Arkansas for Medical Sciences, 4301 West Markham, #781 COPH Room 3234, Little Rock, Arkansas 72205-7199 USA; 9 Emory University School of Medicine, 2015 Uppergate Dr. NE, Suite 304, Atlanta, GA 30322 USA; 10 Pregnancy and Perinatology Branch of the Eunice Kennedy Shriver National Institute of Child Health and Human Development, 6710 Rockledge Dr. Wing B, Rm2321-D, Bethesda, MD 20892-7002 USA; 11 Arkansas Children’s Research Institute, University of Arkansas for Medical Sciences, 13 Children’s Way, ACRI Slot 512-35, Little Rock, AR 72202 USA; 12 Department of Pediatrics, University of Louisville School of Medicine, 571 South Floyd Street Suite 342, Louisville, KY 40202 USA

## Introduction

### Background and rationale

#### Public health impact

Increased opioid use has resulted in a dramatic increase in the number of infants born with in utero opioid exposure requiring management for NOWS [1–4]. Despite the significance of this problem, numerous critical gaps remain in our knowledge with respect to the best practices for identification and management of infants with NOWS, as well as our understanding of the outcomes of these infants [5, 6]. The opioid epidemic particularly impacts rural and underserved communities represented by the ISPCTN and participating Neonatal Research Network (NRN) sites, which makes our networks well poised to address these critical gaps and improve the care of infants with NOWS.

#### Background

##### Scope of the problem

The medical and non-medical use of opioids has increased substantially in women of childbearing age during the last decade [7]. In the United States (US), medical professionals wrote and dispensed 259 million opioid prescriptions in 2012 alone, an average of 82.5 opioid prescriptions for every 100 persons [8]. Approximately 28% of privately insured and 39% of Medicaid-enrolled women between 15 and 44 years of age filled an opioid prescription annually between 2008 and 2012 [9]. Every 3 minutes, a woman seeks care in an emergency department for prescription opioid misuse. In addition, illicit opioid abuse is also increasing dramatically [7]. Nearly 600,000 Americans reported a substance-use disorder involving heroin in 2015, with the strongest risk factor for heroin use being a history of prescription opioid misuse [3, 10]. The national rate of opioid use disorders in new mothers has quadrupled between 1999 and 2014, increasing from 1.5 to 6.5 per 1000 deliveries [11, 12].

The increased use and misuse of opioids during pregnancy has directly resulted in a 5-fold increase in the incidence of NOWS between 2004 and 2014 [13]. A retrospective analysis of a National Inpatient Sample showed that, among infants covered by Medicaid, the incidence of NOWS increased from 2.8 to 14.4 per 1000 births during this same period [13]. Additionally, analysis of an administrative database of 23 hospitals from 2013-2016 demonstrated a continued increase in the incidence of NOWS to 20 per 1000 live births [14]. Significant regional variation in the incidence of NOWS has been noted, with the highest rates seen in the Northeast and Southeast regions of the United States [1]. Researchers have found an increased incidence of infants with NOWS born to mothers who have high rates of long-term unemployment or who live in mental health shortage areas [15]. Rural areas are disproportionately affected by NOWS, with twice the rate of growth in the number of hospital deliveries complicated by maternal opioid abuse in rural communities compared with the rate of growth in urban communities between 2004-2013 [16]. The proportion of infants with NOWS born into rural communities increased from 12.9% in 2003 to 21.2% in 2013 [16]. Therefore, improving care for infants with NOWS will particularly impact the rural areas served by many ISPCTN and NRN sites. Additionally, compared with their urban peers, rural infants affected by perinatal opioid misuse are more likely to come from lower-income families who have public insurance [16]. Nationally, state Medicaid programs enroll 60% of mothers with perinatal substance use and more than 80% of infants with NOWS [1, 2].

##### Recognition and assessment of neonatal opioid withdrawal syndrome

Some infants with in utero opioid exposure may have mild signs of NOWS that do not significantly impact the infant’s ability to feed, sleep, and function, while others may have more severe signs that require pharmacologic therapy to avoid negative effects on growth and development [17]. Physicians use observer-rated scales in clinical practice to quantify the severity of withdrawal and to guide pharmacotherapy [4]. Yet, current scales have not undergone rigorous instrument development and validation [18, 19]. Ninety-five percent of institutions in the United States use the FNAST, with its various modifications [20]. Preliminary data from the ACT NOW Current Experience Study, a chart review conducted at 25 sites within the ISPCTN and 5 sites within the NRN, found that all 30 participating sites used the FNAST or a modification of the FNAST for the assessment of infants with NOWS as part of usual institutional care. Loretta Finnegan developed the FNAST in 1975, and medical personnel currently use this and several modified versions. The tool was initially found to have an inter-rater reliability (IRR) of 0.82 (0.75-0.96), but it has not been subsequently validated for the evaluation of infants with NOWS, although researchers have studied normative values in newborns unexposed to maternal substances [21]. Researchers and clinicians remain concerned about the length of the tool, [22, 23] its inherent subjectivity, [24] and the need to disturb infants for formal assessments [25]. In addition, investigators have concerns that the FNAST and modifications of the FNAST may overestimate the need for pharmacologic therapy, as the formal score incorporates all signs of withdrawal, including those that may not be clinically significant. This overestimation has been linked to increased length of hospital stay and hospital costs [26].

The ESC Care Tool is an alternative assessment and management tool developed and subsequently implemented at several sites as part of quality improvement (QI) initiatives based on the original ESC approach developed by Grossman and colleagues at Yale [25]. The ESC Care Tool uses a non-invasive, simplified, function-based assessment that evaluates the infant based on his/her ability to eat, sleep, and be consoled. The tool's design provides continued emphases on the role of the family/caregiver in the assessment and care provided for their infants and on non-pharmacologic care as the first-line treatment for infants with NOWS. If an infant is able to feed effectively within 10 minutes of showing hunger (breast-feed well x 10 minutes or take 10 mL [or age-appropriate volume] by alternative feeding method), to sleep undisturbed for 1 hour or longer, and is able to be consoled within 10 minutes, pharmacologic treatment is not initiated or escalated. If the care team assesses that the infant is having difficulties in one of these areas related to NOWS, the care team first attempts to optimize non-pharmacologic interventions. If these attempts are unsuccessful, the care team will initiate or escalate pharmacologic therapy.

##### Initial eating, sleeping, consoling approach

The ESC approach, an approach that emphasizes parental involvement, simplifies the assessment of infants with NOWS, and focuses interventions on non-pharmacologic therapies, began its evolution at Yale-New Haven Children’s Hospital over a 5-year period of QI work. During this time, the proportion of infants prenatally exposed to methadone who received pharmacologic treatment for NOWS decreased significantly from 98% (54 out of 55 infants) in the baseline period (January 2008-February 2010) to 14% (6 out of 44 infants) in the post-intervention period (May 2015-June 2016), *P* < 0.001. The average length of stay (LOS) for these infants also decreased significantly from 22 to 6 days (*P* < 0.001) [25]. There were no reported seizures during the initial birth hospitalization or need for readmission within 30 days of discharge related to signs of withdrawal for the post-intervention group. Although the results of this QI work appear quite impressive, it is unclear how generalizable this work is, as the pre-intervention rate of pharmacologic treatment was much higher than national estimates at 98% of methadone-exposed infants [4]. Additionally many infants with NOWS are exposed to opioids other than methadone (e.g., buprenorphine and illicit opioids).

On direct comparison, Yale-New Haven’s ESC approach, studied as a QI measure, appears to trigger the initiation of opioid replacement therapy for significantly fewer infants than use of the FNAST approach. The Yale group, following their transition to ESC-based assessments, completed a retrospective comparison of treatment decisions for 50 consecutive opioid exposed infants (March 2014-Aug 2015) [26]. These infants had FNAST scores recorded every 2 to 6 hours, but clinical personnel managed these infants based on their ESC assessments alone. Management decisions based on the ESC assessment resulted in morphine initiation for 6 infants (12%), compared with 31 infants (62%) who medical professionals would have treated using the FNAST (P < 0.001). Additionally, using the ESC-based assessments, medical personnel initiated or increased morphine on 8 patient days (3%), compared with 76 patient days (26%) predicted using the FNAST (P < 0.001) [26]. Again, the study reported no readmissions or adverse events (AEs).

##### Eating, sleeping, consoling care tool development

Other groups have subsequently worked to standardize implementation of the assessment and management components of the ESC care approach, through the development and testing of a formal ESC Care Tool. Initial evaluation of the assessment component of the ESC Care Tool, using standardized training and simulated case scenarios, has demonstrated high inter- and intra-rater reliability [27]. Training in the use of the ESC Care Tool and the overall care approach, with standardized training materials, continues to be evaluated and improved, allowing for feasible implementation in even small community hospitals. Faculty at Children’s Hospital at Dartmouth-Hitchcock Medical Center, Boston Medical Center, and Yale-New Haven Children’s Hospital collaborated to develop training materials, including Instructional Manual, ESC Care Tool with definitions, Newborn Care Diary, ESC training video, and written and videotaped case scenarios with answer key. Sites within The Northern New England Perinatal Quality Improvement Network are currently using these materials to facilitate training as part of a network-wide QI initiative.

Physicians at one of the institutions involved in these development efforts, recently published on their QI results following implementation of the ESC care approach. This institution utilized a pilot version of the ESC Care Tool and showed more modest but consistent findings to those at Yale. The researchers found a decrease in pharmacologic treatment from 87% to 40% and a reduction in LOS from 17 to 11 days, with no AEs noted [28].

##### Further study

Although outcomes following implementation of the ESC care approach, inclusive of the ESC Care Tool, appear promising and initial accounts suggest that it is safe, we need to rigorously study this care approach to show safety, efficacy, and generalizability of its use in the care of infants with NOWS. Reports on the ESC care approach to date have been from hospitals where the majority of the mothers are compliant with medication-assisted treatment and are highly motivated to care for their infants. Furthermore, the potential effects of the care provided, using the ESC care approach, on infant and family well-being after discharge are unknown and important to assess [5, 29]. In the proposed trial, comparison of the short- and long-term outcomes for infants managed with the ESC care approach versus those managed with usual care will move us closer to an evidence-based approach for the evaluation and management of infants with NOWS, thus meeting a top research priority in the field [5, 6].

#### Hypotheses

##### Primary hypothesis

Among infants evaluated for NOWS, the ESC care approach will reduce the length of time until infants are medically ready for discharge by an average of 4 days, compared to usual institutional care with the FNAST or modification thereof.

##### Secondary hypothesis

Among infants evaluated for NOWS, use of the ESC care approach will result in an improvement in infant neurobehavioral functioning and family well-being, when compared to usual institutional care with the FNAST or modification thereof.

##### Justification of hypotheses

We hypothesize that use of the ESC care approach for the evaluation and management of infants with NOWS will safely reduce the average length of time until infants are medically ready for discharge, compared with usual care with the FNAST or modification thereof. We selected the primary outcome, average length of time until infants are medically ready for discharge, due to the potential for infants to remain in the hospital beyond this point because of social factors and the previously described potential impact of and link between a reduction in hospital stay and the following:

- Improved maternal and infant attachment/bonding [30, 31]
- Decreased hospital complications
- Increased benefit to society in reduced healthcare costs [2, 13, 32]

Additionally, we hypothesize that use of the ESC care approach will have minimal to no impact on infant safety, while resulting in the following outcomes:

- Reduction in the need for initiation of opioid replacement therapy (i.e., morphine, methadone, or buprenorphine)
- Decrease in the total postnatal opioid exposure
- Improvement in the timeliness to initiation of opioid replacement therapy, when required
- Decrease in the need for adjuvant therapy
- Increase in the proportion of infants who directly breastfeed
- Increase in the proportion of infants receiving their mothers’ own breastmilk

We also hypothesize that use of the ESC care approach will improve postnatal attachment and bonding, and will enhance infant well-being and neurobehavioral functioning and development compared to usual care. Further, we hypothesize that use of the ESC care approach will enhance maternal well-being and the family environment after discharge. An important component of the ESC care approach is the reported fostering of a collaborative relationship between the primary caregiver(s) and the inpatient clinical team through the co-assessment of the infant’s severity of withdrawal and shared treatment plan development. Interviews conducted with families as part of the QI implementation of the ESC Care Tool consistently suggest that this element may reduce the social and emotional impact of the infant’s hospitalization on the family. However, while many families expressed feeling like they were an integral part of their infants’ care team and reported decreased anxiety and reduction in stigma during the initial birth hospitalization, [33] these families were poised to actively participate in the care of their infants, and such results may not be consistent across all families/caregivers. Thus, we must consider that families/caregivers who are not as well poised to actively participate in the care of their infants may experience more stress if their infants are discharged home earlier.

Our assessment of key markers of infant and family well-being in the subpopulation of infants whose caregiver(s) provide informed consent will allow for further insight into safety. This will also provide an opportunity to examine not only often-assessed intermediate outcome variables (time until medically ready for discharge and need for opioid replacement therapy), but also to examine longer-term outcomes, such as infant neurobehavioral functioning and development, maternal-infant attachment and bonding, and family well-being and functioning.

#### Study design type

In this stepped-wedge cluster randomized controlled trial with transition period, the protocol study team will compare the ESC care approach to usual institutional care with the FNAST or modification thereof. Randomization will occur at the site level. The protocol study team will randomize approximately 24 US sites into 8 blocks. Each block will transition from usual care to the ESC care approach for the evaluation and management of all infants with NOWS at various time intervals (see Table 1). Sites will use the care approach randomly assigned to their block during each study period for the evaluation and management of all infants with NOWS cared for at the site. During the initial birth hospitalization, the site research team will collect data under waiver of consent for infants who meet eligibility criteria. The number of infants enrolled per period at each site will vary throughout the study, due to fluctuations in the number of infants managed for NOWS at each site during each period. However, the goal is for each site to enroll at least 4 infants per period. The site research team will obtain informed consent from the legal guardian(s) to obtain long-term outcomes for eligible infants and caregivers. Site research team members may obtain this consent at any point during the hospital stay for infants who meet the trial’s inclusion criteria.

**Table 1:**
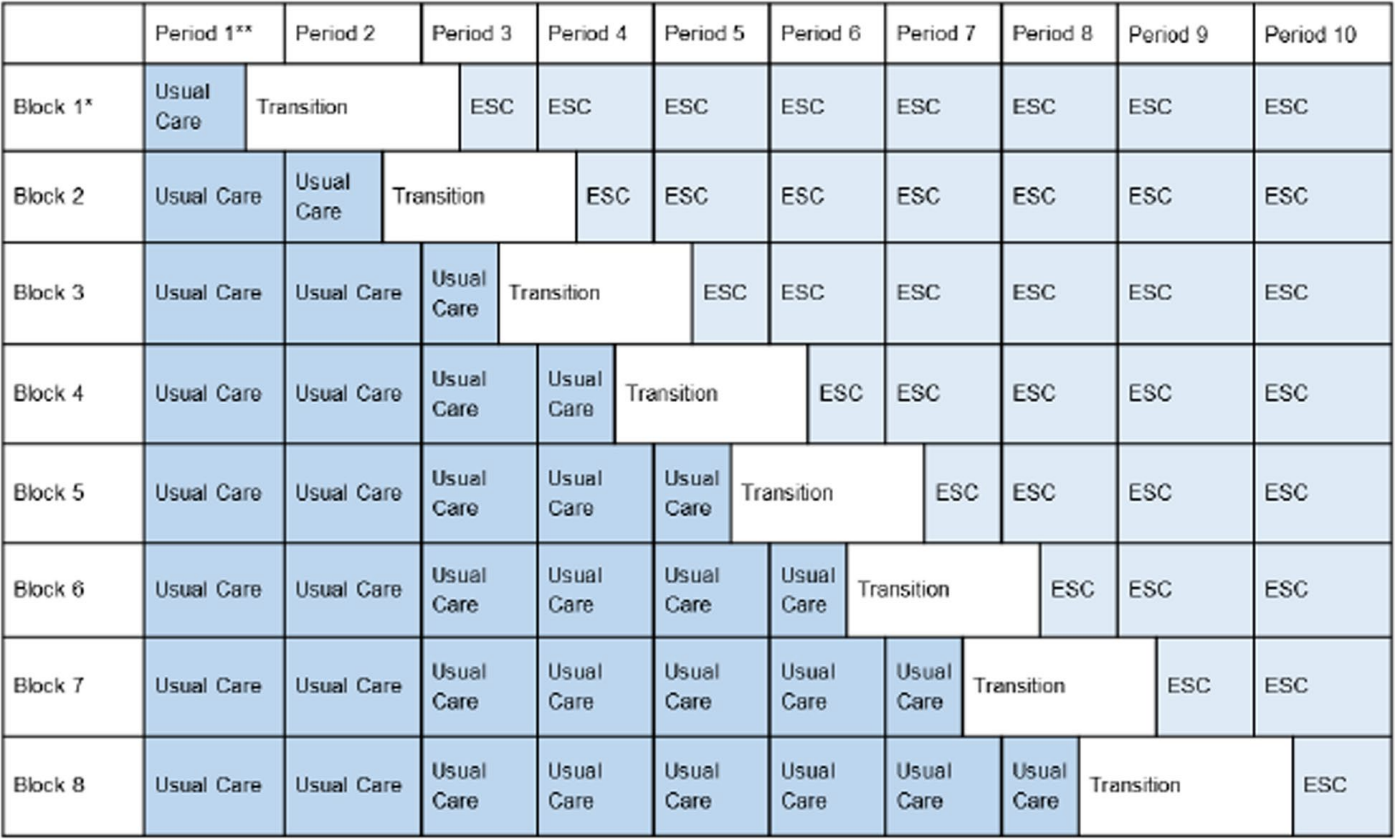
Stepped-wedge cluster randomized controlled trial with transition period

*Each block will consist of 3-4 sites

**Each period will be 2 months in duration, except for the transition period, which will be 3 months, and the intervention periods bordering the transition, which will be 1.5 months/6 weeks in duration

##### Justification of study design

The protocol study team selected a stepped-wedge cluster design due to three main considerations:

1. Transition to the ESC care approach requires a significant cultural shift in the management of infants with NOWS. This type of cultural change is most effective when applied at the level of the population covered by the hospital and not on a subset of random infants with NOWS within the hospital, thus making a cluster study design important.
2. Interim analysis of the ACT NOW Current Experience study allowed for estimation of an intracluster correlation coefficient (ICC) based on the LOS outcome measure. Using LOS as a proxy for our primary outcome, time until infant is medically ready for discharge, the number of sites required to adequately power the trial based on a parallel cluster design with estimated ICC=0.25 would be prohibitive. The stepped wedge design makes the study feasible by allowing each site to serve as its own control in a pre/post analysis and thus, the variation between sites is of less statistical significance.
3. Additionally, the results of QI projects have inspired many healthcare providers to consider transition to the ESC care approach. A brief questionnaire, sent to investigators at the available study sites, demonstrated an increased willingness to participate in the trial if we integrated a transition to the ESC care approach into the study design.

For these reasons, the protocol study team has designed a stepped-wedge cluster randomized trial with the intervention applied at a cluster level and applied to all participating sites by the end of the 20-month study period, with the timing of transition to ESC randomized. This study design also allows for differentiation between the effect of the intervention and unanticipated time-related confounders.

## Methods

### Study population

#### Inclusion criteria

##### Site level

- The site is willing, able, and has nurse management and administrative commitment to transition to the ESC care approach at the randomly allocated time
- The site currently uses the FNAST or modification thereof for the assessment of withdrawal severity for infants with NOWS
- The site currently provides opioid replacement therapy (i.e., morphine, methadone, or buprenorphine) for the pharmacologic management of infants with NOWS

##### Infant level

- The infant is being managed for NOWS at an eligible site (i.e., receiving non-pharmacologic care, assessments for withdrawal severity, +/- pharmacologic care)
- The infant is ≥ 36 weeks gestation
- The infant satisfies at least 1 of the following criteria:
- Maternal history of prenatal opioid use
- Maternal toxicology screen positive for opioids during the second and/or third trimester of pregnancy
- Infant toxicology screen positive for opioids during the initial hospital stay

#### Exclusion criteria

##### Site level

- The site currently manages < 20 opioid-exposed infants annually
- The site routinely discharges/transfers infants from the hospital on opioid replacement therapy (i.e., morphine, methadone, or buprenorphine). We define routine discharge/transfer as ≥10% of infants who receive opioid replacement therapy for NOWS at the site

##### Infant level

- Infant has major birth defect(s)
- Infant has neonatal encephalopathy (inclusive of hypoxic ischemic encephalopathy), a metabolic disorder, stroke, intracranial hemorrhage, or meningitis diagnosed by 60 hours of life
- Infant was receiving respiratory support (any positive pressure or oxygen therapy) unrelated to pharmacologic treatment for NOWS at 60 hours of life
- Infant was receiving antimicrobial(s) at 60 hours of life
- Infant has received any major surgical intervention in the first 60 hours of life
- Postnatal opioid exposure other than for treatment of NOWS in the first 60 hours of life
- Outborn infants transferred at >60 hours of life or treated with opioids for NOWS at the transferring hospital
- Infant’s biological mother or primary caregiver is positive or under investigation for COVID-19 at 60 hours of life

##### Additional infant-level exclusion criteria for consented portion of the study

Infant’s primary caregiver does not speak, read or write English.

### Detailed study procedures

#### Study events

Table 2 outlines the study events from the initial hospital stay through 24 months.

**Table 2:** Study event schedule

Evaluation/Procedures	Hospital Stay	Hospital Discharge	1 month post discharge	3 months of age^a^	6 months of age^a^	9 months of age^a^	12 months of age^a^	18 months of age^a^	24 months of age^a^
Maternal and infant medical history	X								
Neonatal opioid withdrawal scoring/ assessments	X								
Date/time of initiation and number of doses of opioid replacement therapy administered (Infant)	X								
Date/time of initiation dose and number of doses of adjuvant therapy administered (Infant)	X								
Date/time when medically ready for discharge (Infant)	X								
Date/time discharged (Infant)		X							
Weight, length and head circumference (Infant)	X	X							O
Feeding type (Infant)	X	X							
Seizures, accidental trauma (i.e., dropped infants) and respiratory insufficiency (Infant)	X	X							
Serious Adverse Events (Infant)	X	X							
Acute/urgent care and/or ER visits and hospital readmissions				X					
Non-accidental trauma & death (Infant)		X		X					X
Infant Behavioral Questionnaire (IBQ) – very short form (Infant)				O			O		
Caregiver questionnaire (CQ) (enteral feeds, acute/urgent care and/or ER visits and readmissions) (Infant)			O	O	O		O		O
Brief Infant Sleep Questionnaire (BISQ) (Infant)				O			O		
Patient Reported Outcome Measurement Information System (PROMIS) Short Forms (Caregiver(s))		O			O				O
Maternal Postnatal Attachment Questionnaire (MPAQ) (Caregiver(s))		O			O				
Parenting Sense of Competence Scale (PSOC) (Caregiver(s))		O			O				
Adverse Childhood Experiences (ACE) questionnaire									O
Family Environmental Scale (FES) – Relationship Dimension Form R (Caregiver(s))				O					
Bayley Scales of Infant and Toddler Development, Fourth Edition (Bayley-4): Cognitive, Language and Motor									O
Brief Infant-Toddler Social and Emotional Assessment (BITSEA)									O
Contact information update	O	O	O	O	O	O	O	O	

(X) Evaluations/procedures assessed under waiver of consent

(O) Evaluations/procedures performed with informed consent

^a^Procedures occurring at ≤12 months of age may occur within ±3 weeks of stated time point. Procedures occurring at 18 and 24 months of age may occur within ±6 weeks of stated time point

#### Screening

**Fig. 1:**
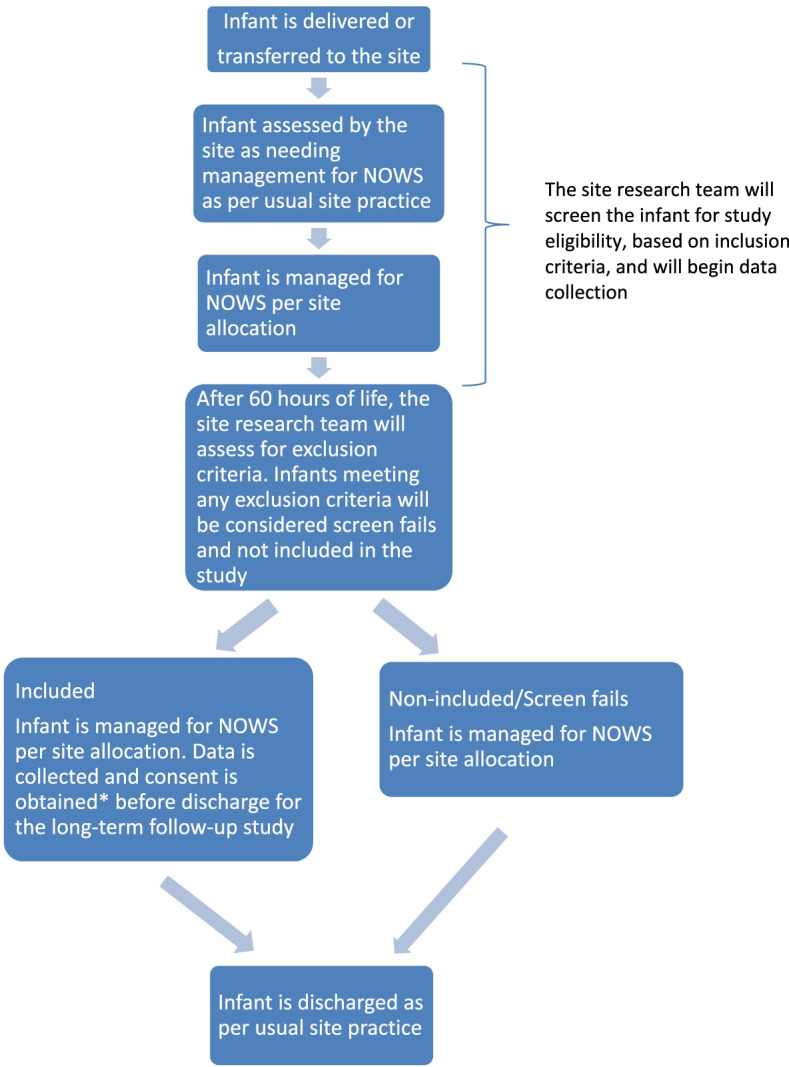
Screening and enrollment procedures. *The site research team may obtain consent for the long-term follow-up portion of the study at any point during the hospital stay for infants who meet the trial’s inclusion criteria. To optimize recruitment it will permissible to obtain initial consent up to one month after discharge

The protocol study team will screen interested sites for eligibility, and will randomize eligible sites into one of 8 blocks, as illustrated in Table 1. With this study design, all infants with NOWS cared for at a site, will be evaluated and managed using the care approach that the site is assigned to during the study period. Therefore, individual infant screening will not be required before initiation of this study protocol. The process will be as follows (see Fig. 1), after birth the inpatient clinical team will assess infants as at risk for NOWS and initiate management for NOWS based on the site’s usual methods of identification. The initiation of clinical management for infants with NOWS will not be impacted by the study intervention. The site research team will identify potential participants for the trial based on their eligibility following review of the medical record after delivery. The site research team may obtain informed consent for infant and caregiver participation in the long-term outcomes portion of the study at any point during the hospital stay for infants who meet the trial’s inclusion criteria. The site research team will evaluate for exclusion criteria after the infant’s first 60 hours of life. The site research team will note infants as screen fails who meet any of the exclusion criteria, and will not collect additional data for these infants. Sites may enroll up to 16 infants per period; screen fails are not included in this total. Only infants enrolled in the study may be approached for consent and included in the long-term follow-up study.

#### Consent procedures

##### Waiver of consent

Since this study is a stepped-wedge cluster randomized controlled trial, the intervention will take place on a site-wide basis and sites will transition their practice for all infants with NOWS cared for at the site during the study period. Thus, we will request a waiver of consent from the central institutional review board (IRB) at the University of Arkansas for Medical Sciences for the primary outcome and previously outlined short-term secondary outcomes. There is debate in the clinical trial and ethics literature about the issue of individual consent for cluster-randomized trials. However, in general, a study may proceed without individual consent if conditions for a waiver of consent are satisfied and participants (or legally authorized representatives) are provided with a description of the intervention to which their cluster has been randomized.35

As stated in Code of Federal Regulations [45 CFR 46.116 (d)], an IRB may approve a consent procedure that does not include, or that alters, some or all of the elements of informed consent set forth in this section, or waive the requirements to obtain informed consent, provided the IRB finds and documents that all of the following conditions are met:

1. The research involves no more than minimal risk to the participants;
2. The waiver or alteration will not adversely affect the rights and welfare of the participants;
3. The research could not practicably be carried out without the waiver or alteration; and
4. Whenever appropriate, the study team will provide participants with additional pertinent information after participation.

The justification for a waiver of informed consent from caregiver(s) for the short-term outcomes meets the above criteria per the following:

1. *The research involves no more than minimal risk to the participants.*
  Both usual care using the FNAST and the ESC care approaches are currently used at sites across the country, and the optimal care approach for the management of infants with NOWS is unknown. Additionally, there are no study procedures or study interventions within this protocol that would qualify as more than minimal risk, based on federal regulations, for either intervention group.
2. *The waiver or alteration will not adversely affect the rights and welfare of the participants.*
  As the best management for infants with NOWS is unknown, there is no universally accepted standard of care, and both care approaches are currently being used at sites across the country. Therefore, participants receiving care via either model should not have their rights and welfare adversely affected.
3. *The site research team could not practicably carry out this trial without the waiver or alteration.*
  Carrying out this trial and obtaining generalizable results would not be feasible if obtaining informed consent were required. Obtaining informed consent from legally authorized representatives of infants in this population is difficult due to multiple factors. The interventions conducted in this study begin shortly after birth and recruitment during this vulnerable period can be extremely difficult. Researchers have experienced this difficulty in a number of trials that have failed to successfully recruit this population shortly after birth [34–36]. Seeking consent shortly after delivery may not only result in recruitment failure, but may result in achieving consent only among a less generalizable group of “responders”, which may introduce bias and diminish generalizability. This could be particularly problematic in this trial where the success of the intervention may be particularly susceptible to caregiver effort and engagement.
  Seeking consent later in the hospital stay for the long-term follow-up portion of the study will allow for relationship and trust building between the consenting member of the site research team and the primary caregiver(s). This will likely allow for improved consent rates and improved generalizability. If consenting members of the site research team sought early consent and only a group of “responders” were consented, it is unclear whether this would have a differential impact across the study interventions. Additionally, the intervention is instituted at the site level and will represent a culture change. The site will use the assigned approach to care for all infants with NOWS during the trial period. Therefore, if consent were required, obtaining consent would not alter the care approach used for the infant. Thus, the benefits afforded by using a waiver of consent outweigh the risks to the infant receiving the same management. If the clinical team used two different care models at the same time and at the same site, patient safety could be at risk and care potentially compromised due to the use of inconsistent care practices at the site.
4. *Whenever appropriate the study team will provide participants with additional pertinent information after participation.*
  Throughout the study, the site research team will provide participants with additional pertinent information when appropriate. The protocol study team will develop a handout that the site research team will give to the caregiver(s) of all infants with NOWS cared for at the site throughout the study period. This will fulfill the suggested framework [37] of participants being “provided with a detailed description of the interventions to which their cluster has been randomized.”

##### Consent for assessment of long-term outcomes

Members of the site research team will work with families/caregivers to obtain informed consent for: 1) parent/caregiver questionnaires that will assess caregiver well-being (e.g., parenting stress, attachment and bonding, depression, anxiety, etc.) and infant well-being (e.g., diet, sleep, neurobehavioral functioning, etc.), and 2) in-person follow-up visit at 24 months to assess neurodevelopmental outcomes and growth measures. Consent will contain basic information on recognition and support (consistent with regulatory requirements at each site) for mental health issues including suicidality among caregivers. The consent will also contain basic information on notification of child protective services (consistent with state law) should researchers or members of the clinical team have suspicion of child neglect or abuse. The site research team will obtain written, informed consent from primary caregiver(s) (e.g., biological parents, adoptive parents, or state-appointed guardians) prior to administration of the first questionnaire.

As previously outlined, participants may be consented up to 1 month after discharge. To facilitate this process, and due to the ongoing COVID-19 pandemic, remote consenting will be allowed. All communications will be done via HIPAA-compliant methods such as telephone, personal delivery of documents, US postal service, REDCap or other compliant electronic platform. The remote consent process will parallel the consent processed used for in-person consenting. The only difference will be the method(s) of communication. The study team will ensure that, as with in-person consenting, the participant is given sufficient opportunity to ask questions, is able to understand the nature of this study and what participation entails, and is provided a copy of the final, completed consent signed by all parties involved, including the research team member who obtained consent and, when applicable, the site investigator. This final, signed consent will be provided via a HIPAA-compliant method or a method that the participant has agreed to in writing. The study team members working on the consenting process will ensure that any participant who is consenting remotely has the authority to consent.

##### Detailing barriers to consent and participation

The site research team will ask non-consenting parents/caregivers to answer questions specific to perceived or actual barriers to participation and their choice not to be involved in the long-term outcome portion of the study. The site research team will inform non-consenting parents/caregivers about the purpose of these questions and that they are not required to answer them. The site research team will record responses without linking identifiers. The protocol study team will not permit an amendment of the consent form for previously non-consenting parents/caregivers that wish to consent following these questions. The protocol study team will use the data collected to improve site-specific and study-wide recruitment strategies for this trial and to inform future trials in this field.

#### Randomization procedures

This is a stepped-wedge cluster randomized design with a transition period wherein we will randomize participating study sites, rather than individual infants. All sites will implement the ESC care approach at some point during the trial; the random elements are two-fold: 1) randomization into the blocks, and 2) randomization of blocks to the time point at which each block implements the ESC care approach, the so-called “step” of the stepped-wedge design.

**Fig. 2:**
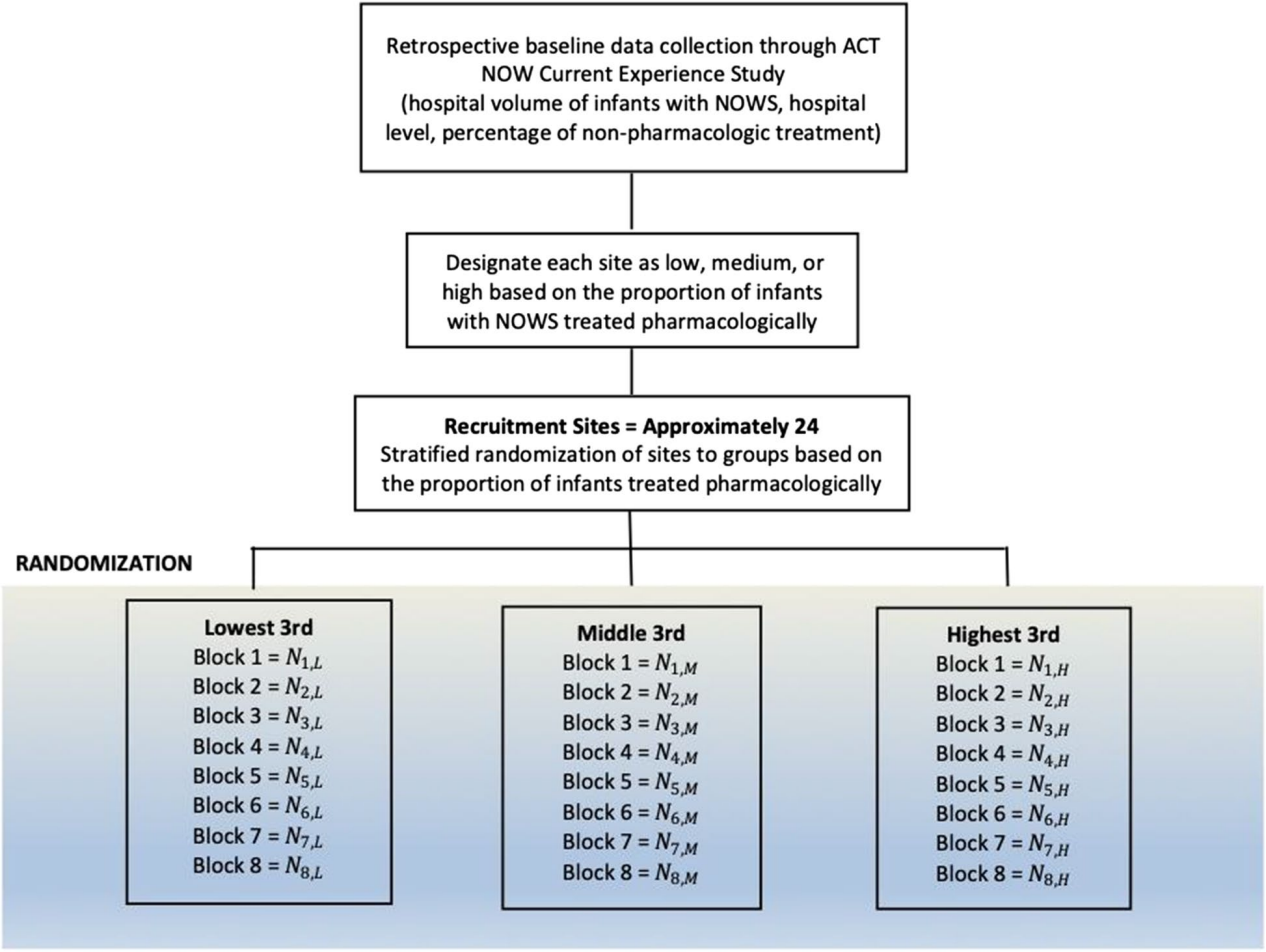
Randomization Flow Diagram

A statistician at the independent Data Coordinating Center (DCC) for the trial will generate a randomization list using SAS 9.4 (SAS Institute Inc., Cary, NC). The protocol study team will use the proportion of infants with NOWS treated pharmacologically at each site as the variable to stratify randomization (i.e., lowest 3rd, middle 3rd, highest 3rd). The protocol study team will identify this proportion using the results of the ACT NOW Current Experience Protocol, a retrospective data collection that details the inpatient identification, assessment, and management of infants with NOWS at the ISPCTN and participating NRN sites. The protocol study team will conduct a brief survey to obtain similar estimates from other interested sites. The protocol study team will randomize sites in each stratum into one of 8 blocks (Fig. 2). Once the protocol study team randomizes each site into blocks, computer-generated random numbers from a uniform distribution will determine the order in which the block of sites step into the transition and implementation period for the ESC care approach. The DCC will hold the randomization list. Due to the nature of a stepped-wedge cluster randomized controlled trial, the protocol study team can only enforce limited blinding. The protocol study team will notify sites of their allocated block following randomization.

Integration of the ESC care approach is a complex process and training of the hospital staff will be time-intensive. Thus, we expect to observe no quantifiable effects on the outcomes of interest during the transition period. Hence, this study has a planned transition period. The site research team will collect data on primary and secondary outcomes for all study periods, excluding the transition period. As this trial has sufficient power, we have planned for the transition period to be 3 months in duration to allow for adequate time for training and implementation at the sites. To maintain this, the preceding usual care period and the initial ESC period will each be 1.5 months/6 weeks in duration.

### Study intervention and comparison

All sites will provide usual institutional care, including the use of the FNAST, or modification thereof, for the evaluation and management of infants with NOWS during Period 1 (see Table 1). After the first period, the sites in Block 1 (3-4 sites) will move into the transition period. During the transition period, sites will participate in education and training modules conducted through a centralized training platform. The protocol study team will standardize education and training across sites during each block’s designated transition period. Educational modules will include an introduction and overview of the ESC care approach, education on trauma informed care and bias, and a general review on caring for infants with NOWS and the importance of non-pharmacologic care. Training will occur in a train-the-trainer format, and will include off-site or teleconference ESC training for a core group of site champions with subsequent on-site training of clinical personnel. Following the transition period, sites within Block 1 will move into the first ESC period and each of the other blocks will move into their next designated intervention period. The site research team will collect data during all intervention periods of the study and make comparisons between these interventions (usual care versus ESC care approach).

#### Usual institutional care

##### Intervention

- Following delivery or transfer, the care team will initiate care for NOWS per usual practice. The clinical team will use institutional practices and protocols to guide non-pharmacologic care, to assess the infant using the FNAST or modification thereof, and to guide pharmacologic care.
- If needed, the clinical team will initiate pharmacologic treatment per the site’s usual practice and/or treatment protocol, and escalation, weaning, and discontinuation of pharmacologic care will be per the site’s usual care. Opioid replacement therapy given (morphine, methadone, or buprenorphine) will be per site preference, as will adjuvant therapy used (clonidine or phenobarbital).
- The clinical team will monitor each infant requiring opioid replacement therapy for signs of escalating withdrawal symptoms following discontinuation of this treatment and will consider discharge per the site’s usual practice.
- The clinical team will use FNAST or modification thereof to assess infants after birth and consider discharge for infants who do not require pharmacologic treatment per the site’s usual practice.
- The DCC will develop a monitoring plan for each site’s compliance with usual care during this period.
- Infants with antenatal opioid exposure born or transferred to the site during the usual institutional care intervention will be managed per this care approach throughout their admission (this includes infants who remain admitted when the site enters the transition period), and the site research team will collect their data and use it for the study analysis.

#### Transition period

##### Intervention

**Education**

- All research and clinical nurses, advanced practice providers, and physicians who care for infants with NOWS at each participating site will complete educational modules. These modules will include an introduction and overview of the ESC care approach, education on trauma informed care and bias, and a general review specific to caring for infants with NOWS. The latter will include an emphasis on the importance of non-pharmacologic care, as well as on the importance of differentiating the etiology of symptoms common to NOWS.
- The protocol study team will assess completion of these modules through pre/post assessments. Post assessments will require 80% correct responses for completion. Participants will be able to retake each of the lessons until he/she achieves a correct response rate of 80%.

**Training and implementation**

- The protocol study team will conduct all training in a train-the-trainer format supported by nationally known clinical experts.
- The protocol study team will train a core group of site champions, which may include research and clinical nurses and physicians, in the use of the ESC care approach during the designated transition period. Education and training on the optimal use of the ESC care approach will include an introduction and overview of the ESC care approach, review of the Instructional manual, review of the ESC Care Tool with definitions and Newborn Care Diary, ESC training video, and review of written and videotaped case scenarios. The site champions will access the components of this training through the educational platform, and the protocol study team will track completion through pre/post assessments. The protocol study team will provide an electronic copy of the ESC Care Tool Instructional Manual for each site in anticipation of entry into the transition period.
- After the training, the core group of site champions will view and score cases until each member of the group consistently attains 100% reliability on standardized patient assessment cases (6/6 items). We define this as three consecutive assessments with 100% reliability as compared to national experts in the field. Once consistently achieving 100% reliability, the protocol study team will consider these individuals the "gold-star raters."
- This core group will train all other clinical personnel who care for infants with NOWS at their site, including, but not limited to, nurses, advanced practice providers, and physicians in all areas where these infants receive care. These areas may include, but are not limited to, the well-baby nursery, pediatric unit, and neonatal intensive care unit. After the training, clinical personnel will view and co-assess cases with the “gold-star raters” using the ESC IRR tool until clinical personnel consistently achieve 80% agreement (5/6 items). Once a member of the clinical team reaches 80% IRR, the site research team will clear the trainee for independent assessment. If the trainee consistently achieves 100% reliability, the site research team will consider him/her to be a “gold-star rater”, and may ask the trainee to function in this capacity. Site staffing levels will determine the number of “gold-star raters" at a site, with the goal of having one “gold-star rater” available on each shift. The protocol study team will require clinical personnel who are unable to attain 80% reliability to complete supplementary training. Clinical personnel hired after the initial training will complete the educational modules and ESC training at the site inclusive of co-assessing cases with “gold-star raters” using the ESC IRR tool to demonstrate 80% agreement.
- To ensure fidelity of the assessments, the protocol study team will assess the reliability of the “gold-star raters” at each site during the implementation phase of the transition period. The protocol study team anticipates each “gold-star rater” will maintain 100% reliability in scoring on patient assessment cases. The “gold-star raters” will then gauge reliability for the clinical team by assessing 10 individuals during the implementation phase by using the ESC IRR tool and written or video case scenarios on the training platform. For clinical personnel who fail to maintain the target of 80% reliability in scoring during the implementation period, the protocol study team will utilize just-in-time training through a centralized training platform until he/she achieves 80% reliability in assessments. When staffing allows, members of the care team who have reliability less than 80% should not be assigned to care for infants with NOWS until improved reliability is demonstrated through the just-in-time training process.
- Infants with antenatal opioid exposure born or transferred to the site during the transition period but before the site has implemented ESC, will be managed with usual institutional care. Once a site implements ESC, the site will manage all infants born or transferred to the site with the ESC care approach. For those infants receiving ongoing care for NOWS at the time of ESC implementation, the protocol study team will leave the care approach used for the continued care of these infants to the discretion of the clinical team.
- Infants born or transferred to the site during the transition period will not have their data collected and these infants will not be included in the study analysis.
- Clinical leads from each discipline (i.e. nursing and medicine) and the site research team at the site will assess for completeness of ESC care approach implementation prior to the site’s formal movement into the ESC intervention period.
- ESC experts will conduct biweekly webinars for each block of sites through the transition and initial intervention period(s). These webinars will provide continued support to the sites during this initial period of implementation, and ESC experts will continue to conduct these webinars on a monthly basis throughout the subsequent ESC intervention period(s).

#### ESC care approach

##### Intervention

- After delivery or transfer to the site, the care team will initiate non-pharmacologic care for NOWS, as detailed in the ESC training materials, and non-pharmacologic care will remain in place for the full duration of the infant’s management for NOWS.
- Non-pharmacologic care can include: primary caregiver(s) involvement (rooming-in if possible), promoting breastfeeding (for eligible infants based on the institution’s established breastfeeding guideline), encouraging on-demand feeding, enhancement of low light and minimal noise exposure, supporting clustered care (doing assessments, vitals, and all other care around feeding, to promote sleep), swaddling, and skin-to-skin care by primary caregiver(s) or holding by family/staff volunteers.
- Not all sites will be able to offer all forms of non-pharmacologic care and not all infants will be able to receive all non-pharmacologic interventions available at the site. Acknowledging this, the clinical team will make every attempt to optimize the non-pharmacologic care provided to each infant.
- The clinical team will encourage primary caregiver(s) to participate in the care and evaluation of their infants. The clinical team will also encourage the primary caregiver(s) to record the infant’s feedings (timing and duration, and/or volume), sleeping (quality and quantity), and ability to be consoled, in the Newborn Care Diary, a component of the ESC care approach.
- The clinical team, in collaboration with the primary caregiver(s), will use the ESC Care Tool to assess the infant with respect to the ESC items (eating, sleeping and consoling) by approximately 4 to 6 hours of life (if risk for NOWS is known) or upon identification of the need for NOWS management.
- The clinical team will perform ESC Care Tool assessments every 2 to 4 hours after feedings, clustering other infant and maternal care (i.e., vital signs) at the same time. These assessments will include a collaborative review with the primary caregiver(s) (when available) of the ESC items since the last assessment, using the Newborn Care Diary. If the primary caregiver(s) are not available, the clinical team who participated in the care of the infant during the assessment period will complete the assessment.
- If during an assessment the infant has a "Yes" for any ESC item or obtains a score of “3” for “Consoling Support Needed” on the ESC Care Tool, the primary caregiver(s) and clinical team will conduct a “Parent/Caregiver huddle” to determine: 1) if the "Yes" is due to NOWS and 2) which non-pharmacologic care interventions the care team can optimize further. The “Parent/Caregiver Huddle” could include, but is not limited to, the parent/caregiver and the bedside nurse.
- If the care team can optimize non-pharmacologic interventions, they will do so and will continue to assess the infant.
- If it is unclear if the infant’s difficulties with eating, sleeping, or consoling are due to NOWS, the care team will indicate a "Yes" on the ESC Care Tool and will continue to monitor the infant closely while optimizing all non-pharmacologic care interventions.
- If the infant has a second consecutive "Yes" for any ESC item (or “3” for “Consoling Support Needed”) on the ESC Care Tool (or other significant concerns are present), despite maximal non-pharmacologic care, the care team will conduct a “Full-Care Team Huddle” to determine if: 1) the "Yes" is due to NOWS and 2) the infant needs pharmacologic treatment. A “Full-Care Team Huddle” could include, but is not limited to, the parent/caregiver and the bedside nurse, in addition to the physician and/or advanced practice providers caring for the infant.
- The clinical team will initiate pharmacologic treatment if the infant scores "Yes" due to NOWS on an ESC item or scores a “3” for “Consoling Support Needed” on the ESC Care Tool despite optimization of non-pharmacologic care. If an infant requires pharmacologic treatment, sites will initiate a treatment protocol to guide care. A treatment protocol should include dose initiation, escalation, and weaning parameters. The protocol study team will provide sites with a protocol. The protocol study team will permit (following review and approval) site-level modifications of the protocol to align it with the site’s preferred practice. Opioid replacement therapy given (morphine, methadone, or buprenorphine) will be per site preference, as will adjuvant therapy (clonidine or phenobarbital).
- The clinical team will monitor each infant requiring opioid replacement therapy for signs of escalating withdrawal symptoms following discontinuation of this treatment and will consider discharge per the site’s usual practice.
- The clinical team will use the ESC Care Tool to monitor infants following birth and consider discharge for infants who do not require pharmacologic treatment per the site’s usual practice.
- To ensure fidelity of the assessments, the protocol study team will randomly assess the reliability of the “gold-star raters” at each site throughout the study period. The protocol study team anticipates that each “gold-star rater” will maintain 100% reliability in scoring. The “gold-star raters” will then assess reliability of the clinical team once per period, by assessing 10 individuals using the ESC IRR tool and written or video case scenarios on the training platform. The protocol study team anticipates that each member of the clinical team will maintain 80% reliability in scoring. If a member of the clinical team fails to meet this target during the assessment, the protocol study team will utilize just-in-time training through a centralized training platform until the clinical team member achieves 80% reliability. The protocol study team would ask that members of the care team with reliability less than 80% not be assigned to care for infants with NOWS until improved reliability is demonstrated through the just-in-time training process.
- To ensure fidelity of ESC implementation the protocol study team will develop an electronic platform that will allow “gold-star raters” to discretely evaluate, in real time, how nursing implements ESC at each participating site. The electronic platform will contain items from the ESC IRR tool and the ESC Implementation Process Evaluation. The protocol study team will use these tools to evaluate how consistent each nurse is in her/his evaluation of infant symptoms, recommendations for the care team huddle, as described by the ESC Care Tool, and implementation of the ESC Care Tool (inclusive of non-pharmacologic care interventions). The site research team will enter data into the electronic application and will send it directly to a central repository where the protocol study team will analyze the data and identify fidelity issues that the site research team can address in a timely fashion.

### Protocol adherence and compliance monitoring

The DCC will monitor protocol deviations per site in relation to the number of participants enrolled and visits conducted. All sites will receive re-education via regularly scheduled teleconferences to help other sites prevent similar deviations. If a particular deviation is recurrent at one site or across the sites, the DCC may implement operational tools, such as additional reminders, source document worksheets, and/or checklists, to reduce the likelihood of deviations. The DCC will review protocol deviations throughout the study, and it may schedule additional on-site visits, as needed, to review regulatory documents, data points, key issues, etc. or to retrain site staff to improve processes and provide additional education.

Strategies to improve or monitor adherence to the study protocol will include the following:

- Monthly recruitment reports of infants screened, enrolled, and consented (accrual figures)
- Screen fails will be reviewed by the protocol study team to assess for bias in inclusion/exclusion decisions
- Monthly reports detailing data received at the data center, data consistency, missing data, performance measures, and adherence to the study protocol (with appropriate measures taken to preserve the blinding of study personnel and investigators)
- Supplementary blinded reports requested by the study investigators or subcommittee that do not disclose allocation-group–specific outcomes (primary, secondary, or any safety outcomes)

The DCC will generate the aforementioned reports.

Additionally, the protocol study team will monitor protocol adherence through collection of the following data:

- Completion of modules and training by the research and clinical team as assessed through the education and training platform.
- Initial IRR for the clinical team (reevaluated each period).
- Assessed adherence to the assigned care approach.

### Post-hospital procedures

The site research team will assess for the outpatient composite safety outcome at approximately 3 months of age as well as the critical safety outcome through review of the medical records (including the site’s primary and any linked electronic medical record systems) and media review for all infants enrolled in the study at approximately 3 and 24 months of age. Primary caregiver(s) for infants for whom the protocol study team obtained informed consent will receive questionnaires via electronic application or via phone interview, if caregiver(s) have limited access to cellular/internet service or prefer this modality of communication. Caregiver(s) will complete these questionnaires at discharge, 1-month post discharge, and 3 months, 6 months, 12 months, and 24 months of age. These questionnaires will gather information on infant neurobehavioral functioning, infant wellness, primary caregiver(s) well-being, family environment, and caregiver-infant interactions. In addition, there will be an in-person follow-up visit with neurodevelopmental assessment and anthropometric measures at 24 months of age. The site research team will maintain contact in between study assessments at regular intervals, as detailed in Table 2. As there will likely be differences between the populations who provide consent for follow-up and those who do not, we will collect socioeconomic data (insurance and maternal educational status), marital status, and maternal receipt of medication-assisted treatment for all populations to examine for possible bias.

### Data quality assurance

To assure the quality of the data collected, the protocol study team will provide training specific to accuracy of data acquisition for the research coordinators at each site. The protocol study team will design data collection forms, which a subset of sites will subsequently pilot to minimize the potential for errors. Additionally, the protocol study team will allocate sufficient funds to allow for quality data collection. The site research team will re-abstract a subsample of their own charts and assess the error rate. Re-abstraction will focus on critical data elements related to the primary and secondary objectives of the protocol. The protocol study team will base the number of charts a site re-abstracts, for each 6-month interval, on the number of patients enrolled in the study during the 6-month period at each site as shown as outlined in Table 3.

**Table 3:** Chart Re-abstraction plan

No. of patients enrolled in a 6-month period	No. of charts to be re-abstracted
0	0
1-14	1
15-24	2
25-34	3
35-44	4
45-54	5
55-64	6

The DCC will provide sites with the randomly selected subject IDs for re-abstraction. The site research team will identify an independent site quality control (QC) abstractor who will re-abstract and enter data into the electronic data capture system (EDC) only for the QC process and will not abstract study data while QC activities are taking place. The DCC will generate a discrepancy report comparing study data abstracted by the site with the source information abstracted by the independent abstractor. The site manager will hold a QC Review Meeting with the independent site QC abstractor, research coordinator, and site abstractor(s) to review the discrepancies and identify errors. Together they will discuss and document the corrective action for each error identified. The DCC will create manual queries in the EDC to make any necessary corrections to the data that QC Review members identify. The protocol study team will provide hospitals that have an error rate above the predefined threshold with additional training, a hospital-specific assessment of the data collection process, and suggestions for process improvement. The protocol study team will track hospitals by their error rates. The protocol study team will share practices of those hospitals with exceptionally low error rates with hospitals working to improve their own process. The protocol study team will review error rates and re-abstraction data during monthly team calls. If errors exceed the predefined threshold on 2 consecutive reviews, a remediation plan will be requested and shared with the study sponsor.

Sites that have an error rate above the predefined threshold will receive additional training, a site-specific assessment of the data collection process and suggestions for process improvement. The protocol study team will highlight sites with exceptionally low error rates, and these sites will share aspects of their data collection process with sites working to improve their own process.

### Blinding/Masking

The protocol study team will assure blinding of the electronically performed follow-up questionnaires through the use of a centralized computer scoring system. For questionnaires completed by phone, each site should develop a site-specific protocol to preserve blinding of those administering the questionnaires. The protocol study team will note the method of questionnaire completion.

### Study objectives and endpoints

#### Primary outcome

The primary outcome is the time from birth until infants are medically ready for discharge. We define medically ready for discharge as when the infant meets ALL of the following criteria:

1. ≥ 96 hours of life
2. Off opioid replacement therapy (e.g. morphine, methadone, or buprenorphine) for ≥ 48 hours
3. Taking 100% of feeds by mouth for ≥ 24 hours
4. ≥ 24 hours from initiation of the maximum caloric density infant received during the initial hospital admission
5. Receiving no respiratory support for ≥ 24 hours
  Hypothesis: Among infants evaluated for NOWS, the ESC care approach will reduce the length of time until infants are medically ready for discharge by an average of 4 days, compared to usual institutional care with the FNAST or modification thereof.

#### Secondary outcomes

##### Obtained Under Waiver of Consent and Gathered by Authorized Site Research Personnel from the On-Site Medical Records, Linked Medical Records and Research Forms

1. Receipt of opioid replacement therapy (morphine, methadone, or buprenorphine) for NOWS prior to hospital discharge
  Hypothesis: The use of the ESC care approach will decrease the proportion of infants who receive opioid replacement therapy.
  This is a yes/no outcome, and it will enable us to determine the percentage of infants receiving opioid replacement therapy in each intervention group.
2. Total postnatal opioid exposure prior to hospital discharge
  Hypothesis: The use of the ESC care approach will decrease total opioid exposure, compared to usual care.
  Each dose of opioid replacement therapy (total units and units/kg and morphine equivalents [mg/kg]) that infants received throughout the initial birth hospitalization will be collected to determine total postnatal opioid exposure.
3. Hour of life opioid replacement initiated
  Hypothesis: The use of the ESC care approach will not delay the initiation of pharmacologic therapy.
  Use of the ESC Care Tool for the assessment of infants may delay the initiation of pharmacologic therapy and thus infants may be at an advanced state of withdrawal and more difficult to “capture”. Alternatively, there is some evidence to suggest27 that use of the ESC Care Tool ultimately allows for more timely recognition of infants requiring pharmacologic therapy, compared to usual care using the FNAST.
4. Receipt of adjuvant therapy (clonidine or phenobarbital) prior to hospital discharge
  Hypothesis: The use of the ESC care approach will decrease the proportion of infants who receive adjuvant therapy.
  This is a yes/no outcome, and it will allow us to determine the percentage of infants receiving adjuvant therapy.
5. Maximum percent weight loss during the initial birth hospitalization
  Hypothesis: Use of the ESC care approach will not result in more excessive weight loss than usual care.
  Poor feeding and excessive weight loss are signs of suboptimal control of NOWS. Birth weight and daily weights (g) will be collected throughout the initial birth hospitalization to determine the impact of NOWS on growth, and the maximum percent weight loss will be calculated as:
    $$\left[\frac{birthweight\ (g)- weight\ nadir(g)}{birthweight\ (g)}\right]x\ 100=\max percent\ weight\ loss$$
6. Type of enteral feedings (exclusive maternal breastmilk, combination of formula and maternal breastmilk, exclusive formula feeding) at time of hospital discharge
  Hypothesis: Use of the ESC care approach will increase the proportion of infants who receive maternal breastmilk at the time of discharge from the initial birth hospitalization.
  Studies have shown that the receipt of maternal breastmilk decreases withdrawal signs in infants in a dose-dependent fashion [38, 39]
  The site research team will assess and collect the type of enteral feeding at the time of discharge from the initial birth hospitalization.
7. Direct breastfeeding at the time of hospital discharge
  Hypothesis: Use of the ESC care approach will increase the proportion of mothers who directly breastfeed at the time of discharge from the initial birth hospitalization.
  The site research team will assess and collect direct breastfeeding occurrences within 24 hours of the time of discharge from the initial birth hospitalization.
8. Length of hospital stay
  Hypothesis: Infants managed with ESC will have a decrease in the LOS.
  The site research team will report the LOS in addition to the length of time until infants are medically ready for discharge. The differences in these measures will allow the protocol study team to assess the impact of social factors on the length of hospitalization.
9. A composite measure of infant safety during the initial birth hospitalization (seizures, accidental trauma [i.e., dropped infants], and respiratory insufficiency due to opioid therapy, including documented apnea or need for respiratory support [positive pressure or supplemental oxygen])
  Hypothesis: Infants managed using the ESC care approach will be safe during the initial birth hospitalization.
  Use of the ESC care approach may delay initiation of pharmacologic therapy, which could result in an increase in withdrawal-related seizures. Therefore, monitoring for the presence or absence of seizures will help to build the safety profile for ESC.
  Increased primary caregiver(s) involvement is thought to result from the ESC care approach. In this case, parent/caregiver skin-to-skin time and holding may increase, which could increase the risk of infants being dropped if primary caregiver(s) are fatigued and/or chemically impaired.
  Use of the ESC care approach may delay initiation of pharmacologic therapy, which could result in the infant receiving a higher dose of opioid replacement therapy. Higher doses of opioids may increase the risk of respiratory insufficiency. Therefore, monitoring for respiratory insufficiency will help to build the safety profile for ESC.
10. A composite measure of critical infant safety outcomes during the initial birth hospitalization (non-accidental trauma and death)
  Hypothesis: Infants managed using the ESC care approach will be safe during the initial birth hospitalization.
  Use of the ESC care approach encourages parents/caregivers to provide extensive non-pharmacologic care and rooming-in. This may increase stress and fatigue and lead to undesired caregiver-infant interactions. Inclusion of a critical composite safety outcome inclusive of non-accidental trauma and death will help to build the safety profile for ESC
11. A composite measure of infant safety during the first 3 months of life based on the presence or absence of acute/urgent care and/or ER visits and hospital readmissions
  Hypothesis: Infants managed using the ESC care approach will be safe during the first 3 months of life.
  Discharge of an infant earlier from the initial hospitalization and/or increased primary caregiver involvement during the initial hospitalization may increase the stress and fatigue experienced by the caregiver(s) and lead to increased risk for poor outcomes, and increased healthcare utilization.
12. A composite measure of critical safety outcomes based on the presence or absence of non-accidental trauma and death at discharge and during the first 3 and 24 months of life
  Hypothesis: Infants managed using the ESC care approach will be safe during the first 3 and 24 months of life.
  Infants with undertreated signs of withdrawal may be at increased risk for non-accidental trauma and death due to the potential for increased primary caregiver stress and fatigue during the hospital admission and following discharge. These infants may also fail to develop a bond with their primary caregiver(s) during the first months of life, which may further increase the risk for non-accidental trauma and death during the first two years of life.

##### Obtained for the subpopulation who provide informed consent and acquired through questionnaires

Assessed at various time points between discharge and 24 months of age (see Table 2).

1. Infant neurobehavioral functioning following discharge

- Hypothesis: Infants managed using the ESC care approach will have improved infant neurobehavioral functioning when compared to usual care.
- Assessed with Infant Behavior Questionnaire - Revised (IBQ-R) very short form at 3 and 12 months of age. The caregiver will complete the survey and it will be sent to a central location for review by the protocol study team
  - The IBQ–R is a well-established caregiver report measure of neurobehavioral functioning through assessment of temperament for infants between 3 and 12 months of age [40] The questionnaire has demonstrated good internal consistency, reliability, and validity [41–44] The IBQ-R consists of 191 items and takes approximately 1 hour to complete which makes it impractical for this study. The very short form consists of 37 questions that measure surgency, negative affect, and effortful control of the infant caregiver. This form takes approximately 12 minutes to complete.46 The very short form has been shown to have reliability and stability that are similar to the IBQ–R and other temperament measures [45]

2. Infant wellness following discharge as independently assessed by:

- Anthropometric growth (weight, height, head circumference)
  - Hypothesis: Use of the ESC care approach will not impact growth long-term when compared to usual care.
  - Assessed with percentile measurements of weight, length, head circumference (HC), and weight for length on WHO growth curves. The research team will assess weight, length, head circumference and weight for length at hospital discharge and 24 months of age. The study team will calculate anthropometric z-scores at these time points, and will assess BMI at 24 months of age and calculate BMI-z.
- Sleep
  - Hypothesis: The infant’s sleep will improve after use of the ESC care approach compared to usual care.
  - Assessed with the Brief Infant Sleep Questionnaire (BISQ) [46] at 3 and 12 months of age. The caregiver will complete the survey and it will be sent to a central location for review by the protocol study team.
- Enteral feeds during the first 6 months of life (exclusive maternal breastmilk, combination of maternal breastmilk and formula, or exclusive formula feeding)
  - Hypothesis: Use of the ESC care approach will increase the proportion of infants who receive maternal breastmilk following discharge compared to usual care.
  - Assessed with the Caregiver Questionnaire (CQ) at 1-month post hospital discharge, and 3, and 6 months of age. The caregiver will complete the questionnaire and it will be sent to a central location for review by the protocol study team.
- Direct breastfeeding during the first 6 months of life
  - Hypothesis: Use of the ESC care approach will increase the proportion of mothers who directly breastfeed following discharge compared to usual care.
  - Assessed with the CQ at 1-month post hospital discharge, and 3, and 6 months of age. The caregiver will complete the questionnaire and it will be sent to a central location for review by the protocol study team.
- Number of ER visits and/or acute/urgent care visits
  - Hypothesis: Use of the ESC care approach will not result in an increase in the number of ER or acute/urgent care visits compared to usual care.
  - Assessed at 1-month post hospital discharge, and 3, 6, 12, and 24 months of age via completion of the CQ and submission for review by a protocol study team. The site research team will also assess the site’s electronic health record (EHR) and include any visits not reported, if observed.
- Readmissions
  - Hypothesis: Use of the ESC care approach will not result in an increase in the number of readmissions following initial hospital discharge compared to usual care.
  - Assessed at 1-month post hospital discharge, and 3, 6, 12, and 24 months of age via completion of the CQ and reviewed by the protocol study team. The site research team will also assess the sites’ EHR and include any visits not reported, if observed.

3. Maternal/caregiver well-being

- Hypothesis: Use of the ESC care approach will improve maternal/caregiver well-being compared to usual care.
- Assessed with Patient Reported Outcomes Measurement Information System (PROMIS) short forms at discharge, 6 months and 24 months [47]. Standardized short forms examining mental health, specifically the areas of anxiety (PROMIS Short Form v1.0 - Anxiety - 8a 31May2019), depression (PROMIS_SF_v1.0_-_ED-Depression_8a_5-31-2019), anger (PROMIS Short Form v1.1 - Anger - 5a 27Apr2016), life meaning and purpose (PROMIS Short Form v1.0 - Meaning and Purpose - 8a 18Jul2017), and social support (PROMIS v2.0 - Emotional Support Short Form 4a 23June2016) will be completed by the primary caregiver and will be sent to a central location for review by the protocol study team.
- The standardized short form for each of the PROMIS Measures consists of between four to eight 5-point Likert scale questions. The PROMIS Depression Short form has been validated in the postpartum period and has been found to be strongly correlated with the Edinburgh Postnatal Depression Scale, the most extensively studied measure of depression in the postpartum period [48, 49]. In addition, the PROMIS anxiety measure has been correlated with the Mood and Anxiety Questionnaire (MASQ) and has been shown to be a valid measurement tool for anxiety in the post-partum period in a sample of parents whose infants were hospitalized in the NICU [49]. Administration takes approximately 10 minutes and includes a total of 33 questions.

4. Infant-caregiver bonding and attachment

- Hypothesis: Use of the ESC care approach will result in improved infant-caregiver bonding and attachment, compared to usual care.
- The protocol study team will assess with the Maternal Postnatal Attachment Questionnaire (MPAQ) at discharge and 6 months of age. The caregiver will complete the questionnaire and it will be sent to a central location for review by the protocol study team.
- Primary caregiver-infant interactions will be assessed with the MPAQ, [50] a 19-item questionnaire that assesses quality of bonding, absence of hostility, and pleasure in interaction. The MPAQ requires approximately 5 minutes to complete, and researchers have validated the tool for postpartum women with substance-abuse problems [51]
- The focus of the MPAQ is primarily upon the caregiver(s) subjective experiences in relation to their infant in the first year of life [52] Established risk quartiles exist, and the protocol study team will note caregiver(s) for entry and exit into these high-risk quartiles at each time point.

5. Parenting efficacy

- Hypothesis: Use of the ESC care approach will result in improved caregiver sense of competency in caring for their infants compared to usual care.
- The protocol study team will assess with the Parenting Sense of Competence (PSOC) Scale at discharge and 6 months of age. The caregiver will complete the questionnaire and it will be sent to a central location for review by the protocol study team.
- The PSOC is a self-reporting instrument that measures and assesses parent self-efficacy. It is a 17-item publicly available scale that measures satisfaction (degree of liking a person has for their role as a parent) and efficacy (an individual’s perceived competence in their role as a parent).
- Researchers have used this tool to assess the impact of parenting efficacy on the likelihood of out-of-home placement and loss of custody in mothers with mental health and substance use disorders [53]

6. Family environment

- Hypothesis: Use of the ESC care approach will enhance family environment when compared to usual care.
- The protocol study team will assess with Family Environmental Scale (FES) - Relationship Dimension - Form R at 3 months of age. The caregiver will complete the questionnaire and it will be sent to a central location for review by the protocol study team.
- The Relationship dimension of the FES consists of the Cohesion, Expressiveness, and Conflict subscales. Form R for each subscale is composed of 9 true-false items.
- The relationship dimension assesses the degree of commitment, help, and support that family members provide each other, the extent to which family members are encouraged to act openly and to express their feelings directly, and the amount of openly expressed anger, aggression, and conflict among family members [54]
- Researchers frequently use the FES to assess the home environment and it has been found to have strong psychometric properties [54]

7. Influence of maternal childhood experiences on infant outcomes

- Hypothesis: Maternal history of adverse childhood experiences will be associated with worse infant behavioral functioning and developmental outcomes.
- The protocol study team will assess adverse childhood experiences using the Adverse Childhood Experience (ACE) Questionnaire at 24 months of age.
  - The ACE [55] is a self-report measure used to capture specific childhood experiences correlating with future social risk factors and negative health outcomes.

##### Obtained for the subpopulation who provide informed consent and acquired through an in-person visit at 24 months of age

1. Infant development

- Hypothesis: Use of the ESC care approach will improve infant development, compared to usual care.
- The protocol study team will assess infant development with the Bayley Scales of Infant and Toddler Development, Fourth Edition (Bayley-4): Cognitive, Language and Motor at 24 months of age. The Bayley-4 will be administered by a trained examiner blinded to neonatal history who has undergone extensive training and been certified to perform exams.
  - The Bayley Scales are recognized internationally as one of the most comprehensive tools to assess developmental outcomes in children. With the Bayley-4, it is even possible to obtain detailed information from non-verbal children as to their functioning. Children are assessed with 3 key developmental domains: cognition, language and motor. Reliability and validity of the previous version of the instrument have been well established [56]
- The protocol study team will assess infant behavioral development with the Brief Infant-Toddler Social and Emotional Assessment (BITSEA) at 24 months of age. The BITSEA is a standardized and normed referenced instrument designed to assess for social-emotional, behavior concerns, and social competence in infants-toddlers [57].
  - The 42-item parent rating form is a shorted version of the Infant-Toddler Social and Emotional Assessment. Scores include ratings of internalizing behaviors, externalizing behaviors, executive function (dysregulation), psychosocial competence, social relatedness; maladaptive, and atypical behavior. The behavioral indices included in the BITSEA have been observed to correspond with neurodevelopmental functioning among infants at risk for neurodevelopmental problems.

### Potential risks and benefits to participants

Under the proposed study design, the protocol study team will randomize each site into blocks with each block transitioning from usual care to ESC at a randomly allocated time. At any given time during the study enrollment period, all infants managed for NOWS at a site will receive care consistent with the care approach assigned by the protocol study team. Sites throughout the country are currently using both care approaches described in this study for the evaluation and management of NOWS. Use of either care approach will not expose infants in this study to risk beyond that of usual/accepted clinical care.

Involvement in the study will not increase the risk to the family of legal ramifications associated with the in utero opioid exposure of their infants, as only infants who have been identified by the site as at risk for NOWS and for whom management for NOWS has begun, will be screened for enrollment in the trial. There will be no additional toxicology screening (maternal or infant) performed beyond what medical professionals would typically obtain as part of usual institutional care at the site. Thus, there will be no additional information garnered with respect to substance use during pregnancy due to one’s involvement in the trial.

The protocol study team will assess primary caregiver well-being (e.g. parenting stress, attachment and bonding, depression, anxiety, etc.) as well as infant well-being, neurobehavioral functioning, and development during the follow-up portion of the study.

The protocol study team will assess primary caregiver wellness with 5 PROMIS Measures. It is possible that these questionnaires may reveal that the primary caregiver is experiencing psychological distress potentially requiring support. The study team has determined that a standardized scoring threshold for the PROMIS Depression Measure will be used to identify these individuals. As thresholds specific to postpartum women with opioid dependency have yet to be established and given that severe depression (a t-score >70, or 2 standard deviations above the mean for the normative population is the threshold for severe depressive symptoms [58, 59]) is most likely to impact family well-being, a score of >70 was chosen for this threshold.

If a primary caregiver has a t-score >70 on the PROMIS Depression measure, the primary caregiver will be provided with national hotline support numbers within the electronic questionnaire platform. In addition, after the questionnaire is completed in REDCap an email will be automatically generated and sent to the study coordinator and PI. Each site will develop a plan to provide support for the primary caregivers at risk and connect them with local mental health resources in response to those emails. The protocol study team will collect a copy of this plan from each site.

SAMHSA NATIONAL HELP LINE – 1-800-662-4357 (HELP)

- https://www.samhsa.gov/find-help/national-helpline
- SAMHSA’s National Helpline is a free, confidential, 24/7, 365-day-a-year treatment referral and information service (in English and Spanish) for individuals and families facing mental and/or substance use disorders.

NATIONAL DOMESTIC VIOLENCE HOTLINE - 1−800−799−7233 (SAFE)

- https://www.thehotline.org
- Advocates are available 24/7/365 to talk confidentially with anyone experiencing domestic violence, seeking resources or information, or questioning unhealthy aspects of their relationship.

NATIONAL SUICIDE PREVENTION LIFELINE – 1-800-273-8255

- https://suicidepreventionlifeline.org
- The National Suicide Prevention Lifeline is a national network of local crisis centers that provides free and confidential emotional support to people in suicidal crisis or emotional distress 24 hours a day, 7 days a week.

Additionally, a response plan will be in place at each site for questions specific to incidental findings of or suspicions for child abuse and/or neglect.

Participants recognized to have neurodevelopmental impairment on the Bayley-4 exam will be referred to their primary care providers for follow-up. The study team will communicate and share the report with the caregiver(s) and primary care providers if requested by the participants’ caregiver(s) and consent is obtained.

The infants in the study may not benefit directly from participation. There may be a benefit to the infant of information garnered from the developmental screening portion of the study. By virtue of inclusion in a research study, participants are at risk of loss of confidentiality of medical-record information because participants will have their medical records reviewed by research personnel. The protocol study team will institute measures to protect the privacy of medical information, including the coding of all HIPAA (Health Insurance Portability and Accountability Act) identifiers in medical records, limitation of access to the medical records to research personnel, and removal of any individual identifiers in reports and publications generated from the study. Research personnel will keep any hard copies of research records in a locked cabinet and will destroy these records after the study is complete and the protocol study team publishes the results. In this study, infants themselves are the primary research focus, thus justifying the inclusion of children. The protocol study team will not exclude a subject based on race, ethnicity, or gender. However, some of the study questionnaires that will be used have not been validated in languages other than English. Thus, the population for the consented portion of the study will be limited to infants of English-speaking, reading and writing caregivers. Due to the demographic distribution of NOWS, the proportion of low socioeconomic-status infants will likely be higher than in the general population.

### Recruitment and retention

#### Site recruitment and retention

The protocol study team began to optimize the potential for recruitment during initial protocol development, through an assessment of potential ISPCTN and NRN sites’ willingness to participate in and enthusiasm for various study designs. The study design chosen for this protocol incorporates the feedback from these sites. During the site assessment process, the protocol study team will expect each site to commit in writing to the site’s participation in and completion of the trial with maintenance of the site’s allocated intervention for the duration of the study. The protocol study team will facilitate retention of sites through the focused allocation of funds to support participation, through assessment of needs, provision of support, and troubleshooting at each site, as needed.

#### Infant and parent/caregiver recruitment and retention

The site research teams will need to obtain participant consent for the long-term follow-up portion of the study. Historically, enrollment of infants with NOWS in clinical trials that seek to improve their care has been challenging. In response to this, the protocol study team plans to utilize a robust recruitment and retention plan developed to support and optimize the participation of this population in the follow-up portion of this study.

##### Recruitment

The single most important element of the recruitment strategy is to establish trust with the primary caregiver(s) and provide an introduction to the research plan prior to delivery. The prenatal consultation is most likely the first time that the family will meet the site PI or designee and is an ideal time to introduce the trial. The consultation is the opportunity for the provider to gain trust with the family and reaffirm a partnership with the family. The consultation will include establishing a foundation of knowledge about NOWS, outlining gaps in current national care, and a detailed description of the research approach.

In anticipation that prenatal consultations will not be feasible for all patients, effective dissemination of information regarding the clinical trial will be exceptionally important. The protocol study team will provide an informational pamphlet to all parent/caregiver(s) of infants receiving care for NOWS at participating sites soon after delivery. The consenting member of the site research team will begin trust building with the parent/caregiver(s) in anticipation of the consenting process. The site research team will present information about the study in person, and/or via an informational brochure developed by the protocol study team and distributed to the sites. To further optimize recruitment, if informed consent is not able to be obtained during the initial hospitalization, it is permissible to obtain consent up to one month after discharge.

The protocol study team will assess site recruitment for long-term follow-up each month following site enrollment. If the protocol study team assesses the site as below target, the study team will evaluate the site’s processes for recruitment, and the site will receive additional training and/or modifications to the recruitment approach as suggested by a recruitment and retention expert from the protocol study team.

Additionally, the protocol study team will assess for barriers to participation, perceived or actual, of non-consenters and utilize their responses to further improve site-specific and study-wide recruitment strategies.

##### Retention

The protocol study team will optimize infant and parent/caregiver(s) retention soon after a site obtains consent, by sending a note of thanks to the parent/caregiver(s) and acknowledging the importance of their contribution to the future care we provide these infants. The site research team will further optimize retention via text messaging for reminders, and access to questionnaires through a centrally located electronic platform. The site research team will use the electronic health record to update a participant’s contact information as needed, in the event that the contact information provided by the participant is not sufficient. The site research team can conduct questionnaires via phone interview if the caregiver(s) has limited access to cellular/internet service or prefer this modality of communication. If a participant answers questions for questionnaires or comes to an in-person visit, the parent/caregiver(s) will receive compensation for their time. This compensation will be provided at – or very near – the time the participant finishes that contact time. Participants will be reimbursed for their time according to the plan outlined in Table 4.

**Table 4:** Participant Reimbursement Plan

Contact Time	Participated in	Reimbursement/ Compensation Amount
Hospital Discharge	Answering Questionnaires	$50.
1-month post discharge (of baby from hospital)	Answering Questionnaires	$50.
Baby 3 months of age	Answering Questionnaires	$50.
Baby 6 months of age	Answering Questionnaires	$50.
Baby 12 months of age	Answering Questionnaires	$50.
Baby 24 months of age	Bringing baby in for in-person Bayley’s exam and answering questionnaires	$100.

The mechanism of payment (gift card, check, etc.) will be site specific and will be according to each site’s mechanism for making such payments.

The site research team will provide text reminders to the parent/caregiver(s) to optimize timely completion of the questionnaires. Additionally, the site research team at each site will include a retention coordinator, and the protocol study team will allocate funds to support this role.

Additionally, the protocol study team will explore other methods to optimize both recruitment and retention. This could include, but is not limited to, discussions with stakeholders and parent/caregiver(s) from the community who have had infants treated for NOWS and understand the importance of being able to successfully complete this trial.

## Analytical plan

### Statistical analysis plan

#### General approach

The material of this section is the basis for the statistical analysis plan of this study. The protocol study team may revise the plan during the study to accommodate clinical trial protocol amendments and to make changes to adapt to unexpected issues in study execution and data that affect planned analyses. The protocol study team will conduct all statistical analyses following the statistical principles for clinical trials as specified in International Council on Harmonization Topic E9. The protocol study team will describe and justify any deviations from the planned analyses in the final integrated clinical study report. The protocol study team will present overall and study site-specific data and summary tables.

The protocol study team will present the characteristics of infants and mothers by intervention groups (usual care versus ESC care approach) and their outcomes for each site. We do not expect significant differences in the demographics of the study population during the 20-month study period. Each site covers a different population mix, and while each hospital will contribute both usual care and ESC participants, they will do so in different proportions depending on when the protocol study team randomizes the hospital to the intervention. This will contribute greatly to any demographic differences between the usual institutional care and ESC groups. Whilst we do not intend to test for demographic differences between the usual institutional care and ESC groups for the full cohort, we will adjust the analyses for the covariates described because of potential imbalance across sites and across steps. We will present numerical variables as means [standard deviation (SD)] or medians (interquartile range), depending on their distribution, and categorical variables as counts and percentages.

We will use the principles of intention-to-treat for all statistical analyses related to primary and secondary endpoints.

#### Analysis of the primary efficacy endpoints

For the primary efficacy variable, we will test the following null hypothesis:

H0: There is no treatment difference in average length of time until medically ready for discharge between usual care and the ESC care approach.

##### Versus

H1: There is a treatment difference in average length of time until medically ready for discharge between usual care and the ESC care approach.

We will consider the length of time until medically ready for discharge measure a count measure and has the potential to follow a skewed distribution. Initially, we will assess the distributional assumption. We will evaluate the associations of potential confounders (e.g., gestational age, birth weight, race/ethnicity, hospital volume, rural/urban indicator) at both the participant and site level with the intervention. An additional potential confounder that we will evaluate will be the presence of other ongoing clinical trials in our trial sites that might impact the outcome of this study, including the “Prospective Randomized Blinded Trial to Shorten Pharmacologic Treatment of Newborns with Neonatal Opioid Withdrawal Syndrome (NOWS)”.

We will use a generalized linear mixed-effects model (GLMM) to compare the expected length of time until medically ready for discharge between the two treatment interventions (usual care and ESC care approach). Specifically, we will use a GLMM with a negative binomial distribution and log-link to account for potential over-dispersion, as an infant level analysis, and accounting for correlations between observations in the same hospital by including hospital in the model as a random effect. We will report point estimates for the group mean difference along with a 95% confidence interval (CI). The model-building approach for our primary outcome will follow four analyses steps: 1) an unadjusted before/after of the effect of the ESC care approach (ignoring period/time effect); 2) the time period (i.e., steps) to examine if any potential intervention effect relates only to the intervention or also to an independent effect of calendar time; 3) an adjustment for infant-level and maternal characteristics and potential hospital-level confounders, such as hospital volume and rural/urban indicator; 4) the possible interaction between period and intervention effect. The impact of the ESC care approach on the primary outcome could potentially change over time, as the improvement in outcome could increase with time as the staff gains experience. However, the impact could also decrease after an initial improvement as the level of initial enthusiasm decreases. We aim to explore this question through the inclusion of an interaction between period/time and intervention effect in Model 4.

In certain circumstances, medical personnel may discharge an infant prior to being medically ready for discharge as defined in our protocol (e.g., sent home on opioids such as methadone, morphine, or buprenorphine). Therefore, to compare the 2 interventions based on the primary outcome, we will censor these infants. Since one can view the time until medically ready for discharge as a time-to-event outcome, we will use the log-rank test adjusted for a cluster randomized design to compare the median time the infant is medically ready for discharge between the intervention groups [60]. Additionally, we will use a Cox proportional hazards (Cox PH) model with the Lin and Wei robust sandwich estimate of the variance-covariance matrix, to account for clustering, to adjust for infant and maternal demographics. We expect the amount of censoring to be minimal, therefore the results from the Cox PH model will serve as a sensitivity analysis for our primary analysis based on a GLMM with log-link.

#### Analysis of secondary endpoints obtained under waiver of consent

##### Receipt of opioid replacement therapy (Morphine, Methadone, or Buprenorphine) for neonatal opioid withdrawal syndrome prior to hospital discharge

The analysis team will compare the proportion of receipt of opioid replacement therapy for NOWS prior to hospital discharge between the intervention groups using a GLMM with a logistic link function. We will follow the same four modeling strategies described for the primary outcome. We will also present odds ratio estimate of receipt of opioid replacement therapy for NOWS for the intervention effect (ESC versus usual institutional care) with 95% CI.

##### Total opioid exposure prior to hospital discharge

The analysis team will provide the median and range of the total opioid exposure prior to hospital discharge for each treatment group. For the unadjusted analysis, the team will compare the median opioid exposure of the treatment groups using the Wilcoxon rank-sum test for clustered data proposed by Rosner, Glynn, and Lee (2003) [61]. Their test statistic extends the Wilcoxon rank-sum test under the assumptions that all participants from the same cluster belong to the same treatment group, that observations within any cluster are exchangeable, and that the intracluster dependence does not vary across treatment groups. Additionally, the team will use median mixed regression to account for the potential skewness of maximum dose of opioid replacement therapy and of clustered data and allow adjustment for covariates. The team will use the same four model building sequence described for the primary outcome except that the team will replace GLMM with a median mixed regression model.

##### Hour of life opioid replacement initiated

The analysis team will provide median and range for the hour of life when opioid replacement was initiated, and will do so separately for each treatment group. We anticipate that most of the infants will not receive opioid replacement, therefore we will use a hurdle model to model the expected hour of life until medical personnel initiate opioid replacement (i.e., count data) while handling excess zeros and over dispersion. More specifically, the team will fit the first part of the model with a binary logit model, which models whether an infant receives opioid replacement or not. In the second part, the team will utilize a negative binomial mixed model to account for the stepped-wedge design and adjust for potential infant and maternal demographics.

##### Receipt of adjuvant therapy (clonidine or phenobarbital) prior to hospital discharge

The analysis team will compare the proportion of receipt of adjuvant therapy between the treatment groups using a GLMM with a logistic link function. We will follow the same four modeling strategies described for the primary outcome. We will present odds ratio estimate of receipt of adjuvant therapy for the intervention effect (ESC versus usual institutional care) with 95% CI.

##### Maximum percent weight loss during birth hospitalization

The analysis team will provide the mean and SD of percent weight loss during birth hospitalization separately for each treatment group. The team will use a GLMM with an identity link function to compare average percent weight loss between the ESC care approach versus usual institutional care. The analysis team will report point estimates for the group mean difference along with a 95% CI. The team will use the same four model building sequence described for the primary outcome.

##### Type of enteral feedings (exclusive maternal breastmilk/breastfeeding, combination of maternal breastmilk and formula, exclusive formula feeding) at time of hospital discharge

The analysis team will compare the proportion of infants receiving any maternal breastmilk (i.e., exclusive breastmilk/breastfeeding or combination) at discharge between the treatment groups using a GLMM with a logistic link function. We will follow the same four modeling strategies described for the primary outcome, and we will present the odds ratio estimate of receiving any maternal breastmilk for the intervention effect (ESC versus usual institutional care) with 95% CI.

##### Breastfeeding at the time of hospital discharge

The analysis team will compare the proportion of breastfeeding at the time of hospital discharge between the treatment groups using a GLMM with a logistic link function. We will follow the same four modeling strategies described for the primary outcome. We will present odds ratio estimate of breastfeeding at the time of hospital discharge for the intervention effect (ESC versus usual institutional care) with 95% CI.

##### Length of hospital stay

Similar to the primary outcome (i.e., length of time until medically ready for discharge measure), we will consider LOS a count measure. Therefore, we will complete the analysis of LOS using a GLMM with log link assuming a negative binomial distribution to account for over-dispersion. The protocol study team will report point estimates for the group mean difference along with a 95% CI. Similar to the primary analysis, we will start with an unadjusted analysis and conclude with a model that includes possible interaction between period and intervention effect.

##### Composite measure of infant safety during birth hospitalization (seizures, accidental trauma [i.e., dropped infants], respiratory insufficiency due to opioid therapy, including documented apnea or need for respiratory support [positive pressure or supplemental oxygen])

We will be monitoring for the presence or absence of safety indicators such as seizures, accidental trauma, and respiratory insufficiency due to opioid therapy. To assess the safety concerns of the ESC care approach, we will create a binary composite measure of inpatient infant safety. The binary composite measure will have a value of 1 if there is a presence for any inpatient infant safety indicator and 0 otherwise. We will compare the proportion of positive inpatient safety concerns between the treatment groups using a GLMM with a logistic link function. We will follow the same four modeling strategies described for the primary outcome, and we will present odds ratio estimate of inpatient safety concerns for the intervention effect (ESC versus usual institutional care) with 95% CI.

##### Composite measure of infant safety during the first 3 months of life (acute/urgent care and/or ER visits and readmissions)

To assess the safety concerns of the ESC care approach, we will create a second composite measure consisting of outpatient infant safety indicators. We will base this outpatient composite measure on the presence or absence of acute/urgent care and/or ER visits, or readmissions during the first 3 months of life. Similar to the inpatient composite safety measure, we will compare the proportion of positive outpatient safety concerns between the treatment groups using a GLMM with a logistic link function. We will follow the same four modeling strategies described for the primary outcome, and we will present odds ratio estimate of outpatient safety concerns for the intervention effect (ESC versus usual institutional care) with 95% CI.

##### Composite measure of critical safety outcomes during the first 24 months of life (non-accidental trauma and death)

The analysis team will compare the proportion of non-accidental trauma and death between the treatment groups using a GLMM with a logistic link function. We will follow the same four modeling strategies described for the primary outcome, and we will present odds ratio estimate of non-accidental trauma and death for the intervention effect (ESC versus usual institutional care) with 95% CI.

#### Analysis of the long-term outcome endpoints obtained under provision of consent

##### Growth assessed with respect to weight, length, head circumference, and weight-for-length normalized to world health organization growth curves

We will calculate anthropometric z-scores at each assessment period for the purpose of analysis based on age- and gender-specific WHO norms. The analysis team will provide the mean and SD of infants' weights (z-scores) separately for each treatment group. The team will use a GLMM with appropriate link function (i.e., identity link for continuous outcome) to evaluate the effect of ESC on weight (z-scores). The model will examine the how the treatment means differ (i.e., main treatment effect), how treatment means change over time (i.e., main time effect), and how differences between treatment means change over time (i.e., treatment-by-time effect). The team will carry out assessment across 2 time points: hospital discharge and 24 months of age. The GLMM analytical approach allows us to analyze correlated data obtained repeatedly from the same participant and account for the ICC among participants nested within with same clinical site. To account for potential imbalance in key demographic and site-level characteristics, the analysis team will utilize both unadjusted and adjusted GLMMs. Initially, the unadjusted GLMM will include the fixed categorical effects of intervention, time, and intervention-by-time interaction and the random-site effect. We will calculate the point estimates and their respective CIs for the changes in infants' weights for each intervention group and for the difference in the estimated change between intervention groups. Additionally, the team will present the p-value of the difference in point estimates between intervention groups.

The analysis team will examine the impact of the ESC care approach on length, head circumference, and infant weight for length (z-scores) using the same analytical methods described for weight (z-scores). Additionally, the team will provide the mean and SD of infant BMI-z at 24 months for each treatment group. The team will use a GLMM with an identity to compare average BMI-z between the groups, and the team will report point estimates for the group mean difference along with a 95% CI.

##### Sleep assessed with the brief infant sleep questionnaire (BISQ)

The analysis team will provide the mean and SD of the BISQ survey separately for each treatment group. The team will use a generalized linear mixed model (GLMM) with appropriate link function (i.e., identity link for continuous outcome) to evaluate the effect of ESC on infant sleep duration. The model will examine how the treatment means differ (i.e., main treatment effect), how treatment means change over time (i.e., main time effect), and how differences between treatment means change over time (i.e., treatment-by-time effect). The team will carry out assessment at 3 months and 12 months of age. The GLMM analytical approach allows us to analyze correlated data obtained repeatedly from the same infant and account for the intracluster correlation coefficient among infants nested within with same clinical site. To account for potential imbalance in key demographic and site-level characteristics, the analysis team will utilize both unadjusted and adjusted GLMMs. Initially, the unadjusted GLMM will include the fixed categorical effects of intervention, time, and intervention-by-time interaction and the random-site effect. We will calculate the point estimates and their respective CIs for the changes in infants' BISQ scores for each intervention group and for the difference in the estimated change between intervention groups. Additionally, the team will present the p-value of the difference in point estimates between intervention groups.

##### Enteral feeds during the first 6 months of life

We will measure enteral feeds on a nominal scale (i.e., exclusive maternal breastmilk, combination of maternal breastmilk and formula, or exclusive formula feeding). The analysis team will tabulate count and relative frequency for each level and for each treatment group. To evaluate the association between enteral feeds with intervention, the team will use a mixed-effects multinomial logistic regression model to account for the longitudinal cluster study design and potential participant and site-level covariates.

##### Breastfeeding during the first 6 months of life

The analysis team will report the proportion of direct breastfeeding for each treatment group during each of the assessment periods (1 month post-discharge, and 3 months and 6 months of age). To evaluate the association between breastfeeding with intervention, the team will use a mixed-effects logistic regression model to account for the longitudinal cluster study design and potential participant and site-level covariates. We will present odds ratio estimate of breastfeeding at each assessment period for the intervention effect (ESC versus usual institutional care) with 95% CI.

##### Number of emergency room visits and/or acute/urgent care visits

The analysis team will examine the impact of the ESC care approach on the reduction of ER visits and/or acute/urgent care visits using the same analytical steps described for the primary outcome. Given that the outcome measure is count (number of ER visits, integers ≥ 1), we expect that Poisson regression analysis, adjusted for clustering at hospital will be appropriate. However, if the distribution should be approximate to normal or if the team observes over-dispersion, we will consider linear mixed-effect regression or negative binomial models. Again, the team will use the same four model building sequence described for the primary outcome. Specifically, we will start with an unadjusted model and conclude with a model that will include possible interaction between period and intervention effect.

##### Readmissions

The analysis team will compare the proportion readmissions between the treatment groups using a GLMM with a logistic link function. We will follow the same four modeling strategies described for the primary outcome, and we will present odds ratio estimate of readmissions for the intervention effect (ESC versus usual institutional care) with 95% CI.

##### Patient-reported outcomes measurement information system (PROMIS) short forms

The analysis team will measure primary caregiver(s)’ well-being with PROMIS Short Forms. The team will convert raw scores to T-scores and report descriptive statistics (mean ± SD) for each of the five domains (i.e., emotional support, meaning and purpose, anger, anxiety, and depression) separately for each treatment group. To compare each domain composite scores between the ESC care approach and usual care, the team will use a GLMM model with identity link with a fixed effect for the intervention group, time, and group-by-time and a random effect for study site. Assessment periods will include at discharge, and at 6 months and 24 months. The team will report point estimates for the group mean difference along with a 95% CI. Again, this analytical approach will be repeated for each of the 5 PROMIS domains.

##### Maternal postnatal attachment questionnaire

The analysis team will examine the impact of the ESC care approach on the composite score of the MPAQ and its three subscales (quality, absence of hostility towards infant, and pleasure). Since these measures are continuous, the team will apply the GLMM with the normal link function. In addition, the model will examine the ESC intervention impact at hospital discharge and 6 months of age.

##### Family environment scale (FES) at 3 months

Initially, we will base the overall assessment of the FES using the relationship dimension on a composite score of the 30 true-false items found on form R (i.e., range of 0-30). The analysis team will provide the mean and SD of the composite FES scores separately for each treatment group, and the team will use a GLMM with an identity link function to compare average FES scores between the ESC care approach and usual care. We will report point estimates for the group mean difference along with a 95% CI. The team will use the same four model building sequence described for the primary outcome. Additionally, we will repeat the analytical for each relationship dimension, namely, Cohesion, Expressiveness, and Conflict subscales.

##### Parenting sense of competence scale (PSOC)

The analysis team will report descriptive statistics (mean ± SD) for the composite PSOC score separately for each treatment group. The team will compare the PSOC composite scores assessed at hospital discharge and 6-months of age using a GLMM model with identity link with a fixed effect for the intervention group, time, and group-by-time and a random effect for study site. We will calculate the point estimates and their respective CIs for the changes in PSOC scores for each intervention group and for the difference in the estimated change between intervention groups. Additionally, the team will present the p-value of the difference in point estimates between intervention groups.

##### Infant behavior questionnaire (IBQ-R) revised very short form at 3 and 12 months of age

The analysis team will report descriptive statistics (mean ± SD) for each domain of the IBQ-R (i.e., positive affectivity/surgency, negative emotionality, and orienting/regulatory capacity) separately for each treatment group. The team will compare the IBQ-R composite scores for each domain using separate GLMM models with identity link with a fixed effect for the intervention group, time, and group-by-time and a random effect for study site. The team will report point estimates for the group mean difference along with a 95% CI for each domain.

##### Bayley scales of infant and toddler development, fourth edition (Bayley-4): cognitive, language and motor at 24-months of age

The analysis team will calculate descriptive statistics (mean ± SD, medians, percentiles) for each domain in the Bayley-4 separately for each treatment group. To compare the scores between the ESC and usual care groups, we will perform a linear mixed-effects model with a fixed effect for the intervention group and a random effect for study site. We will report point estimates for the group mean difference along with a 95% CI, and the team will repeat this analytical approach for each of the Bayley-4 domains.

##### Brief infant-toddler social and emotional assessment (BITSEA) at 24-months of age

The analysis team will calculate descriptive statistics (mean ± SD, medians, percentiles) for BITSEA problem scale and BITSEA competence scale separately for each treatment group. To compare the scores between the ESC and usual care groups among the two BITSEA scales, we will perform separate linear mixed-effects model with a fixed effect for the intervention group and a random effect for study site. We will report point estimates for the group mean difference along with a 95% CI.

##### Influence of maternal childhood experiences on infant outcomes

The analysis team will calculate descriptive statistics (mean ± SD, medians, percentiles) for the ACE Questionnaire separately for each treatment group. To examine the relationship between the ACE Questionnaire with the IBQ-R scores and Bayley-4, the analysis team will compute separate marginal Pearson correlation coefficients, [62] which is an analog of the standard Pearson correlation coefficient for clustered data. If significant, we will perform a sensitivity analysis in which we include the ACE Questionnaire scores as a covariate in the final analytic models for Bayley-4 and IBQ-R scores.

### Interim analysis

In a stepped-wedge randomized controlled trial, interim analyses for outcomes carried out early in the trial will have a large imbalance between numbers of observations exposed to usual care and intervention conditions. The imbalance will likely have power implications and will make a power analysis infeasible. The clustered natures of the data will also impact the analysis [63, 64]. Therefore, the protocol study team will not conduct an interim analysis on the primary outcome for the purpose of study termination due to inferiority or superiority of the ESC care approach. The protocol study team will conduct an interim analysis for the long-term follow-up portion of the study to assess for futility due to under-recruitment. The projected informed consent rate for long-term follow-up is 30-40%. After each block of two periods (approximately 6 months), the protocol study team will compare the informed consent rate with the projected informed consent rate. If the actual informed consent rate over a block of two periods is below 30%, then the protocol study team will monitor the informed consent rate for another block of two periods. If the cumulative informed consent rate remains below 30%, then the protocol study team will ask the Data and Safety Monitoring Committee (DSMC) to review accrual trajectories and to determine, with the protocol study team, if measures can be taken to improve the accrual rate. The DSMC will consider whether to stop accrual to the long-term follow-up portion of the study due to an insufficient informed consent rate. Additionally, the DSMC will monitor the study for safety concerns.

### Sample size and power estimates

We based the sample size estimate (Table 5) on the primary outcome, which is the comparison of the average length of time until the infant is medically ready for discharge between groups (ESC care approach versus usual care). In much of the literature, researchers tend to report overall length of inpatient hospital stay (LOS). The average reported LOS for infants managed for NOWS is approximately 18 days (SD=8) [28]; we expect a reduction of 4 days with use of the ESC care approach. For this study, we used preliminary data from the ACT NOW Current Experience Study to obtain the mean and standard deviation estimate for LOS. For the purpose of our sample size justification, we used these estimates as a proxy for our estimates of the average length of time until the infant is medically ready for discharge. Based on the Current Experience Study, the average LOS is approximately 11 days (SD=11). Additionally, we derived an estimate of the ICC of 0.25 from this preliminary data analysis. Richard Hooper and colleagues [65] noted that most sample size justifications for stepped-wedge design studies follow a mixed-effects regression approach for cross-sectional stepped-wedge design, as described by Hussey and Hughes, [66] which assumes that the within-period ICC and between-period ICC are equal. They define the cluster autocorrelation coefficient (CAC) as the ratio of the between-period ICC over the within-period ICC.

**Table 5:** Sample size estimates

		3 days		3.5 days		4 days	
Power	CAC	Cluster Size	N	Cluster Size	N	Cluster Size	N
90%	0.8	10	2160	6	1296	4	864
85%	0.8	8	1728	5	1080	3	648
80%	0.8	6	1296	4	864	3	648
90%	0.7	14	3024	7	1512	5	1080
85%	0.7	10	2160	6	1296	4	864
80%	0.7	8	1728	5	1080	3	648
90%	0.6	26	5616	9	1944	5	1080
85%	0.6	14	3024	6	1296	4	864
80%	0.6	10	2160	5	1080	3	648

*CAC* cluster autocorrelation coefficient

We calculated statistical power based on the methodology for stepped-wedge with transition period design proposed by Hooper et al using the R-Shiny app written by Hemming and Kasza [65]. Given that our primary outcome is a count measure, we used the ACT NOW Current Experience Study to obtain an estimate of the over-dispersion parameter (φ). McCullagh and Nelder suggested that the over-dispersion parameter estimate (φ) is simply a ratio of the deviance or the Pearson chi-square to its associated degrees-of-freedom [67]. Thus, a total sample size of 864 infants would achieve 90% power to detect a difference of 4 days between the groups with an estimated CAC of 0.8 and φ=10. This assumes an 8-step stepped-wedge with transition period design with approximately 24 total sites. We will randomize each site into 1 of 8 blocks, and we expect each site to enroll an average of 4 infants during each period for 36 total infants per site during the study duration. Since we have no prior information regarding the CAC estimate, Table 5 provides the total sample size required assuming a CAC ranging between 0.6 to 0.8 and differences of 3 days, 3.5 days, and 4 days. Based on the ACT NOW Current Experience Study, the expected number of infants with NOWS delivered at participating sites annually will be approximately 1500-2000 infants. Therefore, our study will still be sufficiently powered (i.e., 85%) to detect a difference of 3 days between the groups with CAC=0.8. The power calculation assumes significance level of 5%, delivery of infants with NOWS equally distributed across hospital groupings, and analysis by Negative Binomial GLMM.

To address the primary study hypothesis, the protocol study team will randomize a minimum of 24 sites, and a maximum of 28 sites to 1 of 8 blocks of a stepped wedge with transition design (Table 1), with each site enrolling an average of 36 infants. During any single study period (see Table 1), a site may enroll no more than 16 infants. Although we calculated the sample size for the overall trial using the power calculation for the primary hypothesis, we conducted the following power calculations to assure adequacy of sample size to show potential effect of the intervention on infant neurobehavioral functioning and development. To evaluate the impact of the ESC care approach on infant neurobehavioral function and development using measures such as the IBQ-R and Bayley-4, we must obtain primary caregiver consent. We anticipate that not all participants will provide consent for the long-term outcome portion of the study. Table 6 provides an estimate of the effect size based on varying consent rates and CAC estimates with the study having 80% power. Again based on the ACT NOW Current Experience Study, we expect the number of infants with NOWS delivered at participating sites annually will be approximately 1500 infants. Thus, for a 17-month enrollment period, the protocol study team expects a total of 2125 infants with NOWS will be delivered at participating study sites. Assuming a 40% or 30% consent rate, this produces a total sample size of 850 (40% consent rate) or 638 (30% consent rate) infants. Cohen defined effect size as the mean differences, *μ*_1_ − *μ*_2_, divided by the standard deviation, σ, of either group [68]. However, Rosnow and Rosenthal noted that in practice, researchers commonly use the pooled SD (defined as the root mean square of the 2 SDs) [69]. Effect sizes are generally classified as small (≤ 0.3), medium (~0.5), and large (≥ 0.75). For infant neurobehavioral functioning based on the IBQ-R, the study will have 80% power to detect an expected mean difference of 0.28 points in the Orienting/Regulatory Capacity domain, assuming a 30% consent rate and CAC=0.8, based on a mixed-effects model with a fixed treatment effect and random site effect with a significance level of 0.05. With a SD of 0.70, the detectable mean difference constitutes a moderate effect size. We based our estimated mean (5.0) and SD (0.70) for the Orienting/Regulatory Capacity domain using the summary statistics provided by Putnam and colleagues, [45] in which the authors provided summary data of the IBQ-R domains extracted from six standard form data samples.

**Table 6:** Sample size estimates for the IBQ-R for the consented subpopulation

Consent Rate	Total Sample Size	Effect Size (Δ)	Mean Difference	CAC	Power
40%	850	0.35	0.25	0.8	0.80
40%	850	0.38	0.27	0.7	0.80
40%	850	0.40	0.28	0.6	0.80
30%	638	0.40	0.28	0.8	0.80
30%	638	0.43	0.30	0.7	0.80
30%	638	0.45	0.32	0.6	0.80

*CAC* cluster autocorrelation coefficient

For the neurodevelopmental outcome based on the Bayley-4, the study will have 80% power to detect an expected mean difference of 6 points, assuming a 30% consent rate and CAC=0.8, based on a mixed-effects model with a fixed treatment effect and random site effect with a significance level of 0.05. With a SD of 15, the detectable mean difference constitutes a moderate effect size.

### Available population

In December 2018, we completed data abstraction for the ACT NOW Current Experience Study. Twenty-five ISPCTN and 5 NRN sites participated in this study. We collected data for 1808 infants with opioid exposure across these networks. Of these infants, approximately 40% were treated pharmacologically during the 1-year target period of July 1, 2016, to June 30, 2017 (86% morphine, 13% methadone, <1% buprenorphine).

### Projected recruitment time

#### Site recruitment

We will recruit approximately 24 sites for this study. We will randomize these sites into 8 blocks. Initial assessment of site interest in study participation across the networks suggests an adequate number of sites to meet our site recruitment goal. The site’s ability to initiate a change in practice within their organization will impact actual site recruitment. Recruitment of all sites will take an estimated 3 months. We will randomize sites into blocks once recruitment is complete.

#### Site training and implementation

Site training and implementation will take approximately 3 months, as we will first train a core group of site champions, followed by training of all site personnel by the core group. The protocol study team will train the site champions off-site and as such, training may occur in parallel with the end of the final usual care period at the site. Once a site has achieved the training milestones, the site will formally implement ESC. After this initial implementation, the site will step into the ESC period (see Table 1). Total enrollment period is 20 months with each site actively enrolling infants for 17 of the 20 months. If the site research team obtains consent for the long-term follow-up portion of the trial, the site research team will follow the infant for 24 months. Total length of the study will be approximately 44 months.

## Study monitoring plan

We will conduct clinical site monitoring to ensure that we protect the rights and well-being of study participants, that the reported trial data are accurate, complete, and verifiable, and that the conduct of the study complies with the currently approved protocol/amendment(s), with International Council for Harmonisation Good Clinical Practice, and with applicable regulatory requirements.

- A member of the DCC clinical operations staff or their designee will monitor the study.
- The clinical monitoring team will plan and conduct an on-site visit at least once during the course of the study and more often if needed for cause.
- Details of clinical site monitoring are in the Clinical Monitoring Plan. The plan describes who will conduct the monitoring, at what frequency monitoring will occur, at what level of detail monitoring will be performed, and how monitoring reports will be distributed.

### Adverse events

#### Definition of adverse events and serious adverse events

Adverse event (AE): AE means any untoward medical occurrence associated with the use of an intervention in humans, whether or not considered intervention related.

Serious Adverse Event (SAE): An AE is considered "serious" if, in the view of either the investigator or sponsor, it results in any of the following outcomes:

1. Death
2. Life-threatening AE (life-threatening means that the study participant was, in the opinion of the investigator or sponsor, at immediate risk of death from the reaction as it occurred and required intervention)
3. Persistent or significant incapacity or substantial disruption of the ability to conduct normal life functions
4. Inpatient hospitalization or prolongation of existing hospitalization
5. Important medical event that may not result in 1 of the above outcomes but may jeopardize the health of the study participant or require medical or surgical intervention to prevent 1 of the outcomes listed in the above definition of serious event

#### Classification of an adverse event

##### Severity of event

For AEs, the site research team will use the following guidelines to describe severity. The site investigator will determine severity.

- **Mild** – Events require minimal or no treatment and do not interfere with the participant’s daily activities.
- **Moderate** – Events result in a low level of inconvenience or concern with the therapeutic measures. Moderate events may cause some interference with functioning.
- **Severe** – Events interrupt a participant’s usual daily activity and may require systemic drug therapy or other treatment. Severe events are usually potentially life threatening or incapacitating. Of note, the term “severe” does not necessarily equate to “serious.”

##### Relationship to study intervention

The site research team will grade the degree of certainty about causality by using the categories below.

- **Related** – The AE is known to occur with the study procedures, there is a reasonable possibility that the study procedures caused the AE, or there is a temporal relationship between the study procedures and the event. Reasonable possibility means that there is evidence to suggest a causal relationship between the study procedures and the AE.
- **Not Related** – There is not a reasonable possibility that the study procedures caused the event, there is no temporal relationship between the study procedures and event onset, or an alternate etiology has been established.

##### Expected AEs

Expected AEs include - seizures, accidental trauma, severe weight loss (greater than 15% from birthweight) and respiratory insufficiency. Expected AEs that could occur during the follow-up portion of the study include acute/urgent care and or ER visits for worsening symptoms of NOWS. Hospital readmission to assess and manage symptoms of NOWS and non-accidental trauma may also occur. We note anticipated rates in Table 7.

**Table 7:** Expected rates of safety outcomes

Safety Outcomes	Expected Rate
Inpatient Safety Composite	
Seizures [70, 71]	1%
Accidental trauma (i.e., dropped infants) [72, 73]	4 falls per 10,000 births
Respiratory insufficiency due to opioid therapy	0.5%
Outpatient Safety Composite	
Acute/urgent care and/or ER visits – 1 visit in first 6 months of life [74]	35%
Hospital Readmissions in the first 6 months of life [74]	7%

#### Time period and frequency for event assessment and follow up

For this study, the protocol study team will collect the following AEs: 1) all expected AEs (seizures, accidental trauma, severe weight loss, and respiratory insufficiency), and 2) SAEs related to study procedures. The occurrence of an AE or SAE may come to the attention of study personnel during the hospital stay, by the clinical team with administration of questionnaires, or by the medical monitor upon reviewing data. The site research team will capture all AEs on the appropriate case report form. Information to be collected includes event description, date/time of onset, date/time of resolution, clinician’s assessment of severity, relationship to study intervention and time of resolution/stabilization of the event. Site research teams must follow all AEs until the AE meets one of the following criteria: resolution, the condition stabilizes, the event is otherwise explained or is judged by the protocol study team to be no longer clinically significant, or the participant is lost to follow-up. The site research team will collect AEs during the initial hospitalization through hospital discharge.

### Data monitoring and safety

The independent DSMC will have overall responsibility for interim data monitoring and operate based on the ISPCTN and NRN charter for the DSMC. The DSMC will formally review interim safety data in a sequential fashion using interim monitoring boundaries after approximately 25%, 50%, and 75% of the study sites (6, 12, and 18 sites, respectively) have transitioned to ESC. Treatment groups will be compared statistically using the analysis planned for the final analyses for safety outcomes as previously outlined.

Safety oversight will be under the direction of a DSMC. Safety outcomes include the components of the inpatient composite safety outcome and those of the outpatient composite safety outcome (see Table 7). The DSMC may request other outcomes at their discretion. Formal statistical testing for an imbalance of seizures, accidental trauma, or respiratory insufficiency due to opioid therapy, in either treatment group, will be based on a comparatively liberal Lan DeMets Pocock boundary at the three interim safety reviews to guard against any occurrence of false positives while at the same time allowing for stopping at reasonable levels of evidence. Thus, at each interim, an increased incidence of seizures in either treatment group with P < 0.0179 (for 4 total tests) will be considered a statistically significant evidence of harm that the DSMC can use to recommend suspension of the trial for safety reasons. This same statistical testing will also be conducted for the components of the outpatient composite safety outcome. In addition to the formal safety outcomes, the DSMC will examine other safety outcomes, including all reported SAEs by treatment group in considering a recommendation to suspend the trial for safety reasons.

The Medical Monitor will provide input on safety considerations, evaluate safety trends, and provide oversight throughout the life cycle of the clinical research, in accordance with the approved protocol. This role includes reviewing and monitoring safety events on a regular basis, advising the protocol investigators on trial-related medical questions or problems, reviewing cumulative participant safety data, and making recommendations regarding the data to the DSMC.

## Data management

The data management center, RTI International, will:

- Collaborate in the development, implementation, and monitoring of ESC protocol.
- Provide data management, including development of CRFs and appropriate data collection systems
- Supervise data entry activities, including instructing and certifying data entry personnel in software and hardware usage, quality assurance of data entry, etc.
- Manage the Data Safety and Monitoring Committee for the trial. This will include scheduling meetings and the DSMC charter.
- Oversee the receipt and reconciliation of safety data.
- Supervise NRN-site quality assurance efforts, including conducting site visits and remote monitoring of data.
- Prepare and distributes monthly reports, detailing data received, data consistency, missing data and adherence to protocol.
- Disburse capitation payments to clinical centers on the basis of enrolled participants and other study-specific milestone triggers specified in the study protocol.
- Provide the logistical support necessary to run an efficient and productive network.

## Publication and data sharing policy

This study will comply with the National Institutes of Health (NIH) Public Access Policy, which ensures that the public has access to the published results of NIH-funded research. The study will also comply with the NIH Data Sharing Policy and Policy on the Dissemination of NIH-Funded Clinical Trial Information and the Clinical Trials Registration and Results Information Submission rule.

As such, this study will:

- Register with ClinicalTrials.gov and submit primary outcome results. The ClinicalTrial.gov number is NCT04057820.
- Publish results. The protocol study team will make every attempt to publish results in peer-reviewed journals. The team will submit all final peer-reviewed journal manuscripts from this study to the digital archive PubMed Central upon acceptance for publication.
- Deposit data for data sharing with other researchers. Within the bounds of relevant IRB approvals and guidelines for protection of personally identifiable data, the protocol study team will deposit de-identified data from this study in an appropriate, NIH-approved data repository.

## Trial status

Protocol version 5, August 7, 2020

Enrollment began on September 8, 2020. Anticipated end of enrollment is March 22, 2022. Long-term follow up will be complete May 2024.

Trial registration: NCT04057820; registered August 15, 2019

## Supplementary Information

**Additional file 1.**

**Additional file 2.**

**Additional file 3.**

**Additional file 4.**

## Data Availability

This study will comply with the NIH Data Sharing Policy and Policy on the Dissemination of NIH-Funded Clinical Trial Information. Non-proprietary case report forms will be available on the NICHD Data and Specimen Hub.

## Abbreviations

ACE: Adverse Childhood Experience
AE: Adverse Event
Bayley-4: Bayley Scales of Infant and Toddler Development, 4^th^ Edition
BISQ: Brief Infant Sleep Questionnaire
BITSEA: Brief Infant-Toddler Social and Emotional Assessment
BMI: Body Mass Index
CAC: Cluster Autocorrelation Coefficient
CI: Confidence Interval
COVID-19: Severe acute respiratory syndrome coronavirus-2
Cox PH: Cox Proportional Hazards
CQ: Caregiver Questionnaire
DCC: Data Coordinating Center
DSMC: Data and Safety Monitoring Committee
EDC: Electronic Data Capture
EHR: Electronic Health Record
ER: Emergency Room
ESC: Eating, Sleeping, Consoling
FCOI: Financial Conflict of Interest
FES: Family Environmental Scale
FNAST: Finnegan Neonatal Abstinence Scoring Tool
GLMM: Generalized Linear Mixed Model
GSI: Global Severity Index
HIPAA: Health Insurance Portability and Accountability Act
IBQ: Infant Behavior Questionnaire
IBQ-R: Infant Behavior Questionnaire – Revised
ICC: Intracluster Correlation Coefficient
IRB: Institutional Review Board
IRR: Inter-Rater Reliability
ISPCTN: IDeA States Pediatric Clinical Trials Network
LOS: Length of Stay
MOP: Manual of Procedures
MPAQ: Maternal Postnatal Attachment Questionnaire
NIH: National Institutes of Health
NRN: Neonatal Research Network
NOWS: Neonatal Opioid Withdrawal Syndrome
PI: Principal Investigator
PROMIS: Patient Reported Outcome Measurement Information System
PSOC: Parenting Sense of Competence
QC: Quality Control
QI: Quality Improvement
SAE: Serious Adverse Event
SD: Standard Deviation
WHO: World Health Organization
