# Supplementary material for: Eating, Sleeping, Consoling for Neonatal Opioid Withdrawal (ESC-NOW): a Function-Based Assessment and Management Approach study protocol for a multi-center, stepped-wedge randomized controlled trial
Source: Trials. 2022 Aug 9;23:638. doi: 10.1186/s13063-022-06445-z (PMC9361241; doi:10.1186/s13063-022-06445-z)
Supplement: Supplementary file 1 — Additional file 1. [file 13063_2022_6445_MOESM1_ESM.docx]

**Roles and Responsibilities**

Members of the Steering Committee include the lead study investigators, primary investigators from each of the coordinating centers and their designated teams, a scientific officer from the NIH, and senior researcher consultants with extensive experience in clinical trials.

**Lead Study Investigators**

The lead study investigators will be responsible for protocol development, conduct, and maintenance of scientific integrity. Lead study investigators will also be responsible for the development of the scientific publications and presentations for the primary study results.

1. *Leslie W. Young, MD*
2. *Lori Devlin, DO, MHA, MS*
3. *Stephanie Merhar, MD, MS*

**Coordinating Centers**

Three coordinating centers, referred to collectively as the DCC will collaborate  to perform all coordination activities for the ACT NOW collaborative. The coordinating centers consist of:

1. ECHO Coordinating Center – Duke Clinical Research Institute (DCRI)
2. NRN Data Coordinating Center – RTI International
3. ECHO ISPCTN Data Coordinating and Operations Center – University of Arkansas for Medical Sciences (UAMS)

The DCCs will be jointly responsible for:

1. Collaborating in the development, implementation, and monitoring of ACT NOW protocols.
2. Assuring all clinical centers have required regulatory materials, human subjects, and NIH Good Clinical Practice (GCP) trainings.
3. Communication of relevant study matters (ie. protocol modifications, participant enrollment, etc.) to the appropriate and respective parties.
4. Disbursing capitation payments to clinical centers/sites on the basis of enrolled participants and other study-specific milestone triggers specified in the approved study protocols and budgets (each DCC is responsible for doing this via its Network’s existing infrastructure mechanisms, policies, and procedures).
5. For clinical sites that are not in either network, the DCCs will decide which DCC will be responsible for disbursing capitation payments to each clinical center/site.

Duke Clinical Research Institute (DCRI**)**

*PI: Rachel G. Greenberg, MD, MB, MHS*

In support of this study, DCRI will:

1.    Be responsible for the daily operations across all ISPCTN and non-network sites.

2.    Monitor the performance of ISPCTN/non-network clinical sites and meet with NICHD and NIH on   a regular basis to review site performance.

3.    Administer site contracts with ISPCTN and non-network sites.

4.    Pay site capitation to ISPCTN and non-network sites.

RTI International

*PI: Abhik Das, PhD*

In support of this trial, RTI will:

1.    Be responsible for the daily operations across all NRN sites.

2.    Develop and maintain case report forms (CRFs) and the study Electronic Data Capture (EDC) systems.

3.    Ensure the quality and accuracy of data and adherence to study protocol for all Clinical Sites.

4.    Monitor the performance of NRN Clinical Sites and meet with NICHD and NIH on a regular basis  to review site performance.

5.    Monitor safety surveillance and facilitate the expedited adverse event reporting process to the DSMC and NICHD/NIH as needed for all Clinical Sites

- RTI will notifying the DSMC chairperson or vice chairperson of the DSMC, as needed and as specified in the protocol, of any unexpected, serious, and protocol-related adverse events or apparent trends in data possibly relevant to patient safety
- At periodic intervals, as specified in the protocols, (and in coordination with the ISPCTN-ECHO/OD DCC for its assigned protocols), the NRN DCC provides the DSMC with confidential interim analyses of study data related to protocol performance, patient safety, and emerging results related to efficacy and futility

6.    Prepare data and reports for the trial.

7.    Pay site capitation to NRN sites.

University of Arkansas for Medical Sciences (UAMS)

*PI: Jessica Snowden, MD*

*Lead Statistician: Songthip Ounpraseuth, PhD*

In support of this study, UAMS will:

1.    Act as the central IRB for this protocol.

2.    Be responsible for protocol training, intervention specific (ESC) training and maintenance of fidelity (inter-rater reliability and process implementation) for NRN, ISPCTN, and non-network sites.

3.    Be responsible for randomization and statistical analysis.

**NIH Scientific Officer**

*Alan Simon, MD*

*Michele Walsh, MD*

**Senior Research Consultants**

*P. Brian Smith, MD*

*Jeannette Y. Lee, PhD*

*Brenda Poindexter, MD, MS*

**DSMC**

1. The Data and Safety Monitoring Committee (DSMC) will monitor the safety and data integrity.
2. The DSMC is responsible for safeguarding the interests and assessing the safety of study participants.
3. The DSMC will advise NICHD and the ECHO/OD program office on research design, data quality, and analysis issues pertaining to interim monitoring for safety and efficacy, and ethical and human subject aspects of studies.
4. The DSMC will review and approve the protocol and consent form. f
5. NICHD will assume responsibility for administration of the DSMC.
6. The ACT NOW trials will use the existing NRN DSMC under its current existing charter and add the ECHO/OD Program Officer as a non-voting observer, and other specialists as needed, for the ACT NOW study reviews. For the ACT NOW trials, the DSMC will report both to the Director of NICHD and the Director of the ECHO/OD program.

**Study Team Composition**

| **Lead Study Investigator** | |
| --- | --- |
| *Name, degree, title* | Leslie W. Young, MD, FAAP  Associate Professor, Pediatrics  Division of Neonatal-Perinatal Medicine |
| *Institution* | *Department of Pediatrics, Larner College of Medicine at the University of Vermont* |
| *Address* | *111 Colchester Ave Smith 5 Burlington, VT 05401* |
| *Email* | [Leslie.Young@uvmhealth.org](file:///\\uvmmc\Users\loriphinney\Downloads\Leslie.Young@uvmhealth.org) |
| **Lead Study Investigator** | |
| *Name, degree, title* | Lori Devlin, DO, MHA, MS, FAAP  Associate Professor, Pediatrics  Division of Neonatal Perinatal Medicine |
| *Institution* | *Department of Pediatrics, University of Louisville School of Medicine* |
| *Address* | *571 South Floyd Street Suite 342, Louisville, KY 40202* |
| *Email* | [Lori.Devlinphinney@louisville.edu](mailto:Lori.Devlinphinney@louisville.edu) |
| **Lead Study Investigator** | |
| *Name, degree, title* | Stephanie Merhar, MD, MS  Associate Professor, Pediatrics  Division of Neonatal Perinatal Medicine |
| *Institution* | *Perinatal Institute, Division of Neonatology, Cincinnati Children's Hospital Medical Center, Cincinnati, Ohio, Department of Pediatrics, University of Cincinnati, Cincinnati OH* |
| *Address* | *3333 Burnet Ave ML7009 Cincinnati, OH 45229* |
| *Email* | [Stephanie.Merhar@cchmc.org](file:///\\uvmmc\Users\loriphinney\Downloads\Stephanie.Merhar@cchmc.org) |
| **Statistician** | |
| *Name, degree, title* | Songthip Ounpraseuth, PhD  Associate Professor, Biostatistics |
| *Institution* | *University of Arkansas for Medical Sciences* |
| *Address* | *4301 W. Markham St., Little Rock, AR 72205* |
| *Email* | [stounpraseuth@uams.edu](file:///\\uvmmc\Users\loriphinney\Downloads\stounpraseuth@uams.edu) |
| **Duke Coordinating Center (DCC) PI** | |
| *Name, degree, title* | Rachel G. Greenberg, MD, MB, MHS  Associate Professor of Pediatrics  Division of Neonatal Perinatal Medicine |
| *Institution* | *Duke University School of Medicine Duke Clinical Research Institute* |
| *Address* | *300 W. Morgan St Durham, NC 27701* |
| *Email* | [rachel.greenberg@duke.edu](file:///\\uvmmc\Users\loriphinney\Downloads\rachel.greenberg@duke.edu) |
| **RTI Coordinating Center (DCC) PI** | |
| Name, degree, title | Abhik Das, PhD Distinguished Fellow, Biostatistics |
| Institution | *RTI International* |
| *Address* | *3040 East Cornwallis Road*  *Research Triangle Park, NC 27709-2194* |
| *Email* | [adas@rti.org](file:///\\uvmmc\Users\loriphinney\Downloads\adas@rti.org) |
| **Data Coordinating and Operations Center (DCOC) PI** | |
| *Name, degree, title* | Jessica Snowden, MD  Associate Professor, Pediatric  Division of Pediatric Infectious Diseases |
| *Institution* | *Arkansas Children’s Research Institute /*  *University of Arkansas for Medical Sciences* |
| *Address* | *13 Children’s Way, ACRI Slot 512-35, Little Rock, AR 72202* |
| *Email* | [jsnowden@uams.edu](mailto:jsnowden@uams.edu) |
| **Subcommittee Member** | |
| *Name, degree, title* | Jeannette Y. Lee, Ph.D.  Professor, Biostatistics |
| *Institution* | *University of Arkansas for Medical Sciences* |
| *Address* | *4301 West Markham, #781*  *COPH Room 3234*  *Little Rock, Arkansas 72205-7199* |
| *Email* | [jylee@uams.edu](http://jylee@uams.edu) |
| **Subcommittee Member** | |
| *Name, degree, title* | Brian Smith MD MPH MHS  Professor, Pediatrics  Division of Neonatal-Perinatal Medicine |
| *Institution* | *Duke University Medical Center*  *Duke Clinical Research Institute* |
| *Address* | *300 W. Morgan St Durham, NC 27701* |
| *Email* | [brian.smith@duke.edu](mailto:brian.smith@duke.edu) |
| **Subcommittee Member** | |
| *Name, degree, title* | Brenda Poindexter, MD, MS  Professor, Pediatrics  Division of Neonatal-Perinatal Medicine |
| *Institution* | *Children’s Healthcare of Atlanta and Emory University* |
| *Address* | *2015 Uppergate Dr. NE, Suite 304 Atlanta, GA 30322* |
| *Email* | [brenda.poindexter@emory.edu](mailto:brenda.poindexter@emory.edu) |

**Clinical Sites**

Participating clinical sites will be selected from the NRN network, the ECHO ISPCTN network and from outside of these two networks. Clinical Sites will be responsible for the following:

- Implementing and following the study protocol
- Participating in all relevant trainings
- Conducting the intervention(s) and ensuring the fidelity of the intervention(s) at their sites
- Ensuring that the site research team at the site are up-to-date on all relevant training and regulatory requirements
- Obtaining informed consent and tracking participants for long-term follow-up
- Collecting data during intervention and long-term follow-up period
- Transferring the data to the Data Coordinating Center (DCC)
- Participating in all site monitoring activities

List of Participating Sites*:

| **Name** | **Location** | **PI**** |
| --- | --- | --- |
| Shawnee Mission Medical Center | Shawnee Mission, Kansas | Julie Weiner |
| University of Mississippi Medical Center | Jackson, MS | Lauren Tucker |
| University of Nebraska Medical Center | Omaha, NE | Ann Anderson-Berry |
| Christiana Care Health Systems | Newark, DE | David Paul |
| Duke University | Durham, NC | Sophie Shaikh |
| Sanford Health | Sioux Falls, SD | Michelle Baack |
| University of Utah Medical Center | Salt Lake City, UT | Camille Fung |
| Norton Children’s Hospital | Louisville, KY | Sucheta Telang |
| Medical University of South Carolina | Charleston, SC | Julie Ross |
| Good Samaritan Hospital | Cincinnati, OH | Stephanie Merhar |
| University of Buffalo | Buffalo, NY | Anne Marie Reynolds |
| St. Elizabeth Healthcare/CCHMC | Edgewood, KY | Ward Rice |
| University of Kansas Medical Center | Kansas City, KS | Krishna Dummula |
| Oklahoma University Health Sciences Center | Oklahoma City, OK | Devon Hahn |
| University of South Florida | Tampa, FL | Maya Balakrishnan |
| Hospital of the University of Pennsylvania (HUP) | Philadelphia, PA | Lori Christ |
| University of Cincinnati (University Hospital) | Cincinnati, OH | Kurt Schibler |
| Kapiolani Hospital | Honolulu, HI | Charles Neal |
| Spartanburg Regional Medical Center | Spartanburg, SC | Jaime Brown |
| Lahey Health | Winchester, MA | Karen McAlmon |
| Nationwide Children's Hospital | Columbus, OH | Erica Braswell |
| University of New Mexico, Health Sciences Center | Albuquerque, NM | Jessie Maxwell |
| Tulane University School of Medicine | New Orleans, LA | Meghan Howell |
| Case Western Reserve University (University Hospitals Health System) | Cleveland, OH | Moira Crowley |
| Pennsylvania Hospital | Philadelphia, PA | Karen Puopolo |
| University of Rochester | Rochester, NY | Julie Riccio |

*All participating sites are either academic sites or community sites affiliated with a university health system.

**Declaration of interests; all site PIs have reported no conflict of interest.

**Data Management**

The ACT NOW ESC study uses Medidata Rave®, developed by Medidata Solutions, Inc., for web-based electronic clinical data capture (EDC) and clinical data management.  Data managed uses the secure and reliable facilities of Medidata Solutions hosting services. The Medidata Rave application follows good clinical practice (GCP) guidelines and is compliant with FDA Regulation 21 CFR Part 11, ICH E6 sections on data handling and record keeping, and globally, with EMEA and MHLW requirements. Medidata’s operating system has been evaluated for compliance with FIPS 140-2 encryption by NIST. Additionally, hosting sites are monitored 24x7 by on-site security personnel, and no areas are publicly accessible. At the application level, access requires correct entry of username and password by sponsor-authorized users only. Role-based security permissions are utilized within the system to limit views and actions of authorized users.

Additionally, caregiver surveys are collected via the Research Electronic Data Capture (REDCap) system.  This system was developed by Vanderbilt University and the instance that will be used for the ACT NOW project is installed on RTI’s cloud based web servers and is administered by RTI clinical research informatics programming staff.  The REDCap EDC follows good clinical practice (GCP) guidelines. Hosting servers are ISO/IEC 27001:2013 certified as well as comply with the EU-U.S. and Swiss-U.S. Privacy Shield Frameworks and is physically housed in the U.S.  Similar to Medidata Rave, user access requires correct entry of username and password by sponsor-authorized users or via a weblink provided by authorized system users to caregivers that will provide access to an individual survey and nothing else.  Role-based security permission will limit views and actions to ensure users only perform authorized tasks.

Appropriate study data will be keyed into the eCRFs of the Rave and REDCap EDC system by a designated member of the site study team or the caregiver, if REDCap, ideally within 1 week of data capture on the paper CRF. Data entry will be single entry performed at the research site locations. Data accuracy will be validated using programmed EDC edit checks and manual EDC queries.  Data cleaning in Rave and REDCap will be conducted on a regular basis to ensure the conduct of the trial follows the currently approved protocol and GCP. Programmed system edit checks, manual edit checks, and ad hoc reports will be generated to aid in the data cleaning process.

To assure the quality of the data collected, the protocol study team will provide training specific to accuracy of data acquisition for the research coordinators at each site. The protocol study team will design data collection forms, which a subset of sites will subsequently pilot to minimize the potential for errors. Additionally, the protocol study team will allocate sufficient funds to allow for quality data collection. The site research team will re-abstract a subsample of their own charts and assess the error rate. Re-abstraction will focus on critical data elements related to the primary and secondary objectives of the protocol. The protocol study team will base the number of charts a site re-abstracts, for each 6-month interval, on the number of patients enrolled in the study during the 6 month period.  The site research team will identify an independent site quality control (QC) abstractor who will re-abstract and enter data into the electronic data capture system (EDC) only for the QC process and will not abstract study data while QC activities are taking place. The DCC will generate a discrepancy report comparing study data abstracted by the site with the source information abstracted by the independent abstractor. The site manager will hold a QC Review Meeting with the independent site QC abstractor, research coordinator, and site abstractor(s) to review the discrepancies and identify errors. Together they will discuss and document the corrective action for each error identified. The DCC will create manual queries in the EDC to make any necessary corrections to the data that QC Review members identify. The protocol study team will provide hospitals that have an error rate above the predefined threshold with additional training, a hospital-specific assessment of the data collection process, and suggestions for process improvement. The protocol study team will track hospitals by their error rates. The protocol study team will share practices of those hospitals with exceptionally low error rates with hospitals working to improve their own process. The protocol study team will review error rates and re-abstraction data during monthly team calls. If errors exceed the predefined threshold on 2 consecutive reviews, a remediation plan will be requested and shared with the study sponsor.

**Confidentiality**

Only Indirect Identifiers (dates and zip codes) will be collected as part of the research data. A subcontract (or DUA) is being developed with each site that include data going to RTI (this agreement will satisfy HIPAA requirements to collect, release and receive limited datasets). The ACT NOW Project has chosen Medidata Rave®, developed by Medidata Solutions, Inc., for web-based electronic clinical data capture (EDC) and clinical data management for these protocols. RTI uses the secure and reliable facilities of Medidata Solutions hosting services. The Medidata Rave application follows good clinical practice (GCP) guidelines and is compliant with FDA Regulation 21 CFR Part 11, ICH E6 sections on data handling and record keeping, and globally, with EMEA and MHLW requirements. Medidata’s operating system has been evaluated for compliance with FIPS 140-2 encryption by NIST. Additionally, hosting sites are monitored 24x7 by on-site security personnel, and no areas are publicly accessible. At the application level, access requires correct entry of username and password by sponsor-authorized users only. Role-based security permissions are utilized within the system to limit views and actions of authorized users. Additionally, there will be a caregiver surveys collected via the Research Electronic Data Capture (REDCap) system. This system was developed by Vanderbilt University and the instance that will be used for the ACT NOW project is installed on RTI’s cloud based web servers and is administered by RTI clinical research informatics programming staff. The REDCap EDC follows good clinical practice (GCP) guidelines. Hosting servers are ISO/IEC 27001:2013 certified as well as comply with the EU-U.S. and Swiss-U.S. Privacy Shield Frameworks and is physically housed in the U.S. Similar to Medidata Rave, user access requires correct entry of username and password by sponsor-authorized users or via a weblink provided by authorized system users to caregivers that will provide access to an individual survey and nothing else. Role-based security permission will limit views and actions to ensure users only perform authorized tasks.

Throughout the study, sites will retain information to contact the participants. After the completion of data collection, records will be retained as long as legally required by local, state, and federal policies and procedures. For the zip codes specifically collected under HIPAA waiver: The ZIP-code information would be retained in the database to link to the Area Health Resources Files from HRSA, RUCA codes from USDA ERS, and data from the US Census Bureau. Once the link is made and the area-wide data attached to a record, the initial ZIP-code would be deleted from future files.
