# Supplementary material for: Eating, Sleeping, Consoling for Neonatal Opioid Withdrawal (ESC-NOW): a Function-Based Assessment and Management Approach study protocol for a multi-center, stepped-wedge randomized controlled trial
Source: Trials. 2022 Aug 9;23:638. doi: 10.1186/s13063-022-06445-z (PMC9361241; doi:10.1186/s13063-022-06445-z)
Supplement: Supplementary file 2 — Additional file 2. [file 13063_2022_6445_MOESM2_ESM.pdf]

Study Title: Eating, Sleeping, Consoling for Neonatal Opioid Withdrawal (ESC-NOW): a Function-Based Assessment and Management Approach
PI (researcher): [insert local context here]
Institution: [insert local context here]
Sponsor: If applicable
Support: NIH

KEY INFORMATION FOR

EATING, SLEEPING, CONSOLING FOR NEONATAL OPIOID WITHDRAWAL (ESC-NOW): A FUNCTION-BASED ASSESSMENT AND MANAGEMENT APPROACH

We are asking you to choose whether or not you and your baby want to be part of a clinical trial (research study) about babies who have neonatal opioid withdrawal syndrome (NOWS). Neonatal opioid withdrawal syndrome (NOWS) can cause a number of problems. These problems may include tremors, seizures, fussiness, vomiting, and poor feeding. Babies can develop NOWS if their mothers took drugs called opioids while the babies were still inside their mothers. There are many different names for opioids. Some brand names, generic names, and street names are listed on the last page of this form.

Your baby received care for NOWS during his/her hospital stay. We are asking to collect information about you/your baby including information about your baby's growth and development. We also want to collect information about your family's environment after your baby leaves the hospital.

This page and the next page give you key information to help you decide whether to be part of this study. We have included detailed information after these key information pages. If you have questions after reading these pages, please ask the research team. If you have questions later, the contact information for the research investigator in charge of the study at your hospital is below.

WHAT IS THE STUDY ABOUT AND HOW LONG WILL IT LAST?

By doing this study, we hope to learn more about how different treatment methods for NOWS affect the growth and development of babies after they have been discharged from the hospital. We also hope to learn more about how these treatment methods may help babies and their caregivers once they are home. You and your baby's participation in this research will take about 6 hours of your time over the course of 2 years.

WHY MIGHT I CHOOSE TO VOLUNTEER FOR THIS STUDY?

You/your baby may not benefit directly from being in this study. Benefits could include one or more of the following:

- Learning more about you and your baby's well-being by responding to questionnaires that you will be asked to answer after discharge from the hospital.
- Learning more about your baby's development by responding to questionnaires that you will be asked to answer after discharge from the hospital.

- For a complete description of benefits, refer to the Full Consent.

WHY MIGHT I CHOOSE NOT TO VOLUNTEER FOR THIS STUDY?

The reason(s) you may not want you/your baby to volunteer for this study include:

- Someone could find out that you were in the study. They could learn something about you/your baby that you did not want others to know. We will do our best to protect your privacy.
- Some of the questions asked may make you feel sad or upset. For a complete description of risks, see the full Consent.

DO I HAVE TO TAKE PART IN THE STUDY?

No. It is okay to say no. If you decide to take part in the study, it should be because you want to volunteer for you and your baby. You/your baby will not lose any services, benefits, or rights you/your baby would normally have if you choose not to volunteer

WHAT IF I HAVE QUESTIONS, SUGGESTIONS, OR CONCERNS?

You can contact the person in charge of the study, [TO BE INSERTED *PI Name and affiliation*, with any questions, suggestions, or concerns at TO BE INSERTED *contact information*.

If you have any questions, suggestions, or concerns about your/your baby's rights as a volunteer in this study, or wish to speak to someone not directly involved in the research, you can call the UAMS Institutional Review Board at 501-686-5667 during business hours. An institutional review board is a group of people who review research to protect the rights and well-being of research participants.

If you want to know more about the research, let the study team know so they can give you more information.<br>Also tell the study team if you have decided you don't want to be in the study. It is perfectly okay to say no.

<Insert local site information> Informed Consent Form

- **We are asking you/your baby to be in a research study. You/your baby do not have to join the study.**
- **You/your baby will still get medical care from [insert local context here] even if you/your baby are not in the study.**
- **Please take as much time as you need to read this form and decide what is right for you/your baby.**

Why am I being asked to be in this clinical trial (research study)?

- Neonatal opioid withdrawal syndrome (NOWS) is something that affects a lot of babies and their families. We are trying to learn as much as we can about the best way to care for babies born with NOWS
- Babies who, while growing inside their mothers, have been exposed to opioids, can have NOWS. There are many names for opioids. Some of the brand names, generic names, and street names are listed on the last page of this form.
- This study may help us learn more about which method(s) might work better than others for treating NOWS. Specifically, the research team is looking to see how different treatment methods for NOWS affect the growth and development of babies after they go home from the hospital. The research team is also looking at how these treatment methods may help babies and their caregivers once they are home.
- We are asking people like you, who have a babies with NOWS, to help us.
- Up to 3,000 babies and their parents or legal guardians will be in this study.
- This research study is sponsored by the National Institutes of Health. It is being conducted at about 24 hospitals across the United States.

What if I don’t understand something?

- This form may have words you don’t understand. If you’d like, research staff will read and explain it with you.
- You are free to ask questions at any time - before, during, or after you/your baby are in the study.

- Please ask as many questions as you like before you decide whether you want you/your baby to be in this study.

What will happen if I say yes, I want to be in this study?

First, we will see if you/your baby qualify to be in the study. We will make sure that all of the following are true:

- Your baby was inside his/her mother for at least 36 weeks.
- Your baby has been managed for NOWS.
- There is either a maternal history of opioid use, or a positive maternal toxicology screen for opioid use, or a positive infant toxicology screen for opioids during your baby's initial hospital stay.

Your baby can not be in this study if he or she has (or had) certain medical problems. The person who is going over this consent with you can list these problems if you want to know what they are.

If you/your baby qualify, we will do these things:

- Ask you to sign this consent form.
- We will assess your baby's and family's well-being using questionnaires. Questionnaires will be done electronically, over the phone, or in person. You do not have to answer any questions you do not want to answer. The description and schedule for these questionnaires are listed in the "Contact Times" below.
- Ask you to bring you baby in so a medical professional can ✓ check your baby's size and weight.
  - ✓ complete an assessment using a series of play-based tests and questions that allow medical professionals to determine how well your child is developing compared to other babies his/her age.
- We will periodically ask you to update your contact information.

Contact Times:

- Contact 1 - at hospital discharge ○ You will be asked:
  - questions about how you are doing. The questions will be about things like feeling anxious, depressed, distressed or upset.
  - questions about how you and your baby are doing together.

- questions about how much you enjoy being a parent and if you think you are good at it.
- to provide or verify contact information.

○ The total time needed to complete these questions will be about 25 minutes.

- Contact 2 - by phone or electronically at 1 month after your baby is discharged from the hospital.
  - You will be asked:
    - questions about how your baby is feeding, and about any urgent care/ER visits or hospitalizations after your baby is home.
    - to provide or verify contact information.
  - The total time needed to complete these questions will be about 10 minutes.

- Contact 3 – by phone or electronically when your baby is 3 months old.
  - You will be asked:
    - questions about how your baby acts with you and with others.
    - questions about how your baby is feeding, and about any urgent care/ER visits or hospitalizations that occurred after your baby went home.
    - questions about how your baby is sleeping at home. The questions will be about where, when and how your baby is sleeping.
    - questions about how your family is doing. The questions will be about your family environment.
    - to provide or verify contact information ○ The total time needed to complete these questions will be about 35 minutes.

- Contact 4 – by phone or electronically when your baby is 6 months old.
  - You will be asked:
    - questions about how your baby is feeding, and about any urgent care/ER visits or hospitalizations that occurred after your baby went home.
    - questions about how you are doing. The questions will be about things like feeling anxious, depressed, distressed or upset.
    - questions about how you and your baby are doing together.

- questions about how much you enjoy being a parent and if you think you are good at it.
- to provide or verify contact information ○ The total time needed to complete these questions will be about 30 minutes.

- Contact 5 - by phone or electronically when your baby is 9 months old.
  - You will be asked to provide or verify contact information

- Contact 6 – by phone or electronically when your baby is 12 months old.
  - You will be asked:
    - questions about how your baby acts with you and with others.
    - questions about how your baby is feeding, and about any urgent care/ER visits or hospitalizations that occurred after your baby went home.
    - questions about how your baby is sleeping at home. The questions will be about where, when and how your baby is sleeping.
    - to provide or verify contact information.
  - The total time needed to complete these questions will be about 25 minutes.

- Contact 7 – by phone or electronically when your baby is 18 months old:
  - You will be asked to provide or verify contact information.

- Contact 8 –when your baby is 24 months old.
  - You will be asked:
    - to bring in your baby to
      - get his/her weight, length, and head circumference
      - have your baby assessed using a series of play-based tests and questions that allow medical professionals to determine how well your baby is developing compared to other babies his/her age. The team will be checking your baby's physical, social and mental development.
    - questions about how your baby is feeding, and about any urgent care/ER visits or hospitalizations that occurred after your baby went home.

- questions about how you are doing. The questions will be about things like feeling anxious, depressed, distressed or upset.
- questions about whether you had certain bad experiences in your own childhood.

○ The total time needed to complete these questions and to have your baby checked by a medical professional will be about 2 to 3 hours.

How long will this clinical trial (study) take?

- The study will take about 24 months (2 years) to complete.
- You will be asked to answer questionnaires 6 different times. The first 2 times will be when your baby is discharged from the hospital, and 1 month after your baby has been discharged from the hospital. The other times will be when your baby is 3 months old, 6 months old, 12 months old, and 24 months old. The time needed to complete these calls or electronic sessions will range from about 10 minutes to about 35 minutes.
- You will be asked to confirm your contact information a total of 8 times. These times are when you sign the consent (during hospital stay), at hospital discharge, 1 month post-discharge, and when your baby is 3, 6, 9, 12, and 18 months old.
- When your baby is 24 months old, we will ask you to bring your baby to the clinic, medical office, or hospital for an assessment. The study team will check your baby's growth and physical, social and mental development. This check-up will include a playbased exam as well as questions. This assessment itself will take about 2 hours.
- The total amount time you will spend doing this study is about 5 to 6 hours.

What if I say no, I do not want to be in this study?

- Nothing bad will happen.
- You/your baby can still get medical care at [*insert local context here*].
- You may be asked a few questions about why you don't want to be in this study. You do not have to answer any of these questions if you don't want to.

What happens if I say yes, but change my mind later?

- You can stop being in the study at any time.
- Nothing bad will happen.
- You/your baby can still get medical care at [insert local context here].

- If you decide to stop being in the study, call *(insert head researcher name)* at *(insert phone #)*.

Will it cost me anything to be in the study?

The study will not cost you anything. You or your insurance company will be responsible for your/your baby's regular medical care, as usual.

Will I be paid for being in the study?

Yes. We will give you $50.00 for each contact time that you participate in that only involves answering questionnaires. There are 5 of these contact times (hospital discharge, 1 month post discharge, and when your baby is 3, 6, and 12 months old) that only involve answering questionnaires. You will receive $100 if you bring your baby back for a final in-person visit when your baby is 24 months old. If you participate in all 5 of the "questionnaire-only' contact times - plus the in-person visit - you will be paid a total of $350.00. This is to thank you for your time. We will *(LOCAL CONTEXT - insert time and method of payment)*. If you change your mind and decide not to be in the study, you will only be paid for the contact times for which you answered questionnaires or came for an in-person visit. You will receive your payment at – or near – the contact time you participate in.

If you receive more than $600 in one year (January-December) from *(insert local context/institution)* we may send you a tax form if required by law.

Will being in this study help me or my baby in any way?

You/your baby may not benefit directly from being in this study. Benefits could include one or more of the following:

- Feeling like you may help improve the care of other NOWS babies in the future.
- Learning more about your and your baby's well-being by responding to questionnaires that you will be asked to answer after discharge from the hospital.
- Learning more about your baby's development by responding to questionnaires that you will be asked to answer after discharge from the hospital.
- Learning more about your baby's mental and physical growth and development by getting results from the assessment done when your baby is 24 months old.
- Learning more about your family's well-being be responding to questionnaires that you will be asked to answer after discharge from the hospital.
- Depending on how you answer questions, your doctor or the study team may decide you/your baby need extra help or care to deal with issues or problems you

are having. You doctor or study team member may refer you to national helpline(s) or other service(s) that he/she believes will be able to help you.

- Being in the study may or may not help you/your baby, but the information gathered may help babies with NOWS in the future.

What are the risks of being in this study?

The risks are:

- The risks for this study are no more than what happens in everyday life for babies with NOWS.
- Someone could find out that you/your baby were in the study and learn something about you that you did not want others to know. We will do our best to protect your/your baby's privacy.
- The questions we ask may make you feel sad or upset.
- Your doctor or study team member [insert local context as appropriate – 'may' vs 'are required to'] report suspected child abuse or neglect to appropriate authorities.

What if I or my baby gets sick or hurt while in this study?

- If you get hurt when you/your baby are here for the study, we will help you get the care you need. This may include first aid, emergency care, and/or follow-up care.
- If you/your baby are not here and get hurt or sick, and think it is because of the study, do these things:
  - ✓ call your doctor or if an emergency, call 911.
  - ✓ give your doctor or ER staff
    - the name of this study *(insert name of study).*
    - the name of the head researcher for this study *(insert researcher name).*
    - a copy of this form if you have it.
  - ✓ call the head of the study *(insert researcher name and 24 hour phone #)*.
- This treatment may be billed to you or your insurance company in the normal manner. No other form of payment is available.
- INSERT ADDITIONAL LOCAL CONTEXT AS NEEDED WITH REGARD TO BILLING.

What are the alternatives to being in this study?

You do not have to be in this study.

If you do not want to be in this study, you/your baby will be treated the exact same way you/your baby would be cared for by INSERT LOCAL CONTEXT (i.e., site/medical team) if you/your baby were not asked to be in this study.

Can I/my baby be taken out of the study even if I want to continue?

Yes, the study doctor (or head researcher) can take you out of the study if:

- It is not in you/your baby's best interest to continue.
- The study is stopped for any reason.

What information will be collected about me/my baby in the study?

- General contact and background information about you/your baby, such as name, address, telephone number, and date of birth,
  - ✓ If we cannot contact you from the information that you provide, we may access your/your baby's medical record to obtain contact information from your/your baby's medical record
- Information needed to complete the questionnaires.
- Information about your baby's growth and physical and mental development.

The person who is going over this consent with you can give you details about what information will be collected if you want to know.

Who will see this information? How will you keep it private?

- The local study team will know your name and have access to your information as needed for the trial.
- We will do our best to make sure no one outside the study knows you are part of the study.

To help us stay in contact with you during the study, we may ask you if you are willing to provide name(s) and contact information of back-up contact(s). It is completely up to you to decide if you want to give us additional contact information.

- ✓ If you decide to give us information for back-up contacts, you are giving us permission to contact those person(s). If we contact one of your back-ups, that person will likely find out that you are part of this study. You may also choose to provide or share other ways for us to stay in contact with you. If you agree to do provide this information, you are giving us permission to use these ways to contact you. Someone outside of the study team may then find out that you are part of this study.

- We will take your name off of information that we collect from you during the study.
- When we share the results of the study in meetings or medical journals, we will not include your name or anything else that identifies you or your baby.
- There are people who make sure the study is run the right way. These people may see information from the study about you. They are:
  - ✓ NIH (National Institutes of Health), the study sponsor
  - ✓ OHRP (Office for Human Research Protections), a federal agency
  - ✓ University of Arkansas for Medical Sciences (UAMS) Institutional Review Board
  - ✓ Other institutional oversight offices
  - ✓ Researchers from other sites in the study
  - ✓ Research Triangle Institute (RTI)
  - ✓ IDeA States Pediatric Clinical Trial Network Data Coordinating and Operations Center at the University of Arkansas for Medical Sciences
  - ✓ Duke Clinical Research Institute Coordinating Center

  - ✓ *LOCAL CONTEXT: Insert any other applicable group that may access the records or provide oversight, including the FDA (Food and Drug Administration)*
  - ▪ LOCAL CONTEXT: Insert local state law requirements.

For example, state law requires that we report to the Arkansas Department of Health cases of certain diseases that a sick person could give to someone else. If we learn you have such a disease, we will share your name and contact information with the health department.

INCLUDE IF PART OF STATE LAW: State law requires we tell the authorities if we learn about possible child or adult abuse or that you might hurt yourself or someone else

Where and how long will my/my baby's information be kept?

- We will code your/your baby's information and keep the key to the code in a locked file or other secure location.
- Only *(insert appropriate parties)* will be able to link your information to you.
- We LOCAL CONTEXT: *will/will not* put information about you/your baby from the study in your/your baby's medical record(s). *(IF yes, include state what information will be put in the participant's medical record.)*

If I/my baby stop being in the study, what will happen to any information collected from me in the study?

- We will not be able to take your/your baby's information out of the study after the study has started.

Will my information from the study be used for anything else, including future research?

Yes. If you participate in this study, we will keep information from this research study at the Data Coordinating Center, RTI International. The information may be shared for future research as stated in the NIH (National Institutes of Health) Public Access Policy. This policy makes sure that the public has access to published results of NIH-funded research. The study will also comply with the NIH Data Sharing Policy, Policy on the Dissemination of NIHFunded Clinical Trial Information, and the Clinical Trials Registration and Results Information Submission rule. .

Information released under this policy will not identify your baby or his/her participation in this research study. Other researchers who may see the data may include people who were not part of this study.

Will you tell me the results of the study?

- We will not notify you directly, but the results of the study will be available on a website (http://www.ClinicalTrials.gov, see below) and in medical journals. You may contact us at any time during or after the study if you have questions about the results.

Will you tell me anything you learn that may impact my health?

- Yes. If we learn something about you or your baby that might be important for your or your baby's health, we will tell you.
- If the examiner finds an issue with your baby when the examiner does the assessment when your baby is 24 months old, you will be referred to your primary care provider for follow-up. The study team will talk directly to your primary care provider if you would like the study team to do this.

What if new information comes up about the study?

- We want you to know about anything that may change your mind about being in the study.
- The study team will let you know either by calling you or sending you a letter

Where can I find more information about this study?

A description of this study will be available on http://www.ClinicalTrials.gov, as required by U.S. law. This website will not include information that can identify you. At most, the website will include a summary of the results. You can search this website any time. The ClinicalTrials.gov identifier number is NCT04057820.

What if I have questions?

- Please call the head researcher of the study *(insert researcher name and phone #)*, if you
  - ✓ have any questions about this study.
  - ✓ feel you/your baby have been injured in any way by being in this study.
- You can also call the office at University of Arkansas for Medical Sciences (UAMS) that supervises research if you can't reach the study team or want to speak to someone not directly involved with this study. To do so call the UAMS Institutional Review Board at 501-686-5667.
  - ✓ You may call the UAMS IRB if you have any questions about your/your baby's rights as a research participant.
- *[insert local context if other than researcher named above]*

By signing the document, I am saying:

- ✓ I understand that joining this study is voluntary.
- ✓ I agree to be in the study and to allow my baby to be in the study.
- ✓ Someone talked with me about the information in this document and answered all my questions.
- ✓ I have been asked if I wish to talk directly to the study doctor.

I know that:

- ✓ I can stop any and all parts of the study at any time and nothing bad will happen to me or my baby.
- ✓ I can call the office that supervises research (UAMS Institutional Review Board) at 501-686-5667 if I have any questions about the study or about my rights.
- ✓ I do not give up any of my rights by signing this form.
- ✓ My decision will not change my medical care at [insert local context/site name].

I agree to be part of this study:

____
Printed Name of Participant

____ ________________
Signature of Participant Date (mm/dd/yyyy)

I agree to being contacted for future research related to this study. ___
YES ___ NO

____
Printed Name of Participant

____ ________________
Signature of Participant Date (mm/dd/yyyy)

Name/Signature of person obtaining consent:

____
Printed Name of Person Obtaining Consent

____ ________________
Signature of Person Obtaining Consent Date (mm/dd/yyyy)

List of Common Opioids

- **Brand Names (generic names):**

  ○ Demerol (meperidine) ○ Dilaudid (hydromorphone) ○ Lortab (hydrocodone) ○ MS Contin (morphine) ○ Norco (hydrocodone) ○ Opana (oxymorphone) ○ Oxycet (oxycodone) ○ Percocet (oxycodone) ○ Zohodro ER (hydrocodone)

- **Generic Names:** ○ Buprenorphine ○ Fentanyl ○ Heroin ○ Hydrocodone ○ Methadone

- **Street Names (generic names):**
  ○ Buse, Oranges, Subs (buprenorphine) ○ Apache, China Girl, Dance Fever, Friend (fentanyl) ○ China White, Dope, H. Horse, Junk, Smack (heroin) ○ Watson 387 (hydrocodone) ○ Amidone, Fizzies, Chocolate Chip Cookies (methadone) ○ M. Miss Emma, Monkey, White Stuff (morphine)

- Hillbilly Heroin, O.C. Oxycet, Oxy (oxycodone)
