# Supplementary material for: Eating, Sleeping, Consoling for Neonatal Opioid Withdrawal (ESC-NOW): a Function-Based Assessment and Management Approach study protocol for a multi-center, stepped-wedge randomized controlled trial
Source: Trials. 2022 Aug 9;23:638. doi: 10.1186/s13063-022-06445-z (PMC9361241; doi:10.1186/s13063-022-06445-z)
Supplement: Supplementary file 3 — Additional file 3. [file 13063_2022_6445_MOESM3_ESM.pdf]

Summary of protocol changes following the start of enrollment

[Version 05 (published protocol) to Version 07]

Summary of Key Changes from Version 05 to Version 06:

Affected Section(s)	Summary of Revisions Made (05 to 06)	Rationale
Waiver of Consent section	To last paragraph in section:<br>• Added option to post handout materials.	Additional flexibility for sites.

Summary of Key Changes from Version 06 to Version 07:

Affected Section(s)	Summary of Revisions Made (06 to 07)	Rationale
Training and Implementation subsection	• Deleted “review of instructional manual”<br>• Deleted “co-assess cases with the “gold-star raters” and added reference to Training & Implementation Manual, instead of MOP<br>• Replaced protocol study team with site research team<br>• Added the number of gold-star raters (3) that will be assessed each period to ensure fidelity.<br>• Added “training milestones” to the criteria to assess for before moving to the ESC intervention period.<br>• Renamed webinars to coaching calls and deleted “biweekly.”	Protocol clarifications and internal consistency; study procedure changes
Page 24, line 523	• Changed section title to ESC Intervention Periods	Consistency with other study periods
Page 24 Intervention subsection; lines 577-587	• Replaced protocol study team with site research team<br>• Deleted “study” and added “ESC intervention”<br>• Deleted “assessing 10 individuals”<br>• Added “NOWS infant, written cases”<br>• Deleted “through a centralized training platform” for utilizing just-in-time training	Protocol correction and clarification; add flexibility
Intervention subsection; lines 588-596	• Deleted “the protocol study team will develop an electronic platform that will allow…”<br>• Deleted ”The electronic platform will contain items from the ESC IRR tool (see MOP)”<br>• Added “keep completed ESC Implementation Process Evaluation Forms with other study documentation”<br>• Deleted sentence that stated sites will enter implementation process evaluation data into the electronic application and will send it directly to a central repository	Clarify and correct process
