# Supplementary material for: Eating, Sleeping, Consoling for Neonatal Opioid Withdrawal (ESC-NOW): a Function-Based Assessment and Management Approach study protocol for a multi-center, stepped-wedge randomized controlled trial
Source: Trials. 2022 Aug 9;23:638. doi: 10.1186/s13063-022-06445-z (PMC9361241; doi:10.1186/s13063-022-06445-z)
Supplement: Supplementary file 4 — Additional file 4. [file 13063_2022_6445_MOESM4_ESM.pdf]

Eating, Sleeping, Consoling for Neonatal Opioid Withdrawal (ESC-NOW): a Function-Based Assessment and Management Approach

Intervention Training and Implementation Manual

Version 05

Final: 09/22/2020

Revised: 10/29/2021

Contents

1. Summary of Revisions 3
2. Abbreviations and Definition of Terms 5
   2.1 Abbreviations 5
   2.2 Definitions 5
3. ESC Intervention Training and Implementation Manual 7
4. COVID-19 Related Changes 7
   4.1 Key Training and Implementation Contacts 7
5. ESC Intervention Training 7
   5.1 Overview 7
   5.2 ESC Intervention Training Matrix 0
   5.3 Virtual Site Champion Training 0
      5.3.1 Goal 1
      5.3.2 Who Attends 1
      5.3.3 Preparation for Virtual Site Champion Training 1
      5.3.4 3-Day Virtual Site Champion Training Agenda 2
      5.3.5 Virtual Site Champion Training Tracking 3
   5.4 Onsite Clinical Team Training 3
      5.4.2 Preparing for Onsite Training 4
      5.4.3 General Approach to Onsite Training 4
      5.4.4 Training Resources 4
      5.4.5 Nurses 5
      5.4.6 Onsite Training Tracking 7
      5.4.7 Other Providers, Residents, Site PI, and Research Coordinators 8
      5.4.8 Just-in-time Training 8
6. ESC Implementation 9
   6.1 Preparing for ESC Implementation 9
      6.1.1 Clinical Readiness 10
      6.1.2 Algorithm 10
      6.1.3 Electronic Medical Record/Documentation 10
   6.2 ESC Implementation Tool Kit 10
   6.3 ESC Implementation Optimization 10
      6.3.1 Sample Meeting Schedule 11
7. Maintenance of Fidelity 11
   7.1 ESC Inter-rater Reliability (IRR) 12
      7.1.1 During Implementation Phase of Transition Period 12
      7.1.2 During ESC Intervention Periods 12

7.2 ESC Implementation Process Evaluation 13
7.2.1 During ESC Intervention Periods 13
8. Entering site-level deviations in REDCap 13
Appendix A: ESC Care Tool and definitions 16
Appendix B: ESC IRR Tool 18
Appendix C: Conversion of Pharmacological Treatment Algorithms 19
Morphine 19
Methadone 21
Buprenorphine 22
Appendix D: ESC Care Tool Epic Tip sheet 23
Appendix E: ESC Care Tool Cerner Tip Sheet 24
Appendix F: Parent Education Brochure 25
Appendix G: Newborn Care Diary Sample Pages 26
Appendix H: Implementation Guide 27
Appendix I: Ensuring Fidelity of IRR Scores 28
Video and Written Cases 28
Completing IRR on Paper 30
Appendix J: ESC Implementation Process Evaluation form 31
Appendix K: ESC Trial Schedule 32

1. SUMMARY OF REVISIONS

Date	Section	Change Details
11/02/2020	All	Corrected references to section numbers and changed virtual ESC simulation training to virtual site champion training
	5, 9.1	Renamed biweekly webinars to biweekly implementation coaching sessions
	7.1.6	Clarified onsite training tracking process
	9.4	Corrected appendix reference. Updated and clarified implementation process evaluation
	10.2	Added that sites will complete the IRR assessment with an infant with NOWS
	10.2.2	Added instructions for the IRR assessment process
	Appendix G	Renamed to clarify when process is used
	Appendix J	Added to provide links to cases and IRR for use during implementation phase of intervention period
05/11/2021	2.2	Minor updates to care team and inpatient clinical team definitions
	5.2	Moved summary table to this section
	5.3.2	Clarified qualifications for gold-star rater<br><br>Clarified number of site champions per site
	5.3.3.1	Updated amount of time preparatory work will take
	5.3.4	Updated and clarified the 3-Day Virtual Site Champion Training Agenda
	5.4.3.3	Streamlined the "Encouraged" part of the preparatory modules summary table<br><br>Updated amount of time it will take to complete these modules and Video Case 1
	5.4.3.4	Added remediation subsection
	5.4.3	Added when site no longer need to send weekly training reports
	5.4.4.2	Updated amount of time it will take to complete the preparatory modules
	6.1	Clarified milestones for clinically implementing the ESC Care approach
	6.3	Updated and defined types of meetings and when they will occur
	7.1	Added when IRR assessments will need to be conducted and number of gold-star raters and nurses who need to complete the IRR assessments<br><br>Added that sites can complete IRR assessment with written or video cases or with a NOWS infant<br><br>Added the number of gold-star raters and nurses who need to complete the IRR each period
	7.2	Clarified process for completing the Implementation Process Evaluation Form
	Appendix C	Moved pharmacological treatment algorithms to Appendix C

		Updated contact information, clarified process, new algorithm examples
	Appendix H	Moved Implementation Guide to this appendix
	Appendix I	Updated and simplified process for ensuring fidelity of IRR scores
	Appendix J	Moved ESC Implementation Process Evaluation Form to this appendix
07/15/2021	5.4	Added information from Site Champion Training slides
	Appendix K	Added
10/29/2021	5.1, 5.3.1, 5.4.2, 5.4.3, 5.4.8	Clarified text
	5.3.4.2	Changed interrater reliability tool (IRR) testing to gold-star rater testing
	5.4.4	Deleted table; added list of all training resources and link to Onsite Training Packet that contains all training resources
	5.4.5.4	Changed formal IRR testing to formal ESC testing
	5.4.6	Added REDCap link to upload weekly training reports<br>Changed IRR testing to ESC assessments on Written Cases 5-7
	5.5	Deleted subsection
	7.1.1 & 7.1.2	Specified the cases that must be used for IRRs<br>Clarified process
	7.2 & 7.2.1	Added section detailing process for completing the ESC Process Evaluation Form
	Section 8	Added section with instructions on how to enter a deviation into REDCap
	Appendix I	Specified cases that must be used for IRRs and IRRs completed on paper at the site<br>Updated instructions for completing IRRs

2. ABBREVIATIONS AND DEFINITION OF TERMS

2.1 Abbreviations

DCC	Data Coordinating Center
DCOC	Data Coordinating and Operations Center
DCYF	Division for Children, Youth, and Families
EMR	Electronic Medical Record
ESC	Eat Sleep Console
IRR	Inter-rater reliability
ISPCTN	IDeA States Pediatric Clinical Trials Network
NAS	Neonatal Abstinence Syndrome
NOW	Neonatal Opioid Withdrawal
NOWS	Neonatal Opioid Withdrawal Syndrome
NPI	Non-Pharm Care Interventions
PI	Principal Investigator
REDCap	Research Electronic Data Capture

2.2 Definitions

Analysis team- The team(s) of individuals from the DCC who will provide detailed examination and statistical analysis of the primary and secondary outcomes and endpoints of the study.

Clinical monitoring team- Site monitors and support staff that will assist sites in the conduct of the trial and will monitor compliance with the study protocol.

Care team- Inpatient clinical team and the primary caregiver(s) of each infant participating in the study.

Inpatient clinical team: A multidisciplinary team of health care professionals who provide clinical care for infants with NOWS at each participating site including nurses, physicians, social workers, etc. This group will routinely assess infants with NOWS with the sites' assigned care approach and will work together to determine the need for both non-pharmacologic and pharmacologic treatment for affected infants. Also referred to as "clinical team."

ESC care approach - A care approach that emphasizes parental involvement, simplifies the assessment of infants with NOWS using the ESC Care Tool and focuses interventions on non-pharmacologic therapies.

ESC Care Tool – A function-based assessment and management tool for evaluating the withdrawal severity and guiding management of infants with NOWS based on an infant's ability to eat, sleep, and be consoled.

ESC faculty – The group of individuals who will lead the site champions in virtual training and provide support for implementation of the ESC care approach at the sites.

ESC inter-rater reliability (IRR) tool – A six-item assessment completed by individuals training/trained to use the ESC Care Tool to ensure consistency across assessors.

Gold-star raters – Site champions and other individuals who have consistently achieved 100% reliability on written and/or video assessment cases (6/6 items on the ESC IRR tool).

Implementation phase – Occurs during the transition period and begins once a site is cleared to clinically use the ESC care approach. Data collection will not begin until the site moves into the first ESC intervention period.

Primary caregivers – Infant's parent, grandparent, or guardian.

Protocol study team- The group of individuals who designed the study protocol and who will assist sites in answering protocol questions. Also referred to as "study team."

Site champions - A core group from each site, which may include clinical nurses, nurse educators, advanced practice providers, and physicians, that the ESC faculty will train in the use of the ESC care approach. Site champions will become gold-star raters and will train all other clinical team members who assess infants with NOWS at their site. Site champions will also facilitate implementation of the ESC care approach at their site.

Site research team- The research team at each participating site. This includes the site principal investigator (PI), co-investigators, research coordinators, and other members of the local research team, which may include, but are not limited to, research nurse/manager, data coordinator/manager, research assistant, regulatory coordinator/manager, developmental specialist(s), and interns/students.

Training platform – ISPCTN research portal where training materials are housed.

Transition period – The study period when ESC training and clinical implementation of the ESC care approach will occur. Site champions, trained just prior to the transition period, will train their site in ESC Care Tool use and ready the site for/support the site through implementation of the ESC care approach.

3. ESC INTERVENTION TRAINING AND IMPLEMENTATION MANUAL

This manual will provide a detailed overview of the ESC intervention training (online preparatory modules, virtual site champion training, onsite ESC Care Tool training, and just-in-time training) and ESC care approach implementation. For details specific to the ESC study protocol and protocol training, please refer to the ESC study protocol, manual of operations, and other supporting documents.

4. COVID-19 RELATED CHANGES

Due to COVID-19, ESC intervention training will include virtual training of site champions and increased site implementation support from ESC faculty. All training materials will be available on the training platform (you must be logged on to the training platform for this link to work).

4.1 Key Training and Implementation Contacts

Name	Email	Role
Leslie Young	****	Lead Study Investigator
Jessica Snowden	****	Operational Principal Investigator
Kathy Edwards	****	ESC Project Lead
DeAnn Hubberd	****	ESC Training Project Lead

5. ESC INTERVENTION TRAINING

5.1 Overview

Approximately 2 weeks before entering their designated transition period (see section 5.3), site champions will complete detailed training covering the use of the ESC Care Tool in a clinical setting. Onsite training will occur soon after the site enters the transition period. ESC intervention training includes online preparatory modules, virtual site champion training, onsite training, coaching sessions, and just-in-time training. Figure 1 below provides a more detailed overview of planned ESC training activities throughout the duration of the study.

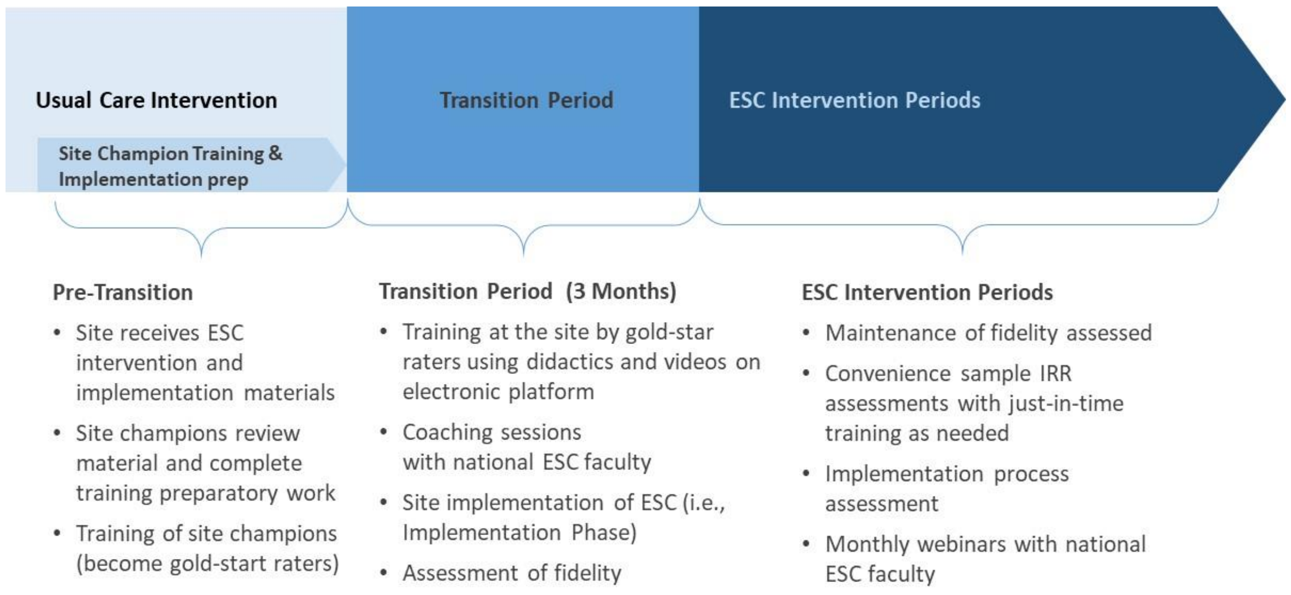

Figure 1. ESC Study Periods

5.2 ESC Intervention Training Matrix

The ESC intervention-training matrix (Figure 2, below) outlines the training requirements for individuals in various research and clinical roles at the site.

Please note that the ESC Care Tool and associated training materials cannot be shared outside the clinical trial/participating centers without special permission from one of the ESC Tool developers.

Training Requirements	Site Champions (section 5.3)	Nurses (sections 5.4.3)	Other Providers: Physicians, PA/NP, Fellows, etc. (section 5.4.4)	Residents (section 5.4.4)	Site Principal Investigator (section 5.4.4)	Coordinator (section 5.4.4)
Online preparatory modules	X*	X	X	R	X	X
Virtual site champion simulation training	X					
Onsite ESC training		X Group session (local)				
Video and/or written cases (online)**		X Group session (local)	R[1]	R[1]	R[1]	
Just-in-time training†	X	X				
X=required; R=recommended; *Completed before virtual site champion training; [1]One Case; †Completed as needed and/or as desired; **Component of onsite ESC training						

Figure 2. ESC Training Matrix

5.3 Virtual Site Champion Training

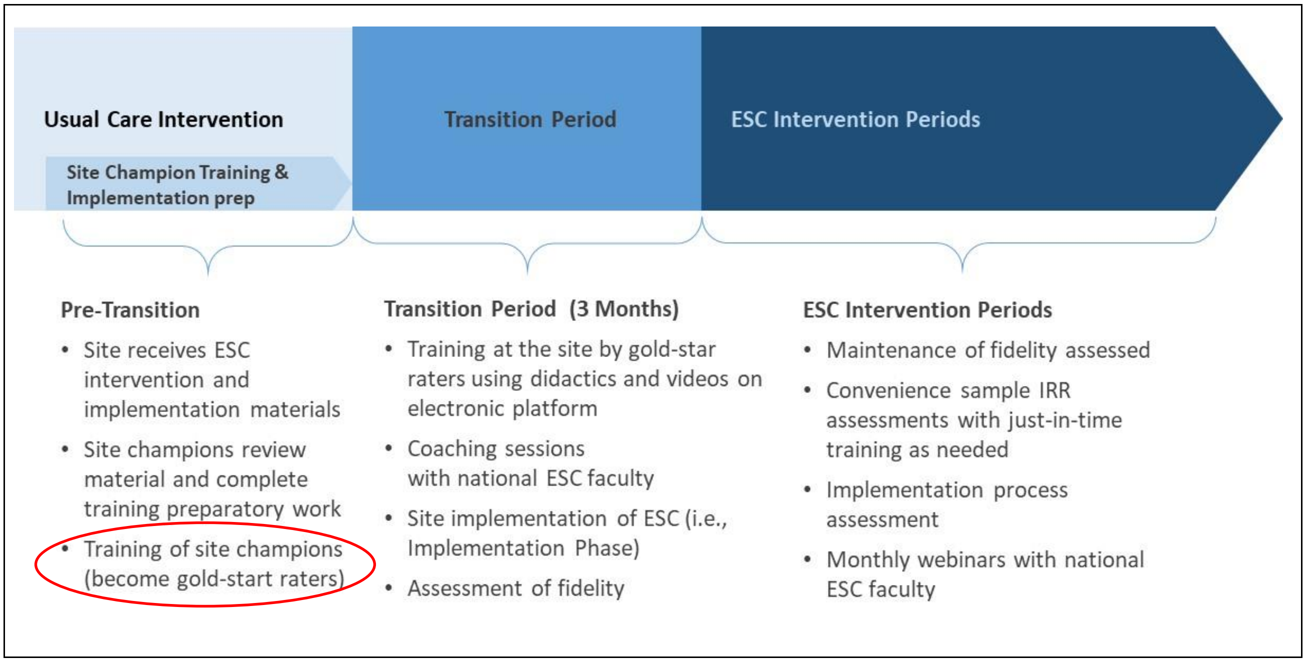

5.3.1 Goal

The goal of virtual site champion training is to provide site champions with intensive training about using the ESC Care Tool (Appendix A) in a clinical setting, certify site champions as gold-star raters, as well as facilitate on-site training and implementation of the ESC care approach. To become a gold-star rater, site champions must achieve and maintain 100% IRR on 3 cases using the ESC IRR tool (Appendix B).

Who attends	See section 5.3.2
What (content)	• Simulated patient experiences to apply ESC Care Tool<br>• Assessment of inter-rater reliability<br>• Implementing the ESC care approach at your site<br>• Trauma-informed care and communication
When	In the weeks immediately preceding the sites entry into the transition period.

5.3.2 Who Attends

Each site should have 6 site champions. There will be approximately 18 to 24 site champions (representing 3 to 4 sites) at each virtual site champion training. Most participating sites have two units providing care for infants with NOWS. Ideally, for these sites, 3 site champions (see definition in section 2.2) from each of the two units will attend. However, if 3 units provide care for NOWS, it is acceptable to send 2 participants per unit. Listed below are the minimum recommendations for potential ESC site champions.

- one nurse leader/nurse educator
- one infant provider (MD/PA/NNP) who can provide support for transition/implementation in all care environments involved
- one bedside nurse from each care unit

Additional members can include (but are not limited to)

- Nurse educator
- Clinical nurse specialist
- Clinical quality improvement specialist for newborn care
- Additional bedside nurses and infant providers

5.3.3 Preparation for Virtual Site Champion Training

5.3.3.1 Online Preparatory Work

To optimize the training experience for all participants, it is essential that site champions complete the written case, preparatory modules, and video case outlined below **before** the virtual site champion training. The virtual site champion training does not impart the basics of the ESC Care Tool or the ESC care approach. The study team and ESC faculty expect that site champions will come to the virtual site champion training with the general baseline knowledge of the ESC Care Tool and ESC care approach that the written case, modules, and video case provide. Preparation before the virtual site champion training will allow sites to optimize their training experience and minimize the need for additional training.

Please note that the ESC Care Tool and associated training materials cannot be shared outside the clinical trial/participating centers without special permission from one of the ESC Tool developers.

What (content)	• Required<br>○ Written Case 1 baseline IRR assessment<br>○ Modules<br>▪ Using the ESC Care Tool in Care of Opioid-exposed Newborns Presenter: Dr. Kate MacMillan<br>▪ Trauma Informed Care: Providing Compassionate Evidence-Informed Care for Infants and Families Presenter: Dr. Lenora Marcellus<br>▪ General Care of the Infant with Neonatal Opioid Withdrawal Syndrome Presenter: Dr. Elisha Wachman<br>○ Video Case 1<br>○ Written Case 1 post IRR assessment<br>• Encouraged<br>○ Development of the ESC Care Tool for Care of Opioid-exposed Newborns Presenter: Dr. Bonny Whalen
When	• Before virtual site champion training; during the transition period<br>• Time ~2.5 hours
Where	• REDCap and training platform
How	• Can be completed independently or as a group, as site guidelines allow<br>▪ Examples:<br>As part of a weekly meeting with modules completed sequentially<br>As one long group meeting<br>As a grand round

5.3.4 3-Day Virtual Site Champion Training Agenda

5.3.4.1 DAY ONE (approximately 3 hours contact time)

- Faculty & Hospital Team Intros, 3-Day Training Overview, & Goal Setting
- Written Case 1 Group IRR Results & Brief Review of ESC Care Tool
- Use of the ESC Care Tool with Written Cases 2-4 and Group IRR
- Overview of Day 2 and Q&A

5.3.4.2 DAY TWO (approximately 4 hours contact time)

- Day 1 Self-Reflections & Brief Review of ESC Simulation Cases/Facilitated Sessions (Optional)
- Simulation Cases and Facilitated Session: Solution-Focused Dialogue & Compassionate Care

    - Hospital teams will rotate through 3 ESC simulation cases (with each site champion serving once as a direct in-room assessor and twice as an indirect assessor) and a facilitated session about solution-focused dialogue and compassionate care for families of opioid-exposed newborns.
- Simulation Case Debriefing
- Group Discussion of Simulation Case Experience with Trauma-Informed Lens
- Review Homework and Gold-Star Rater Testing
    - Homework: Written Cases 5-7 gold-star rater testing

5.3.4.3 DAY THREE (approximately 3 hours contact time)

- Day 3 Overview and Goals
- Written Cases 5-7: Review Results
- Video Case 5: Group Review & IRR
- Rooming-In & Parental Presence Challenges: Brainstorming Solutions
- Training Resources & Documentation
- Brainstorming First Steps with ESC Faculty Coaches
- Wrap Up/Evaluation

5.3.5 Virtual Site Champion Training Tracking

The DCOC will track completion of the preparatory modules and virtual site champion training. At the end of virtual site champion training, site champions will receive a training sign-off sheet showing training completion. Site champions will also receive continuing education credit for virtual site champion training.

5.4 Onsite Clinical Team Training

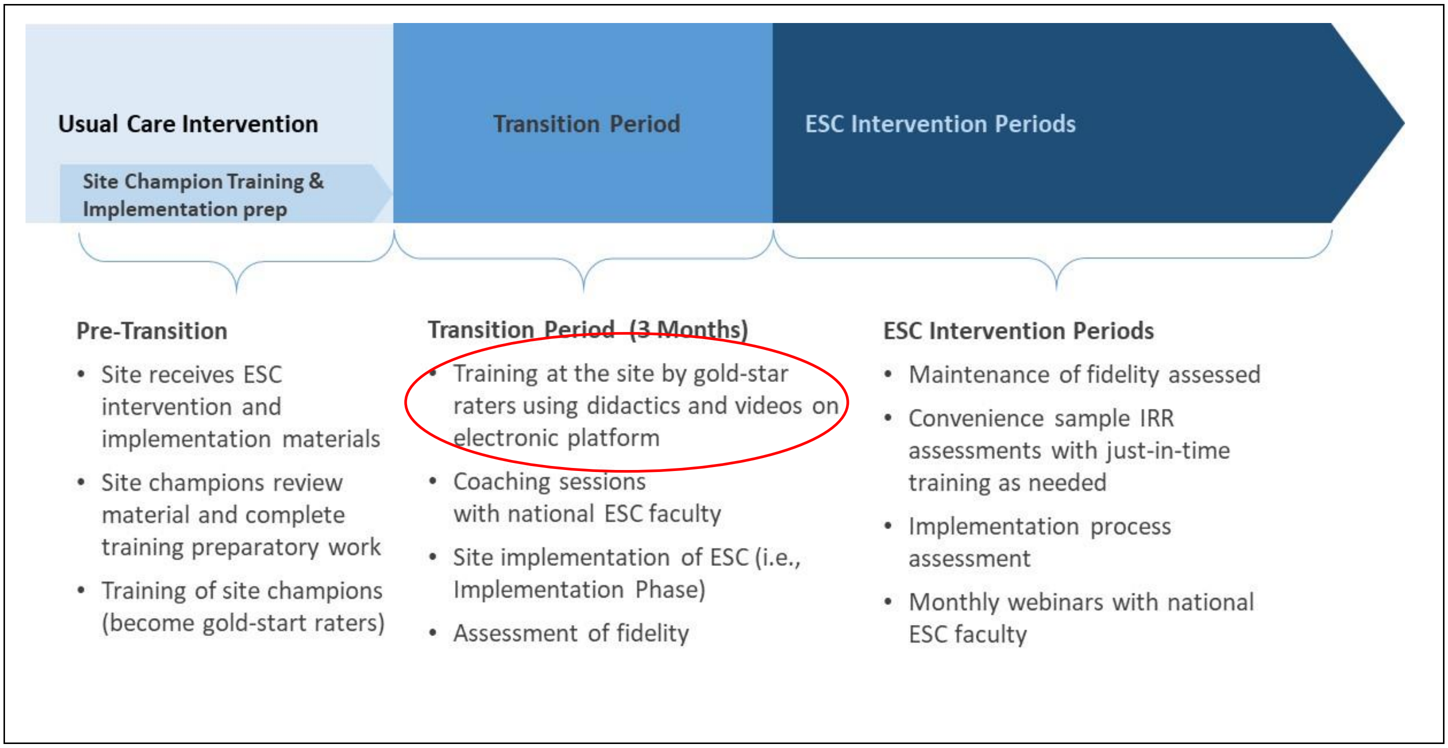

5.4.2 Preparing for Onsite Training

The ESC study team allows flexibility in onsite training approaches, if sites meet the minimum online training requirements. Listed below are some things to consider when preparing for onsite training.

1. Will your site meet in person, remotely (i.e., via Zoom), or a hybrid approach? In the COVID era, many sites hold trainings remotely or use a hybrid approach by holding some of the training remotely and then meeting in small groups to discuss cases and outcomes.
2. How will trainees access the preparatory work? Some sites ask trainees to access the preparatory modules and Video Case 1 via the training platform, and some sites house the modules and Video Case 1 on their learning management system. Both approaches are fine, and the DCOC can assist your site with the logistics of either approach.
3. How will trainees complete Written Case 1? Sites have used different approaches for this. Some use the site's REDCap system to send Written Case 1 as a survey, others ask trainees to complete Written Case 1 on paper or via the site's learning management system.
4. How have other sites approached training that have been successful for them?

5.4.3 General Approach to Onsite Training

- Gold-star raters will customize the order of video and written cases and can develop their site-specific training plan with the support of the ESC faculty. The study team recommends that sites maintain a record of training activities and have this available for the study team and site monitoring.
- During onsite training, sites will use a paper version of the ESC IRR tool or ESC Care Tool. Gold- star raters will use Written Cases 5-7 to assess the nurses' ability to accurately evaluate NOWS babies using the ESC Care Tool. Sites will maintain the completed ESC Care Tool or ESC IRR tool with study records for monitoring visits. See section 5.4.6 for how clearances and ESC assessment scores are tracked and reported.
- The protocol study team requests that nurses who do not attain ≥80% on each written case (Written Cases 5-7) complete supplementary remediation. Nurses hired after the initial site training will complete the educational modules and ESC just-in-time training inclusive of co-assessing cases with gold-star raters using the ESC IRR tool or ESC Care Tool to demonstrate ≥80%.
- If a nurse achieves 100% on each written case (Written Cases 5-7), the site research team can consider him/her a gold-star rater and may ask the nurse to function in this capacity. Site staffing levels will determine the number of gold-star raters at a site, with the goal of having one gold-star rater available on each shift.

5.4.4 Training Resources

All training resources listed below can be accessed in the Onsite Training Packet

1. ESC Care Tool and associated training materials license
2. ESC Care Tool
3. ESC IRR Tool
4. Video Lectures (i.e., Preparatory Modules)

- Mp4 files available for use at site (e.g., with learning management system) or can access via ISPCTN research portal/training portal. Most sites have found it easiest to download the mp4 files and use them at their site.
    a. Using the ESC Care Tool in Care of Opioid-exposed Newborns
    Presenter: Dr. Kate MacMillan
    b. Trauma Informed Care: Providing Compassionate Evidence-Informed Care for Infants and Families
    Presenter: Dr. Lenora Marcellus

c. General Care of the Infant with Neonatal Opioid Withdrawal Syndrome
Presenter: Dr. Elisha Wachman

d. Development of the ESC Care Tool for Care of Opioid-exposed Newborns
Presenter: Dr. Bonny Whalen

5. Written Cases
   a. Case 1 with teaching script and REDCap zip file
   b. Cases 2-4 with algorithms, teaching scripts, and REDCap zip file
   c. Cases 5-7 with algorithms and REDCap zip files
   d. Case 8 with algorithm, teaching script, and REDCap zip files
6. Video Cases 1-5

- Mp4 files available for use at site (e.g., with learning management system) or can access via ISPCTN research portal/training portal. Most sites have found it easiest to download the mp4 files and use them at their site.

7. General ESC decision making algorithm
8. Handouts from 3-Day Site Champion Training Folder
9. Foundational components and reading/resource list for implementing the ESC care approach

5.4.5 Nurses

5.4.5.1 Goal

The goal of onsite nurse training is to provide the training necessary for nurses to assess and manage infants with NOWS using the ESC Care Tool. Please note that the ESC Care Tool and associated training materials cannot be shared outside the clinical trial/participating centers without special permission from one of the ESC Tool developers.

5.4.5.2 Who Attends

Nurses who provide care for infants with NOWS and assess the severity of their withdrawal to facilitate clinical management.

5.4.5.3 Preparatory Modules

To optimize the training experience for all participants, it is essential that nurses watch the preparatory modules before training. The modules provide a general knowledge of the ESC Care Tool and ESC care approach, and nurses are expected to come to training with this knowledge. Onsite training plans will be discussed during the site champion training. See section 5.4.5.4 for onsite training outlines.

Who	• Clinical and research staff affected by ESC training
Where	• Online via the training platform or site learning management system
What (content)	• Required<br>○ Using the ESC Care Tool in Care of Opioid-exposed Newborns<br>Presenter: Dr. Kate MacMillan (~55 minutes)<br>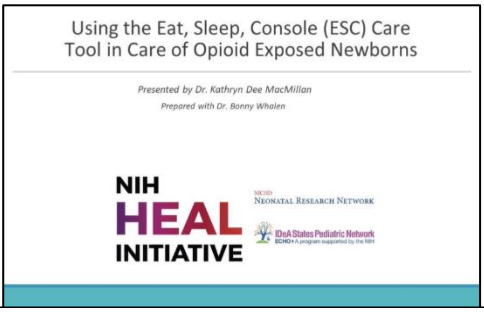

<table>
<tr><td></td><td>
○ Trauma Informed Care: Providing Compassionate Evidence-Informed Care for Infants and Families
Presenter: Dr. Lenora Marcellus (~22 minutes)
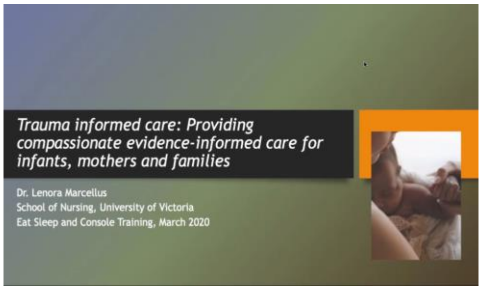

○ General Care of the Infant with Neonatal Opioid Withdrawal Syndrome
Presenter: Dr. Elisha Wachman (~22 minutes)
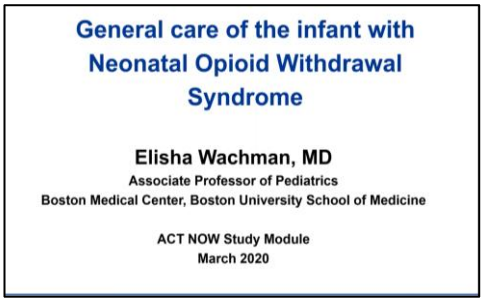

• Encouraged
○ Development of the ESC Care Tool for Care of Opioid-exposed Newborns
Presenter: Dr. Bonny Whalen
</td></tr>
<tr><td>When</td><td>• During the transition period (before site training)</td></tr>
<tr><td>How</td><td>• Can be completed independently or as a group, as site guidelines allow
▪ Examples:
As part of a weekly meeting with modules completed sequentially
As one long group meeting
As part of a trainee lecture series
As a grand round</td></tr>
</table>

5.4.5.4 Minimum Onsite Training Requirements

The target audience is bedside staff performing ESC assessments. The minimum training requirements are listed below. Site can include additional training materials, as desired.

Case 1 (approximately 1 hour)

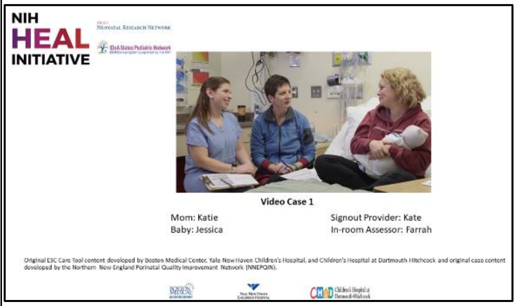

- Written Case 1: baseline assessment (5-10 minutes)
- Video Case 1 (approx. 30 minutes)
- Written Case 1: post- training assessment (5-10 minutes)

Review Cases (approximately 1 hour)

- Written Cases 2-4 with training model individualized to site (1 hour)
    e.g., group review/IRR/debriefing or one-on-one IRR with gold-star rater and debriefing

Formal ESC Testing using Written Cases 5-7 (30 minutes - 1 hour)

- Must complete within one week of onsite training
- Nurses must score ≥80% on each of the 3 written cases (i.e., Written Cases 5-7) prior to being cleared for bedside use of ESC Care Tool

Remediation

If nurses do not achieve ≥80% on Written Cases 5-7, the site can give them a remediation case that has the same learning points as the missed case and/or provide education specific to the missed teaching points to ensure the concept is understood. If nurses score ≥80% on the remediation case, they can be cleared for bedside use of the ESC Care Tool. Review of missed concepts is an important part of this process.

Note: If nurses score 100% on 2 cases of the 3 cases (5-7) and score 100% on a remediation case, these nurses can be gold-star raters.

5.4.6 Onsite Training Tracking

Each Thursday, a site research team member will upload the following information to REDCap. Sites can stop sending weekly training reports once they are cleared for clinical implementation of the ESC care approach.

1. Who completed the following activities
    a. online preparatory modules
    b. ESC intervention training
    c. ESC assessments on Written Cases 5-7
        i. Keep score results at the site for review during audits/site visits
        ii. Individual scores do not need to be submitted in the weekly report, only note if the person passed/failed or is a gold-star rater

The DCOC will use this information to track site training, formal ESC assessments, and gold-star rater clearance. The DCOC will also track modules that trainees complete independently on the training platform. For modules completed by a group, sites will track the individuals present to document their participation.

Sample Weekly Training Report

Clinical Team Member	Modules Complete?	ESC Intervention Training Complete?	Formal ESC Assessment Pass/Remediate?	Gold Star Rater?
Sharon Stoolman	Yes	Yes		
Jay Snow	Yes	Yes	Remediate	
Lisa Sziecowski	Yes	Yes	Pass	Yes
Rusty McCulloh				

5.4.7 Other Providers, Residents, Site PI, and Research Coordinators

5.4.7.1 Goal

The goal of training for other providers and residents who participate in the management (but not assessment) of infants with NOWS with the fundamentals of the ESC care approach appropriate for their clinical role. This will allow a cohesive clinical-team approach for management of infants with NOWS.

The goal of training for the site PI and research coordinators is to provide an overview of the ESC training content.

5.4.7.2 Who Attends

Clinical team members who participate in the management of infants with NOWS, site PIs, and research coordinators. Physicians, physician assistants, nurse practitioners, fellows, residents, site PIs, and research coordinators should complete modules 1-3, listed below. Module 4 is optional but recommended.

It is also recommended that all members of the clinical team who participate in the management of infants with NOWS, site PIs, and research coordinators complete one of the video and/or written case examples to better understand how the tool is operationalized.

What (content)	1. General Care of the Infant with Neonatal Opioid Withdrawal Syndrome<br>Presenter: Dr. Elisha Wachman<br>2. Trauma Informed Care: Providing Compassionate Evidence-Informed Care for Infants and Families<br>Presenter: Dr. Lenora Marcellus<br>3. Using the ESC Care Tool in Care of Opioid-exposed Newborns<br>Presenter: Dr. Kate MacMillan<br>4. Development of the ESC Care Tool for Care of Opioid-exposed Newborns<br>Presenter: Dr. Bonny Whalen*
When	• During the transition period, before clinical implementation of ESC<br>• Module time ~1.75 hours
Where	• Online via the training platform or site training platform
How	• Can be completed independently or as a group, as site specific guidelines allow
* This module is optional	

5.4.7.3 Tracking

See section 5.4.6

5.4.8 Just-in-time Training

The goals of just-in-time training is to offer additional resources to gold-star raters who do not maintain 100% on IRRs and nurses who do not maintain ≥80% IRRs (Section 7) and to offer these team members the opportunity to regain the required score. Other goals are to offer training resources for nurses hired after the initial onsite training and for nurses who may not have used the ESC care approach for some time.

Who	• Clinical team members who do not score ≥80% on IRRs, as noted in the protocol<br>• Gold-star raters who do not score 100% IRRs, as noted in the protocol

	• Clinical team members who did not receive initial site ESC training*<br>• Clinical team members who have not recently cared for an infant with NOWS and desire a refresher
Where	• Online via the ISPCTN training platform or site learning management system
How	• Customized to site and individual, examples include:<br>★ Plan 1: Review video cases 2-4 with ESC IRR tool exercises (Written Case 5-7 or Written Case 8)<br>Plan 2: Review video cases 2-4 with Gold Star Rater, identify specific items with lower agreement and develop targeted coaching<br>Plan 3: Role play written case teaching scripts with direct feedback/targeted coaching from Gold Star Rater for items of lower agreement<br>Plan 4: Co-assess cases with Gold Star Raters using the ESC IRR tool or ESC Care Tool until able to demonstrate 100% (Gold Star Raters) or 80% (Bedside Nurses) agreement
* These individuals should also watch the ESC Care Tool online module and are encouraged to watch the other online modules in addition to completing the just-in-time training	

6. ESC IMPLEMENTATION

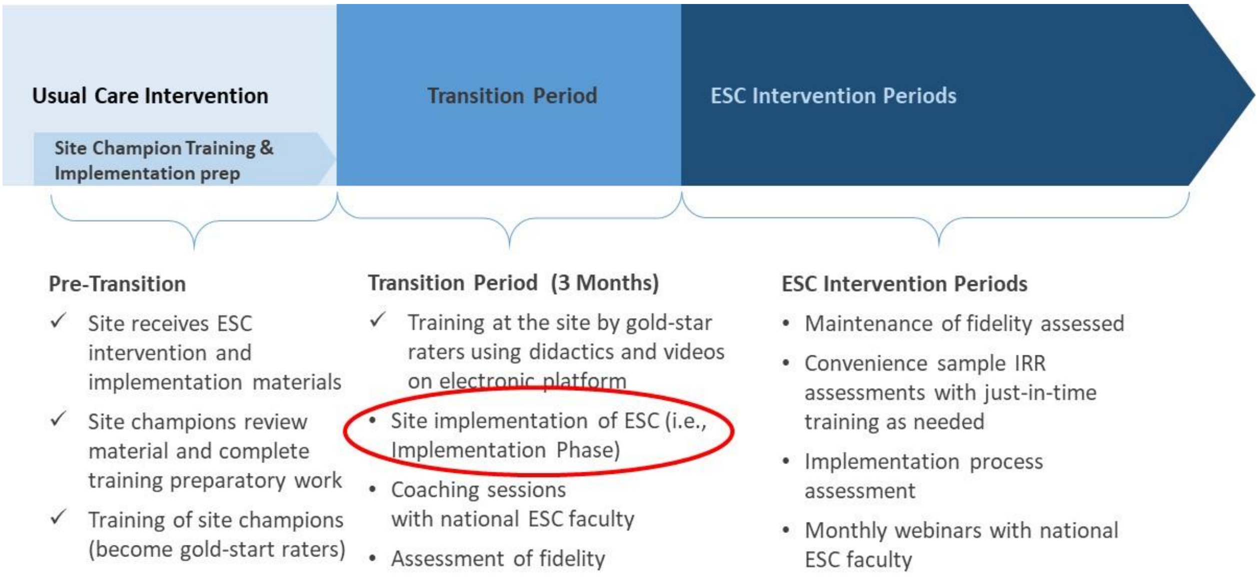

6.1 Preparing for ESC Implementation

The study team advises sites to clinically implementing the ESC care approach during the Transition Period, so that sites can "practice" the ESC care approach before the first ESC period, when data collection will restart. A study team

member will clear sites for clinical implementation of the ESC care approach. Sites must meet the clinical readiness threshold and algorithm requirement before a study team member can clear a site for clinical implementation (see details below).

6.1.1 Clinical Readiness

A site can clinically implement the ESC care approach when it has trained and cleared enough nurses for independent use of the ESC care tool to consistently implement the ESC care approach. To clear nurses for independent use of the ESC Care Tool, nurses must score ≥80% reliability on the IRR tool or ESC Care Tool for 3 written cases (see section 5.4.5.4).

6.1.2 Algorithm

A site must submit its ESC treatment algorithm that shows how the site revised its pharmacological treatment algorithm to an ESC treatment algorithm. A study PI must approve the ESC treatment algorithm before the study team can clear the site to clinically implement the ESC care approach. See Appendix C for algorithm conversion samples.

6.1.3 Electronic Medical Record/Documentation

The study does not require sites to include the ESC Care Tool in its electronic medical record (EMR). If a site does not include the ESC Care Tool in its EMR, the site should implement a documentation process during the transition period.

6.1.3.1 Epic EMR Tip Sheet

Please see Appendix D for the EPIC tip sheet.

For questions regarding the ESC Care Tool EMR build in Epic, please contact the Epic analysts ****. When emailing ****, please be sure to copy Leslie Young. The subject line should indicate that you are part of the ACT NOW ESC study.

6.1.3.2 Cerner EMR Tip Sheet

Please see Appendix E for Cerner tip sheet.

6.2 ESC Implementation Tool Kit

The following items will be available to nurses and clinical team members for implementation of the ESC care approach.

- **ESC Care Tool**
- **ESC IRR**
- **Parent education brochure.** The parent education brochure (Appendix F) is a tool sites can use when collaborating with the primary caregivers in the care of their infants. It is designed to help primary caregivers learn the best ways to care for their infants.
- **Newborn care diary.** The clinical team will encourage the primary caregiver(s) to record their infant's feedings (timing and duration, and/or volume), sleeping (quality and quantity), and ability to be consoled, in the Newborn Care Diary (Appendix G). The clinical team should encourage parents/primary caregivers to document ESC behaviors in the newborn care dairy through the infant's first 7 days of life (or until the time of discharge). Many families will benefit from continuing to use the Newborn Care Diary beyond this point.
- **Implementation Guide.** Sites can use the implementation guide (Appendix H) as a quick guide to ensure that they have all steps in place to successfully implement the ESC care approach.

6.3 ESC Implementation Optimization

The ESC faculty and ESC training team will host meetings that will support sites in the current ESC phase and prepare sites for the next ESC phase. These meetings are described below.

Pre-implementation Site Meeting. Meeting with individual sites and study PIs to discuss questions/concerns about training and implementation.

Group Coaching Sessions. Coaching session with all site champions in a block and ESC faculty. Occurs approximately once every 4 weeks until the Transition Period ends. These sessions will cover common problems and solutions for site training, remediation, and ESC care approach implementation.

Post-ESC Intervention Period Meeting. Group meeting with all site champions in a block and ESC faculty to ask questions/discuss problems about ESC implementation. Occurs once approximately 3-4 weeks after site enters first ESC Intervention Period.

Site Coaching Sessions. Will occur as needed with individual sites and the site's ESC faculty coach, which the DCOC will assign during the 3-day virtual site champion training.

ESC Intervention Education Meetings. Once sites move into the first ESC Intervention Period, site champions and others needed to optimize ESC implementation at the site should attend monthly educational meetings. Listed below are sample discussion topics for these meetings.

1. Providing Comprehensive & Compassionate Care for Opioid-Exposed Newborns and their Families through Prenatal Education & Outreach
2. Short-term and Long-term Effects of Ploy-substance Exposure and Caring for Newborns when Moms are Actively Using Substances
3. Benefits of Parental Presence/Rooming-In and When Need for Safe Sleep and Parent/Caregiver Self-Care Conflicts with Goals for Rooming-in
4. Benefits of Breastfeeding for Opioid-exposed Newborn & Brainstorming (Breast) Feeding Challenges
5. When Staff and Parents Conflict on Infant Care Recommendations
6. Planning for Safe & Successful Transitions to Home

6.3.1 *Sample Meeting Schedule*

Monday	Tuesday	Wednesday	Thursday	Friday
01	02	03	04	05
Block 4: Pre-implementation site meeting-->				
08	09	10<br>Block 2: Group Coaching Session	11	12
15	16	17	18	19
	Block 4: 3-Day Virtual Site Champion Training			
22	23	24<br>Block 1: ESC Intervention Education Meeting	25	26
29	30	31<br>Block 2: Post ESC Intervention Pd. Call (last individual call)	01	02

7. MAINTENANCE OF FIDELITY

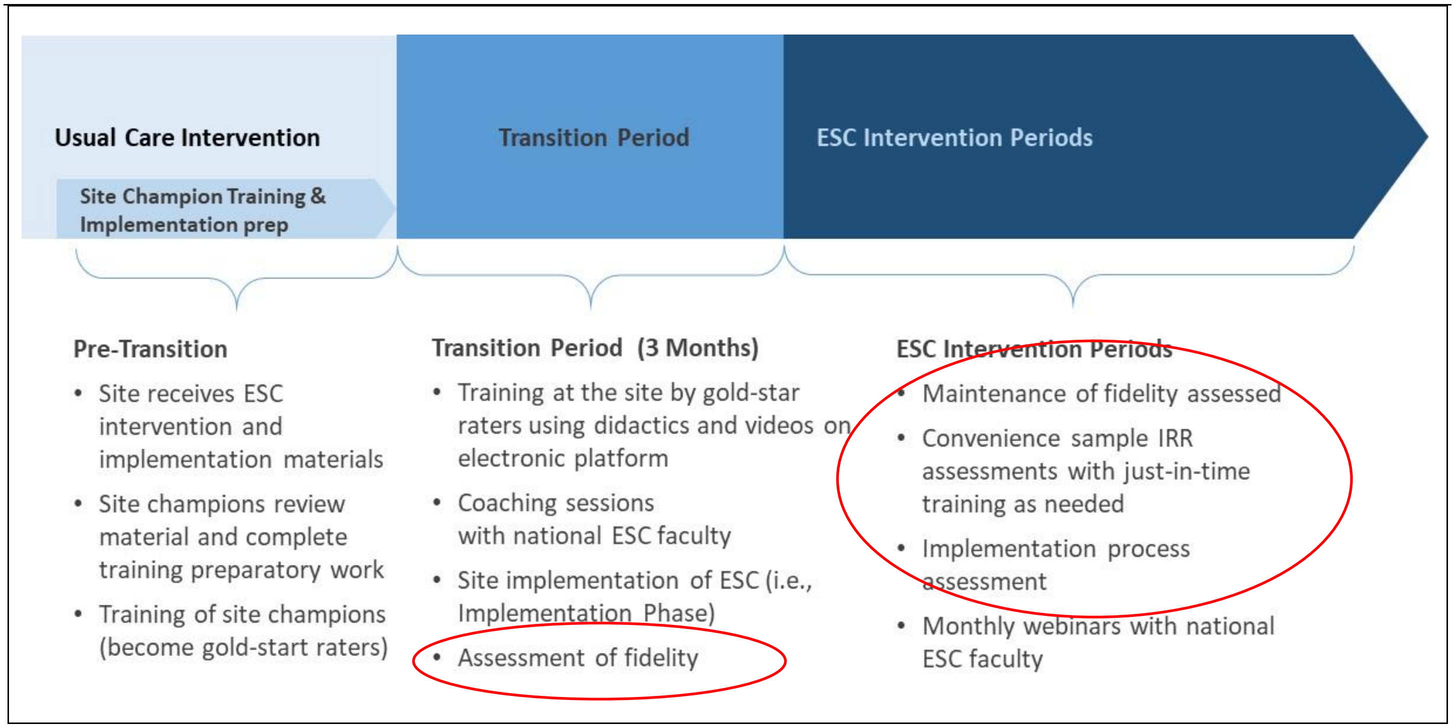

7.1 ESC Inter-rater Reliability (IRR)

7.1.1 During Implementation Phase of Transition Period

If there are more than 4 weeks between a site clinically implementing the ESC care approach and the first ESC Intervention Period, the site will evaluate 3 gold-star raters and 10 nurses, using the IRR tool with Written Case 5 or Video Case 2 in REDCap (see Appendix I for instructions). Each selected gold-star rater and nurse will complete 1 IRR assessment.

If a gold-star rater does not maintain 100% reliability or if nurses do not maintain ≥80% reliability, he or she will utilize just-in-time training (see section 5.4.8). When staffing allows, care team members who have consistently scored less than 80% reliability should not be assigned to care for infants with NOWS until improved reliability is demonstrated through the just-in-time training process.

Each site will report completion of the required IRR assessments during the transition period (if clinical implementation occurs greater than 4 weeks from the first ESC Intervention Period) to their study manager. If a site does not complete the required IRR assessments, the site must submit a protocol deviation form and the site will make a note to file on why they were unable to meet this requirement.

7.1.2 During ESC Intervention Periods

During the ESC intervention periods, sites will evaluate 3 gold-star raters and 10 nurses. Each selected gold-star rater and nurse will complete 1 IRR assessment. Sites may complete the IRR assessment with Written Case 5 or Video Case 2. For each assessment round during each period, sites will attempt to include gold-star raters and nurses who have not previously completed a reliability assessment. If a site does not complete the required IRR assessments, the site must submit a protocol deviation form and the site will make a note to file on why they were unable to meet this requirement. The protocol study team anticipates that each gold-star rater and nurse will maintain 100% and ≥80% reliability in scoring, respectively. If gold-star raters or nurses fail to maintain 100% or ≥80% reliability, the site will utilize just-in-time training (section 5.4.8) until these gold-star raters and nurses achieve 100% and ≥80% reliability, respectively. See Appendix I for instructions.

7.2 ESC Implementation Process Evaluation

7.2.1 During ESC Intervention Periods

Once a site is cleared to implement the ESC care approach **and** enters into the ESC Intervention Periods, the site will evaluate 1 nurse per ESC Intervention Period, using the ESC Implementation Process Evaluation Form in Appendix J. Sites will complete the evaluation with an infant and caregiver. Sites will complete this form on paper and retain at the site. Coordinators and PIs at each site will report the completion of the Implementation and Process Evaluation form once during each study period to their study manager. Communication between coordinators and gold-star raters at each site will be key to ensure the implementation of the ESC Care Approach is evaluated.

8. ENTERING SITE-LEVEL DEVIATIONS IN REDCAP

To enter a site-level protocol deviation, such as IRR and implementation process deviations, please follow the steps below.

1. Log into ACT NOW REDCap and click on "My projects" on the top of the page.

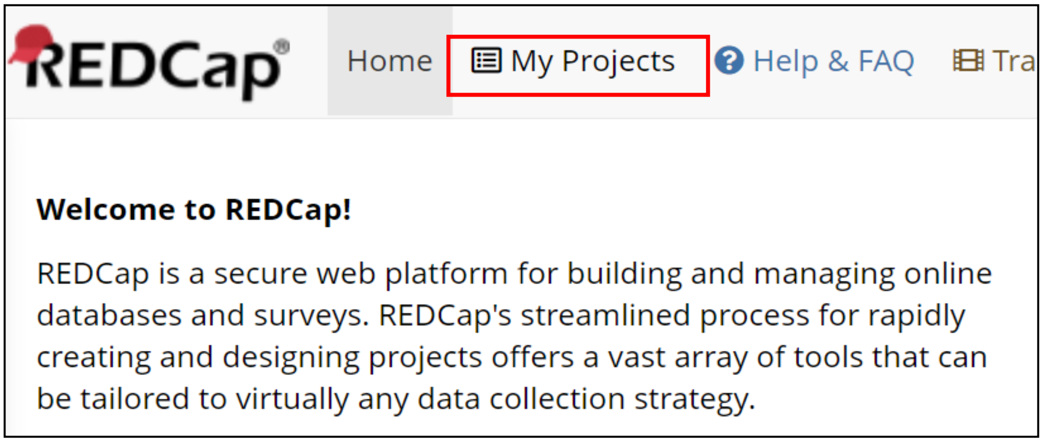

Figure 8.1 My Projects tab

2. Select the appropriate project – "ACT NOW ESC Site-Level Deviations(Only for sites currently implementing ESC)." Note that all users with access to the ACT NOW ESC Production project should also have access to this project.

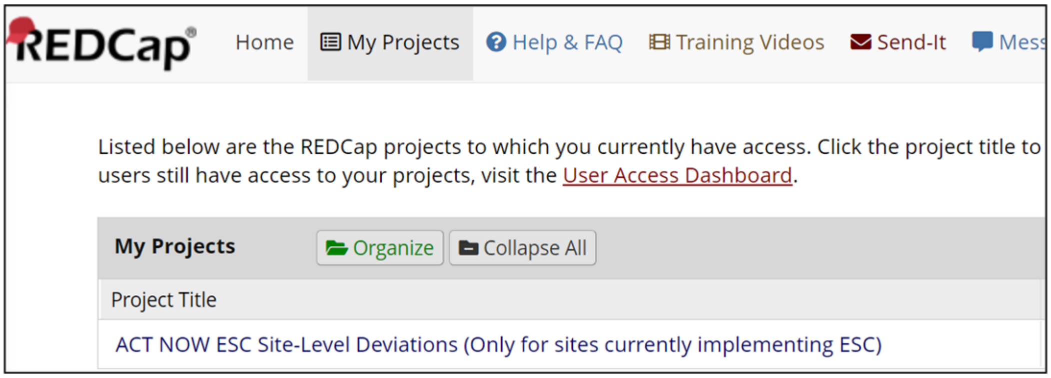

Figure 8.2 Project for ESC Site-Level Deviations

3. To enter a site-level deviation, select "Add/Edit Records" from the menu bar on the left-hand side.

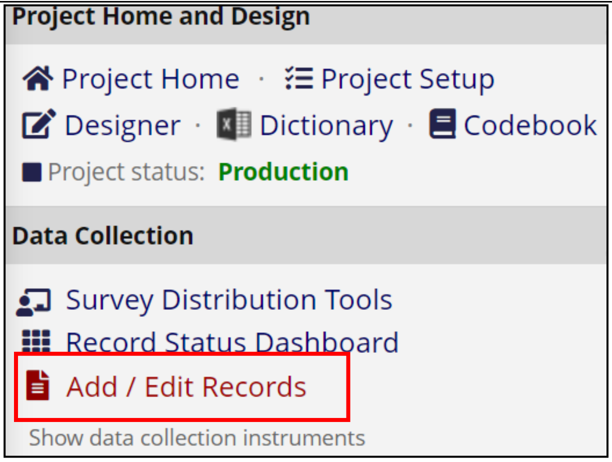

Figure 8.3 Add/Edit Records

4. Click on the green button "Add new record." The system will auto-generate a record ID sequentially for your site and will take you directly to the "Site Deviation Form."

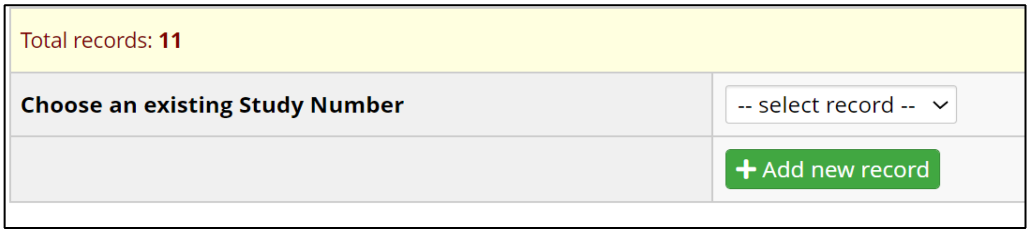

Figure 8.4 Creating a new record

5. Enter all information pertaining to the deviations into sections A, B, and C. Make sure to enter answers for all required questions.

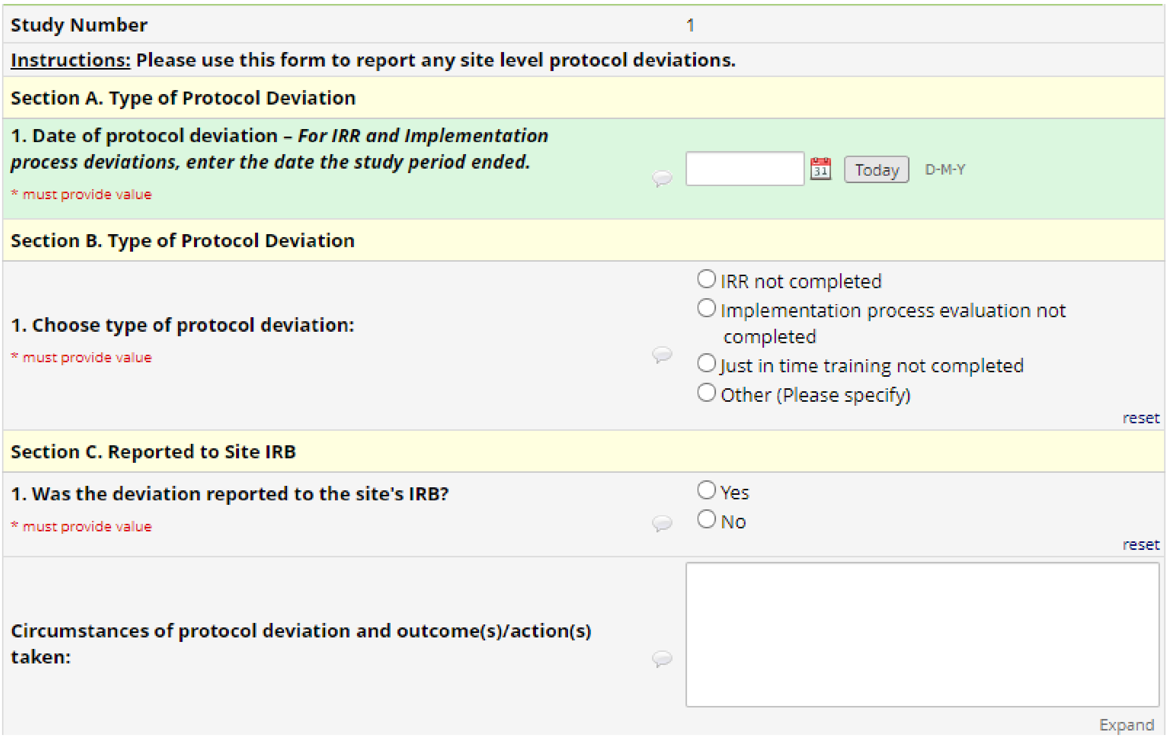

Figure 8.5 Data entry in the form

5. Once the form has been completed, select “Complete” and click “Save & Exit Form.”

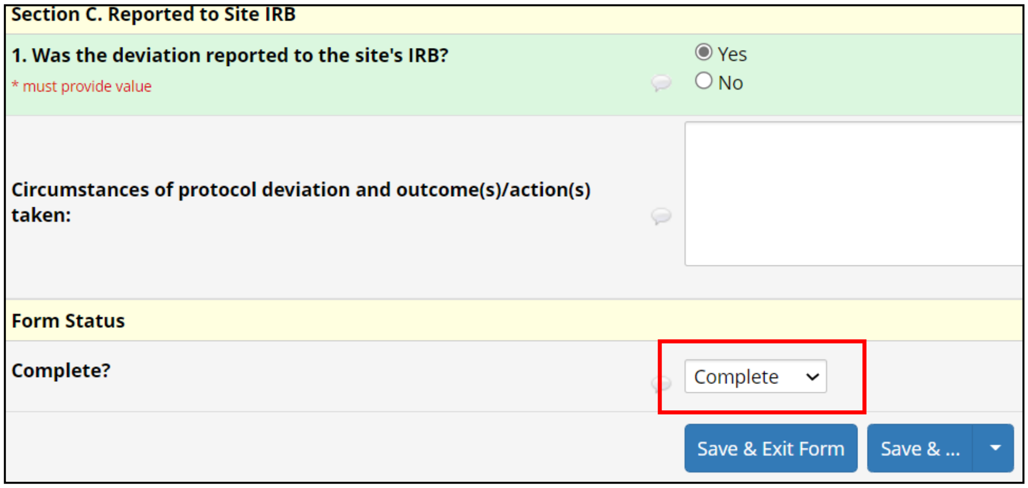

Figure 8.6 Form completion

APPENDIX A: ESC CARE TOOL AND DEFINITIONS

NIH HEAL INITIATIVE | BOSTON MEDICAL | YALE-NEW HAVEN CHILDREN'S HOSPITAL | CHaD Children's Hospital at Dartmouth-Hitchcock

EAT, SLEEP, CONSOLE (ESC) CARE TOOL ESC 3rd edition 1.30.20

- Review ESC behaviors, signs of withdrawal present, and Non-Pharm Care Interventions (NPIs) with parent(s)/caregiver every 2-4 **hours** (using **Newborn Care Diary**), ***clustering care with infant's wakings and feedings***. With each assessment, **reinforce NPIs** that parents/caregivers are implementing well (**"R"**), *and* **educate ("E") / coach parents** in ways that **other NPIs can be increased further ("I")**.
- If Yes for any ESC item *or* 3 for Consoling Support Needed: Perform a **Formal Parent/Caregiver Huddle** to *formally* **review NPIs that can be optimized further to help with infant's current ESC difficulties and continue to monitor infant closely.**

If 2nd Yes in a row for any single ESC item (*or* 2nd "3" for Consoling Support Needed) despite maximal non-pharm care *OR other significant concerns* are present (*e.g., seizures, apnea*): Perform a **Full Care Team Huddle** with **parent/caregiver, infant RN** *and* **physician or associate provider** to 1) **consider all potential etiologies** for symptoms, 2) **re-assess if NPIs are maximized** to fullest extent possible in infant's clinical setting, and 3) **determine if Neonatal Opioid Withdrawal Syndrome (NOWS)/Neonatal Abstinence Syndrome (NAS) medication treatment is needed. Continue** to **maximize all NPIs and monitor infant closely.**

Perform assessment of ESC behaviors, signs of withdrawal, and NPIs for time period since last ESC assessment – note date/time:	
**NOWS/NAS RISK ASSESSMENT**	
**Are signs of withdrawal present?** (e.g., hyperactive Moro, tremors/jitteriness, increased tone, excessive/disorganized suck) Yes / No	
**If Yes, is timing of withdrawal consistent with known opioid exposure?** Yes / No / Unsure	
**Are co-exposures present that may be contributing to signs of withdrawal?** Yes / No / Unsure (please list co-exposures)	
**Are NPIs maximized to fullest extent possible in infant's clinical setting?** Yes / No / Unsure	
**EATING**	
**Takes > 10 min to coordinate feeding** *or* **breastfeeds < 10 min** *or* **feeds < 10 mL** (*or* other age-appropriate duration/volume) **due to NOWS/NAS?** Yes / No	
**SLEEPING**	
**Sleeps < 1 hr due to NOWS/NAS?** Yes / No	
**CONSOLING**	
**Takes > 10 min to console** (*or* **cannot stay consoled for** *at least* **10 min) due to NOWS/NAS?** Yes / No	
**Consoling Support Needed**<br>1: Able to console on own<br>2: Able to console within (and stay consoled for) 10 min with caregiver support<br>3: Takes > 10 min to console (*or* cannot stay consoled for *at least* 10 min) despite caregiver's best efforts	
**CARE PLAN**	
**Formal Parent/Caregiver Huddle Performed** to formally review NPIs to be increased further? Yes / No	
**Full Care Team Huddle Performed** to formally consider all possible etiologies for symptoms, re-assess if NPIs are maximized to fullest extent possible, and determine if NOWS/NAS medication treatment is needed? Yes / No	
**Management Decision**<br>a: **Continue/Optimize NPIs**<br>b: **Initiate NOWS/NAS Medication Treatment** (e.g., if baby's symptoms & timing of symptoms are consistent with mother's particular opioid *and* NPIs are maximized to fullest extent possible in infant's clinical setting, *OR* other significant NOWS/NAS concerns are present (e.g., seizures, apnea)) – please list medication(s) initiated<br>c: **Continue NOWS/NAS Medication Treatment**<br>d: **Other** (please describe – e.g., Start 2nd Pharmacologic Agent (indicate name); Wean or Discontinue Medication Treatment)	
**PARENT/CAREGIVER PRESENCE SINCE LAST ASSESSMENT**	
**> 3 hours** (includes if parent/caregiver present entire time), **2-3 hours, 1-2 hours, < 1 hour, 0 hours** (no parent/caregiver present)	
**NON-PHARM CARE INTERVENTIONS (I = Increase Now, R = Reinforce, E = Educate for Future, NA = Not Applicable/Available)**	
**Rooming-in** (i.e., caring for infant in their own room with earlier caregiver response to infant stress or hunger cues)	
**Parent/caregiver presence** to help calm and care for infant	
**Skin-to-skin contact** when caregiver fully awake/alert to help organize infant feeding behaviors, calming & sleep	
**Holding by parent/caregiver/cuddler** to help calm infant & aid in sleep (with caregiver fully awake/alert)	
**Safe & effective swaddling** (e.g., extremities swaddled in flexed position, blanket snug, no extra blanket around baby's face)	
**Optimal feeding** (e.g., baby offered feedings when showing hunger cues & fed till content)	
**Non-nutritive sucking** with infant's hand, pacifier, adult caregiver's washed or gloved finger	
**Quiet, low light environment** to help limit overstimulation of infant (e.g., tv volume down, quiet "white noise" machine or phone app)	
**Rhythmic movement** provided by parent/caregiver or infant calming device (e.g., "jiggling" or infant swing in presence of alert adult)	
**Additional help/support in room** (e.g., other parent, family member, friend, cuddler, staff member, recovery coach, DCYF worker)	
**Limiting # of visitors & duration of visit(s)** to minimize disruptions in infant's care environment & sleep	
**Clustering care** & assessments with infant's awake times (e.g., RN & infant provider perform assessment together after infant feedings)	
**Safe sleep/fall prevention** (e.g., infant sleeps on back, safely swaddled, in own sleep space)	
**Parent/caregiver self-care & rest** (e.g., identifying another adult to care for infant so parent can rest or take a walk/break)	
**Optional Comments:** (e.g., staff caring for/consoling baby as parents not available or able to safely care for baby)	

**Special note: Numbers above are not intended as a "score" but instead may indicate/identify a need for increased intervention*

ESC Care Tool 3rd edition 1.30.20

© 2017 Boston Medical Center Corporation, Dr. Matthew Grossman and Children's Hospital at Dartmouth-Hitchcock

DEFINITIONS

EATING

- **Takes > 10 min to coordinate feeding *or* breastfeeds < 10 min *or* feeds < 10 mL** (*or* other age-appropriate duration/volume) **due to NOWS/NAS?:** Baby unable to coordinate feeding *within* 10 minutes of showing hunger *OR* sustain feeding for *at least* 10 minutes at breast *OR* with 10 mL by alternate feeding method (*or other age-appropriate duration/volume)* due to opioid withdrawal symptoms (e.g., fussiness, tremors, uncoordinated suck, excessive rooting).
- ***Special Note:*** **Do not indicate Yes** if poor eating is clearly **due to non-opioid related factors** (e.g., prematurity, transitional sleepiness or spittiness in first 24 hours, inability to latch due to infant/maternal anatomical factors).

SLEEPING

- **Sleeps < 1 hour due to NOWS/NAS:** Baby unable to sleep for *at least* one hour, after feeding well, due to opioid withdrawal symptoms (e.g., fussiness, restlessness, increased startle, tremors).
- ***Special Note:*** **Do not indicate Yes** if sleep < 1 hour is clearly **due to non-opioid related factors** (e.g., symptoms in first day likely due to nicotine or SSRI withdrawal, physiologic cluster feeding in first few days of life, interruptions in sleep for routine newborn testing).

CONSOLING

- **Takes > 10 min to console** (*or* **cannot stay consoled for *at least* 10 min) due to NOWS/NAS:** Baby takes longer than 10 minutes to console *OR* cannot stay consoled for *at least* 10 minutes (due to opioid withdrawal symptoms) despite infant caregiver/provider's best efforts to implement NPIs (e.g., skin-to-skin contact, non-nutritive sucking when baby not hungry).
- ***Special Note:*** **Do not indicate Yes** if infant's difficulties consoling are clearly **due to non-opioid related factors** (e.g., caregiver non-responsiveness to infant hunger cues, circumcision pain).

CONSOLING SUPPORT NEEDED

1. **Able to console on own:** Able to console on own without any caregiver support needed.
2. **Able to console within (and stay consoled for) 10 min with caregiver support:** Baby with absence of crying, grimacing, or other signs of distress while being held (or otherwise consoled) by a caregiver.
3. **Takes > 10 min to console** (*or* **cannot stay consoled for *at least* 10 min) despite caregiver's best efforts:** Baby with presence of crying, grimacing, squirming/tensing, or other signs of distress despite a caregiver's best efforts to implement recommended NPIs (e.g., parent/caregiver presence, skin-to-skin, holding, safe swaddling, optimal feeding, non-nutritive sucking when not hungry).

CARE PLAN

- **Formal Parent/Caregiver Huddle:** RN bedside huddle with parent/caregiver to *formally* review NPIs that can be optimized ("Increased") further to help with infant's current eating, sleeping, and/or consoling difficulties. To be performed if infant receives Yes for any ESC item *or* 3 for Consoling Support Needed.
- **Full Care Team Huddle:** Formal huddle with parent/caregiver, infant RN *and* physician or associate provider to 1) consider all potential etiologies for symptoms, 2) re-assess if NPIs are maximized to fullest extent possible in infant's clinical setting, and 3) determine if NOWS medication treatment is needed. To be performed if infant receives 2nd Yes in a row for any single ESC item (*or* 2nd "3" for Consoling Support Needed) despite maximal non-pharm care *OR other significant concerns* are present.

PARENT/CAREGIVER PRESENCE SINCE LAST ASSESSMENT:

Time (in hours) since last assessment that parent (or other caregiver) spent together with infant in own room *or* in Nursery.

OPTIMAL FEEDING:

- **Baby feeding at early hunger cues and until content** without any limit placed on duration or volume of feeding. Feedings are encouraged **at least every 3 hours, optimally 8-12 times per day**, to ensure baby does not become too hungry or disorganized with feeding and to optimize nutritional intake. A baby may remain sleeping for more than 3 hours *for therapeutic rest* if feeding difficulties or excessive weight loss are *not* present. If a pacifier is used, it should be introduced only after a baby has been fed or offered a feeding. As infants with NOWS/NAS may have poor feeding, have excessive/watery stools, or be hypermetabolic, closely follow daily weights and provide **increased volume and/or caloric density of feedings**, as needed, **for more than expected weight loss *and/or* poor weight gain for age.**
- **Breastfeeding:** Baby latching deeply with comfortable latch for mother, and sustained active suckling for baby with only brief pauses noted. **If feeding difficulties present:** a) **assist directly with breastfeeding** to help achieve more optimal latch and position, b) **demonstrate hand expression** and **have mother express colostrum prior to and/or during feedings**, and/or c) have baby feed on a clean or gloved adult **finger first to organize suck prior to latching**. As able based on infant's symptoms, consider withholding pacifiers until babies are breastfeeding well due to the potential to interfere with a good latch/suck. Consider use of nipple shield to facilitate palatal stimulation, or supplementation at the breast (as tolerated by mother), if infant requires assistance to maintain latch/suck.
- **Bottle feeding:** Baby effectively coordinating suck and swallow without gagging or excessive spitting up. **If feeding difficulties are present:** a) **assess need for altered nipple shape/flow rate**, b) instruct parent to **provide chin support during feedings**, *and/or* c) **modify position of bottle and flow of milk** to assist baby with feeding (e.g., modified side-lying position).
- **Consult a feeding specialist** (e.g., lactation, speech therapy, feeding team) **when feeding difficulties are present.**

ESC Care Tool 3rd edition 1.30.20

© 2017 Boston Medical Center Corporation, Dr. Matthew Grossman and Children's Hospital at Dartmouth-Hitchcock

APPENDIX B: ESC IRR TOOL

NIH HEAL INITIATIVE   BOSTON MEDICAL CENTER   YALE-NEW HAVEN CHILDREN'S HOSPITAL   CHaD Children's Hospital at Dartmouth-Hitchcock

EAT, SLEEP, CONSOLE (ESC) Inter-rater Reliability Tool

ESC 3rd edition 1.30.20

- **Review ESC behaviors, signs of withdrawal present,** and **Non-Pharm Care Interventions (NPIs)** with parent(s)/caregiver **every 2-4 hours** (using **Newborn Care Diary**), ***clustering care with infant's wakings and feedings.*** With each assessment, **reinforce NPIs** that parents/caregivers are implementing well **("R"),** *and* **educate ("E") / coach parents** in ways that **other NPIs can be increased further** (**"I"**).
- If **Yes** for any **ESC** item *or* **3** for **Consoling Support Needed:** Perform a **Formal Parent/Caregiver Huddle** to ***formally*** **review NPIs that can be optimized further to help with infant's current ESC difficulties** and **continue to monitor infant closely.**
- If **2nd Yes in a row** for **any single ESC** item **(*or* 2nd "3"** for **Consoling Support Needed)** despite **maximal non-pharm care** *OR* ***other significant concerns*** are present *(e.g., seizures, apnea)*: Perform a **Full Care Team Huddle** with **parent/caregiver, infant RN** *and* **physician or associate provider** to 1) **consider all potential etiologies** for symptoms, 2) **re-assess if NPIs** are **maximized** to fullest extent possible in infant's clinical setting, and 3) **determine if Neonatal Opioid Withdrawal Syndrome (NOWS)/Neonatal Abstinence Syndrome (NAS) medication treatment is needed. Continue** to **maximize all NPIs and monitor infant closely.**

Perform assessment of ESC behaviors for time period since last ESC assessment *– note date/time:*	**RN**	**Gold-Star Rater**
**EATING**		
**Takes > 10 min to coordinate feeding** *or* **breastfeeds < 10 min** *or* **feeds < 10 mL** (*or* other age-appropriate duration/volume) **due to NOWS/NAS?** Yes / No		
**SLEEPING**		
**Sleeps < 1 hr due to NOWS/NAS?** Yes / No		
**CONSOLING**		
**Takes > 10 min to console (*or* cannot stay consoled for *at least* 10 min) due to NOWS/NAS?** Yes/No		
**Consoling Support Needed**<br>**1:** Able to console on own<br>**2:** Able to console within (and stay consoled for) 10 min with caregiver support<br>**3:** Takes > 10 min to console (*or* cannot stay consoled for *at least* 10 min) despite caregiver's best efforts		
**CARE PLAN**		
**Formal Parent/Caregiver Huddle Performed** to formally review NPIs to be increased further? Yes / No		
**Full Care Team Huddle Performed** to formally consider all possible etiologies for symptoms, re-assess if NPIs are maximized to fullest extent possible, and determine if NOWS/NAS medication treatment is needed? Yes / No		
**INTER-RATER RELIABILITY PERCENTAGE**	**%**	

****Special note:*** *Numbers above are* ***not*** *intended as a "score" but instead may indicate identify a need for increased intervention.*

Determining Inter-rater Reliability Percentage: Calculate the percent agreement between the RN and the Gold Star rater on the 6 areas highlighted in yellow above. For example, if 6 out of 6 items are in agreement = 100% reliability, and if 5 out of 6 items are in agreement = 83% reliability.

© 2017 Boston Medical Center Corporation, Dr. Matthew Grossman and Children's Hospital at Dartmouth-Hitchcock

APPENDIX C: CONVERSION OF PHARMACOLOGICAL TREATMENT ALGORITHMS

Appendix C provides examples of algorithm conversions, from Finnegan-based pharmacologic treatment algorithms (left) to ESC-based algorithms (right). These are examples ONLY. Once your site has converted your pharmacologic treatment algorithm for use with ESC, please submit your algorithm to Leslie Young, Kathy Edwards and DeAnn Hubberd for general review. If your site needs assistance converting your treatment algorithm, please email Leslie Young, Kathy Edwards, or DeAnn Hubberd

Morphine

Finnegan-based algorithm (left)

Initiate oral morphine if Finnegan scores 3 ≥8 or 2 ≥12* and non-pharmacologic care has been implemented

Non-pharmacologic care includes: Breastfeeding (if no medical contraindication), holding and swaddling, encouraging parental involvement in care (which includes skin-to-skin), environmental controls (low light, and quiet environment), support with momma-roos and adlib feeding (goal is feeding on demand)

* Scoring should be done after feeding

Morphine Initiation

Starting dose of enteral morphine solution (0.5mg/ml)
- 0.05mg/kg/dose po/ng Q3 hours (birth weight is used for dosing)

Morphine Escalation

If Finnegan Score remain elevated: 3 scores ≥8 or 2 scores ≥12:
AND
- Caregivers are using non-pharmacologic care
- Non-NOWS causes of symptoms have been ruled out

Increase baseline dose by 0.02mg/kg/dose
- Example: If baseline dose is 0.05mg/kg/dose po/ng Q3 hours then new dose will be 0.07mg/kg/dose po/ng Q3

Continue to escalate if Finnegan scores remain 3 scores ≥8 or 2 scores ≥12 until the infant is stabilized and has no more than 2 scores greater than 8 in a 24 hours period

Maximum recommended dose 0.2 mg/kg/dose Q3

Secondary Medication

Add clonidine 1mcg/kg/dose Q3 if Finnegan Score remain elevated: 3 scores ≥8 or 2 scores ≥12
AND:
- Caregivers are using non-pharmacologic care
- Non-NOWS causes have been ruled out

AND
- Morphine dose has reached 50% of maximum dose

OR
- Unable to wean by day 7 of treatment

Escalate by 0.5 mcg/kg/dose if 3 scores ≥8 or 2 scores ≥12

Maximum - 2 mcg/kg/dose

Wean per Clonidine wean block

Morphine Weaning

Once the infant is stabilized and has no more than 2 scores >8 in a 24 hours period start weaning
- For the initial wean – wean by 10% of the maximum total dose and continue Q3 hour dosing
- If the initial wean is tolerated and the infant has no more than 2 scores > 8 for 24 hours then continue to wean 10-15% of total daily dose
- Discontinue morphine when dose is 0.01mg/kg/dose or not longer possible to measure for infants less than 2.5kg

Failed Weaning

If 2 Finnegan Scores >12 after a wean or after discontinuation of morphine
- Maximize clonidine if already receiving clonidine

OR
- Provide up to 2 rescue doses of morphine at last effective dose.
- Score remain elevate as above, after rescue doses restart the last effective dose of morphine for a minimum of 24 hours and reassess

Discharge Home

Observe off all medications and D/C home when no more than 2 scores are >8 for at least 48 hours
- Consider the following prior to D/C
  - Can the infant be maintained in recommended safe sleep position when sleeping
  - Primary caregiver has demonstrated the ability to provide all required care

ESC-based algorithm (right)

Initiate oral morphine after a full care team huddle if infant with ≥ 1 "yes" response to ESC: 1) E = poor feeding due to NAS, 2) S = sleep < 1 hour due to NAS, OR 3) C = unable to console within 10 minutes and stay consoled for 10 minutes and/or 3s for soothing support AND:
- Non pharmacologic care has been optimized first
- Evaluated for non-NAS causes of symptoms

Morphine Initiation

Starting dose of enteral morphine solution (0.5mg/ml)
- 0.05mg/kg/dose po/ng Q3 hours (birth weight is used for dosing)

Morphine Escalation

Escalate oral morphine after a team huddle if infant with ≥ 1 "yes" response to ESC: 1) E = poor feeding due to NAS, 2) S = sleep < 1 hour due to NAS, OR 3) C = unable to console within 10 minutes and stay consoled for 10 minutes and/or 3s for soothing support AND:
- Non pharmacologic care has been optimized first
- Evaluated for non-NAS causes of symptoms

Increase baseline dose by 0.02mg/kg/dose
- Example: If baseline dose is 0.05mg/kg/dose po/ng Q3 hours then new dose will be 0.07mg/kg/dose po/ng Q3

Continue to escalate per ESC assessment until infant is stabilized and has predominantly "no responses" to ESC

Maximum recommended dose 0.2 mg/kg/dose Q3

Secondary Medication

Add clonidine 1mcg/kg/dose Q3 after a team huddle for ≥ 1 "yes" response to ESC and/or 3s for soothing support AND:
- Non pharmacologic care has been optimized first
- Evaluated for non-NAS causes of symptoms

AND
- Morphine dose has reached 50% of maximum dose

OR
- Unable to wean by day 7 of treatment

Escalate by 0.5 mcg/kg/dose for 1 yes response to ESC or 3s for soothing support if nonpharmacologic care continues to be maximized

Maximum - 2 mcg/kg/dose

Wean per Clonidine Wean block

Morphine Weaning

Once the infant is stabilized and has predominantly "no" responses to ESC for 24 hours then begin weaning
- For the initial weaning dose wean by 10% of the maximum total dose and continue Q3 hour dosing
- If the initial wean is tolerated and the infant has predominantly "no" responses to ESC for 24 hours then continue to wean 10-15% of total daily dose
- Discontinue morphine when dose is 0.01 mg/kg/dose or no longer possible to measure for infants less than 2.5kg

Failed Weaning

If an infant increases to persistently "yes" responses to ESC after a wean or after discontinuation of morphine and non-pharmacologic care remains maximized.
- Maximize clonidine if already receiving clonidine

OR
- Provide up to 2 rescue doses of morphine at last effective dose
- Score remain elevate as above, after rescue doses restart the last effective dose of morphine for a minimum of 24 hours and reassess

Discharge Home

Observe off all medications and D/C home when no responses to ESC for 24 hours or more.
- Consider the following prior to D/C
  - Can the infant be maintained in recommended safe sleep position when sleeping?
  - Is the intensity of non-pharmacologic care easily replicated at home?
    - Infants that must be held continuously are not ready for discharge

Example
Example

**Clonidine Wean**<br>• Infant is off morphine and no more than 2 scores are >8 in the preceding 24 hour period wean clonidine by 0.5 mcg/kg/dose every 24 hours until dose of 1mcg/kg/dose Q3 is reached<br>THEN<br>• If no more than 2 scores are > 8 in the preceding 24 hour period then wean to 1 mcg/kg/Q6<br>THEN<br>• If no more than 2 scores are > 8 in the preceding 24 hour period then d/c clonidine and refer to **Discharge Home Block** on the algorithm<br><br>Monitor BP after each wean and if resting systolic BP consistently >110 consider increasing the dose until BP improve and consider weaning every 48 hours rather than every 24 hours<br><br>**For Infants with signs of withdrawal without a history of opioid exposure please start clonidine and not morphine if pharmacologic treatment is deemed necessary**	**Clonidine Wean**<br>• Infant is off morphine and has predominantly "no" responses in the preceding 24 hour period wean clonidine by 0.5mcg/kg/dose every 24 hours until dose of 1mcg/kg/dose Q3 is reached<br>THEN<br>• If the infant continues to have predominantly "no" responses in the preceding 24 hour period then wean to 1 mcg/kg/Q6<br>THEN<br>• If the infant continues to have predominantly "no" responses in the preceding 24 hour period then d/c clonidine and refer to **Discharge Home Block** on the algorithm<br><br>Monitor BP after each wean and if resting systolic BP consistently >110 consider increasing the dose until BP improve and consider weaning every 48 hours rather than every 24 hours

Methadone

Finnegan Methadone Algorithm

- **Initiation:** start pharmacologic treatment for infants with 3 consecutive Finnegan scores ≥ 8 or 2 consecutive Finnegan scores ≥ 12

	Methadone Dose	Dosing Interval	Number of Doses
Step 1	0.1 mg/kg	Q6h	4
Step 2	0.07 mg/kg	Q12h	2
Step 3	0.05 mg/kg	Q12h	2
Step 4	0.04 mg/kg	Q12h	2
Step 5	0.03 mg/kg	Q12h	2
Step 6	0.02 mg/kg	Q12h	2
Step 7	0.01 mg/kg	Q12h	2
Step 8	0.01 mg/kg	Q24h	1

- **Escalation**
  - If infant fails step 1 (scores >12) consider steps 1A through 1C

	Methadone Dose	Dosing Interval	Number of Doses
Step 1a	0.1 mg/kg	Q4h	6
Step 1b	0.1 mg/kg	Q8h	3
Step 1c	0.1 mg/kg	Q12h	2

  - Consider adding phenobarbital if unable to wean for 2 consecutive days
    - Loading dose 20 mg/kg PO
    - Maintenance dose 5 mg/kg PO daily
- **Weaning**
  - Wean to next step if average Finnegan score is <8 for the past 24 hours
  - If average Finnegan score is 8-12, do not wean
  - If average Finnegan score is >12, consider an extra dose of methadone at the current step, or return to the previous step
- **Discharge**
  - Observe for 48 hours off methadone

ESC Methadone Algorithm

- **Initiation**
  - Consider initiation of medication after a full care team huddle if infant has ≥ 1 "yes" response to ESC: 1) E = poor feeding due to NAS, 2) S = sleep < 1 hour due to NAS, or 3) C = unable to console within 10 minutes AND:
    - non pharmacologic care has been optimized first
    - evaluated for non-NAS causes of symptoms
  - Consider giving one dose of methadone and evaluating baby's response before initiating the taper. If the baby continues to have ≥ 1 "yes" response to ESC after a single dose, initiate the taper
  - Initiate at step 1a for infants who have ≥ 2 "yes" responses to ESC despite optimization of non-pharmacologic care

	Methadone Dose	Dosing Interval	Number of Doses
Step 1	0.1 mg/kg	Q6h	4
Step 2	0.07 mg/kg	Q12h	2
Step 3	0.05 mg/kg	Q12h	2
Step 4	0.04 mg/kg	Q12h	2
Step 5	0.03 mg/kg	Q12h	2
Step 6	0.02 mg/kg	Q12h	2
Step 7	0.01 mg/kg	Q12h	2
Step 8	0.01 mg/kg	Q24h	1

- **Escalation**
  - If infant continues to have ≥1 "yes" response to ESC after 24 hours of step 1 and optimized non-pharmacologic care, proceed to step 1a

	Methadone Dose	Dosing Interval	Number of Doses
Step 1a	0.1 mg/kg	Q4h	6
Step 1b	0.1 mg/kg	Q8h	3
Step 1c	0.1 mg/kg	Q12h	2
Proceed to step 2			

  - Consider adding phenobarbital if unable to wean for 2 consecutive days
    - Loading dose 20 mg/kg PO
    - Maintenance dose 5 mg/kg PO daily
- **Weaning**
  - Wean to next step if infant has predominantly "no" responses to ESC
  - If infant persistently has ≥1 "yes" response to ESC for most assessments in the past 24 hours, consider holding at the current dose
- **Discharge**
  - Observe for 48 hours after discontinuing methadone and consider discharge home when "no" responses to ESC for at least 24 hours
  - Consider the following prior to discharge:
    - Can the infant be maintained in recommended safe sleep position when sleeping?
    - Is the intensity of non-pharmacologic care easily replicated at home?

Buprenorphine

FINNEGAN BUPRENORPHINE ALGORITHM

Administration

- Buprenorphine 75 mcg/ml sublingual solution
- Administer under the tongue followed by a pacifier for maximal absorption
- If dose volume > 0.5 mL administer two minutes apart in two separate aliquots

Initiation

- Initiate at Step 1 for infants with Finnegan scores ≥ 8 on 3 consecutive scorings
- If Finnegan scores are ≥ 8 after 2 doses of step 1, proceed to step 1a

Step	Dose	Frequency	Doses
1	4.5 mcg/kg	Q8h	3
1a	8 mcg/kg	Q8h	3
2	3.5 mcg/kg	Q8h	3
3	2.5 mcg/kg	Q8h	3
4	2 mcg/kg	Q8h	3
5	2 mcg/kg	Q12h	2

Escalation

- After step 1a, if infant is still having scores ≥ 8, continue increasing dose by 2 mcg/kg every 16 hours until stabilized (2 consecutive scores <8)
- Once stabilized, start to wean by 2 mcg/kg every 24 hours
- Continue to wean each day by 2 mcg/kg/dose as tolerated until back to Step 1

Weaning

- Average 24 hour Finnegan score should be <8 to wean
- Average Finnegan score 8-12, do not wean
- Average Finnegan score >12, go back one step on taper (backslide)
- If unable to wean 24 hours after backsliding, or for two days in a row, add phenobarbital
- Observation of infant should be maintained for 48 hours after discontinuing buprenorphine

Adjunct: Phenobarbital 10 mg/ml Oral Suspension

- Phenobarbital used to facilitate weaning of the primary opioid and can be continued at discharge for outpatient weaning, as tolerated.

Phenobarbital Dosing

- Loading dose: 20 mg/kg once
- Maintenance dose: 5 mg/kg/day starting 12 hours after loading dose
- May increase dose as needed to maintain effect (range: 2-8 mg/kg/day)
- Discharge: Provide one-month supply after discharge for outpatient wean

ESC BUPRENORPHINE ALGORITHM

Administration

- Buprenorphine 75 mcg/ml sublingual solution
- Administer under the tongue followed by a pacifier for maximal absorption
- If dose volume > 0.5 mL administer two minutes apart in two separate aliquots

Initiation

- Consider initiation of medication after a full care team huddle if infant with ≥1 "yes" response to ESC: 1) E = poor feeding due to NAS, 2) S = sleep <1 hour due to NAS, OR 3) C = unable to console within 10 minutes, AND:
  - Non-pharmacologic care has been optimized first
  - Evaluated for non-NAS causes of symptoms
- Consider giving one dose of medication and evaluating response before initiating taper

Step	Dose	Frequency	Doses
1	4.5 mcg/kg	Q8h	3
1a	8.5 mcg/kg	Q8h	3
2	3.5 mcg/kg	Q8h	3
3	2.5 mcg/kg	Q8h	3
4	2 mcg/kg	Q8h	3
5	2 mcg/kg	Q12h	2

Escalation

- If infant continues to have ≥1 "yes" response to ESC after 16 hours of step 1 and optimized non-pharm care, proceed to step 1a
- After step 1a, if infant continues to have ≥1 "yes" response, continue increasing dose by 2 mcg/kg every 16 hours until stabilized (predominantly "no" responses to ESC)
- Once stabilized, start to wean by 2 mcg/kg every 24 hours
- Continue to wean each day by 2 mcg/kg/dose as tolerated until back to Step 1

Weaning

- Once the infant is stabilized and has predominantly "no" responses to ESC for 24 hours, begin weaning
- If infant has persistent "yes" responses to ESC, consider holding at the current dose x 24 hours
- If an infant has predominantly "yes" responses to ESC after a wean, go back one step on the taper
- If unable to wean 24 hours after backsliding, or for two days in a row, add phenobarbital

Discharge

- Observe for 48 hours after discontinuing buprenorphine and consider discharge home when "no" responses to ESC for at least 24 hours
- Consider the following prior to discharge:
  - Can the infant be maintained in recommended safe sleep position when sleeping?
  - Is the intensity of non-pharmacologic care easily replicated at home?

Adjunct: Phenobarbital 10 mg/ml Oral Suspension

- Phenobarbital is used to facilitate weaning of the primary opioid and can be continued at discharge for outpatient weaning, as tolerated.

Phenobarbital Dosing

- Loading dose: 20 mg/kg once
- Maintenance dose: 5 mg/kg/day starting 12 hours after loading dose
- May increase dose as needed to maintain effect (range: 2-8 mg/kg/day)
- Discharge: Provide one-month supply after discharge for outpatient wean

APPENDIX D: ESC CARE TOOL EPIC TIP SHEET

ESC EPIC Flowsheet Example

Example of the ESC flowsheet from an end-user's perspective. Additional information is available on the training portal.

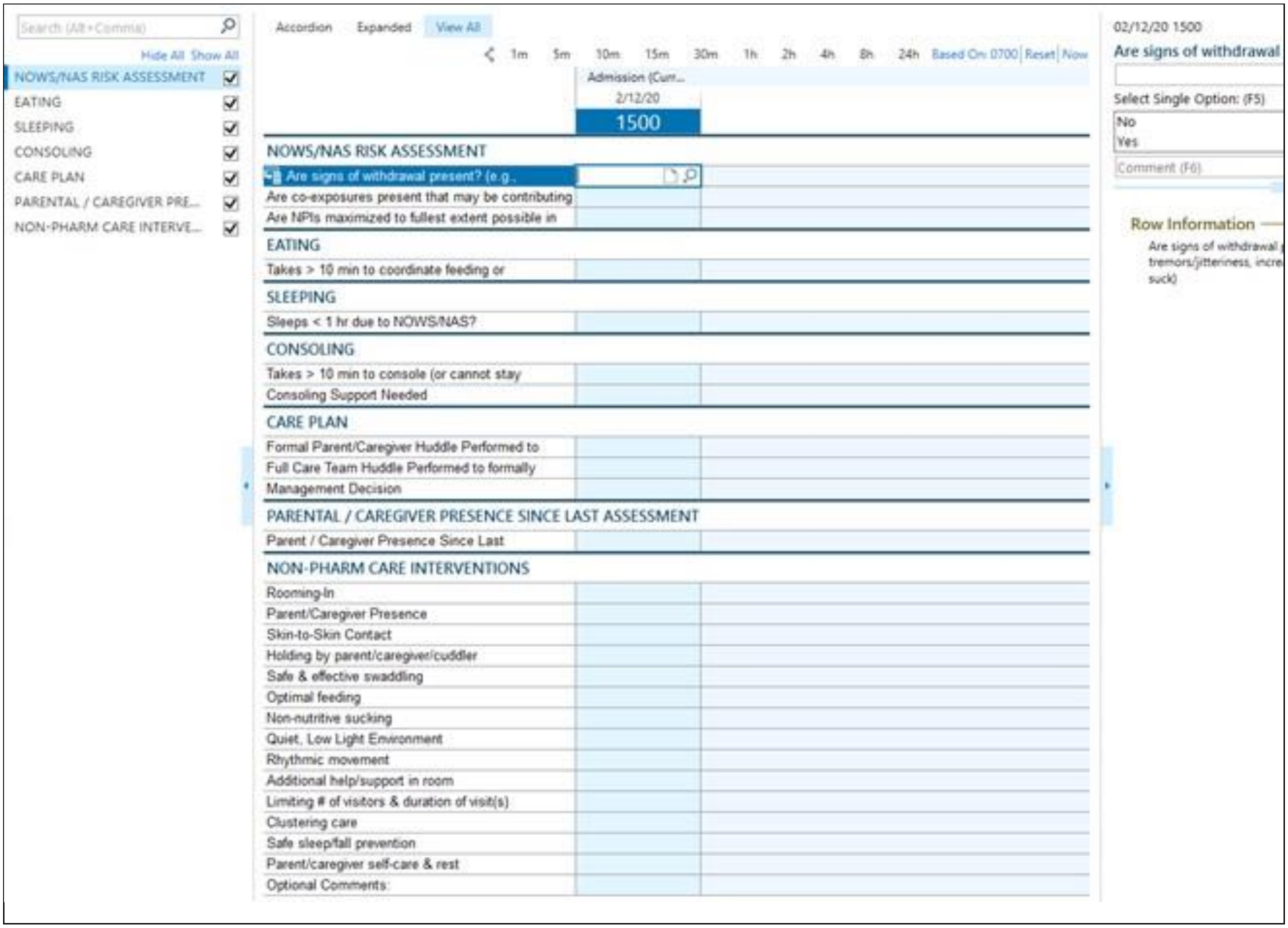

APPENDIX E: ESC CARE TOOL CERNER TIP SHEET

ESC Cerner Flowsheet Example

Example of the ESC flowsheet from an end-user's perspective. Additional information is available on the training portal.

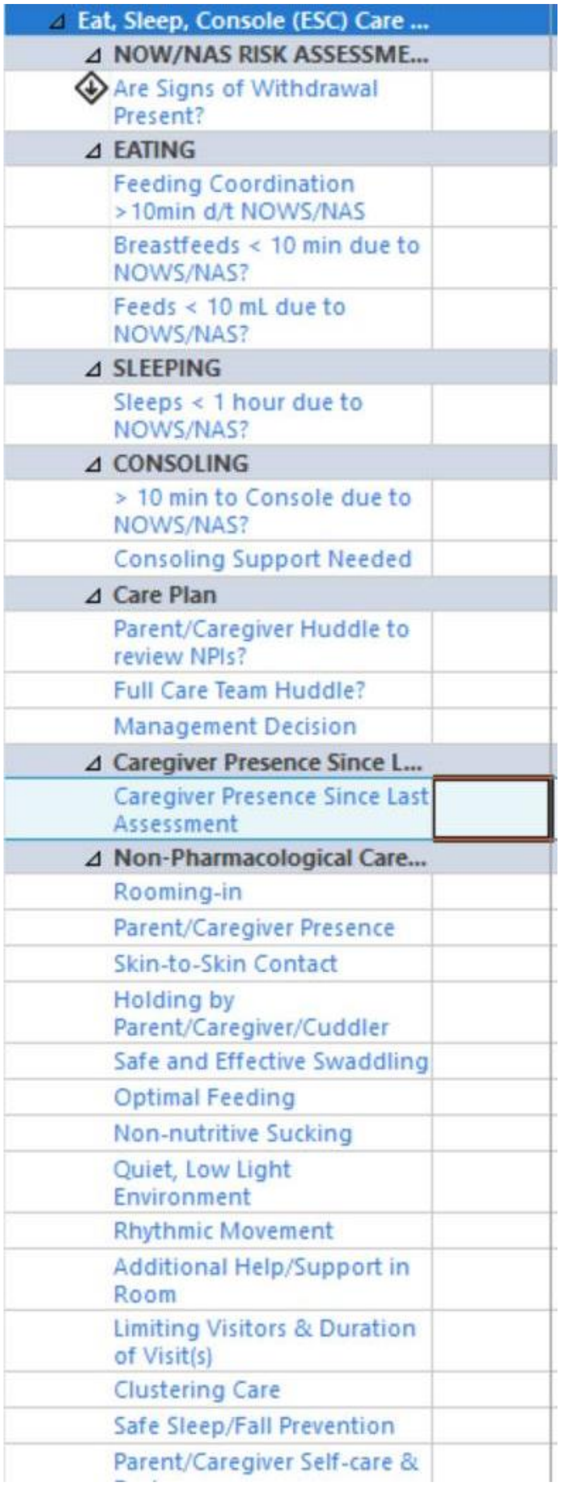

PENDIX F: PARENT EDUCATION BROCHURE

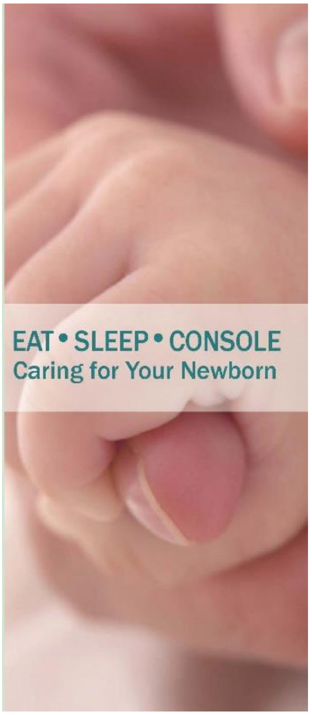

Congratulations on your pregnancy and/or the birth of your new baby!

Our team is committed to providing you and your baby with the best care possible. The information in this pamphlet will help you learn how to best care for your baby after birth.

What is NOWS?

Neonatal Opioid Withdrawal Syndrome (or NOWS), occurs when a baby withdraws from opioids after birth. This is also sometimes called Neonatal Abstinence Syndrome (or NAS). Most babies show signs of withdrawal 2 to 3 days after birth but some may not show signs until day 4 or 5.

Your baby will likely stay in the hospital until most of the symptoms of NOWS are over.

What are the most common signs of NOWS?

- Tremors, jitteriness, or shaking of arms and legs
- Tight muscles in arms and legs
- Fussiness or hard to console (calm down)
- Problems eating or sleeping
- Need to suck when not hungry
- Frequent spitting up or vomiting
- Loose or watery stools (poops)
- Losing too much or not gaining enough weight (after day 4)

Serious symptoms like stopping breathing or seizures are possible but very rare.

NOWS Assessment

We will watch your baby closely and assess for signs of withdrawal every few hours. Let your nurse know when your baby is done feeding as this is a good time to check your baby. You can also help us watch your baby by keeping track of:

- How well your baby eats.
- How well your baby sleeps.
- How well your baby consoles (calms).
- What kinds of things help your baby console or calm (your presence, skin-to-skin contact, holding, swaddling, sucking, a dark or quiet room, rhythmic movement).
- What your baby's stools (poops) are like (loose, very loose, watery).

We will give you a **Newborn Care Diary** to help you keep track of all of these things!

What will my care team do to make sure my baby is healthy?

During your baby's time in the hospital, you will be your baby's primary caregiver. We will be here to help you but your baby will do best if you are the one providing the care.

- We will monitor your baby in the hospital for several days.
- If your baby has problems with eating, sleeping, or consoling (calming), we will teach you ways to help your baby.
- If there are still problems with eating, sleeping, or consoling after all you and we have done to help your baby, we will talk with you about whether medicine may help your baby.
- Medicine may also be needed if there are other problems present, such as problems with breathing or losing too much weight.

How can I best help my baby?

Rooming-In & Parent/Caregiver Presence. Keeping your baby with you in your own room is called "Rooming-in." If available in your hospital, rooming in helps you provide a quiet and calm space and allows you to respond to your baby's needs.

Skin-to-Skin. Spend as much time as you can "skin-to-skin" with your baby when you are awake and alert. This helps your baby eat and sleep better and can also help with other symptoms of withdrawal.

Holding, Swaddling, & Cuddling. When you are awake and not doing skin-to-skin, hold your baby in your arms, either in their clothes, or swaddled in a light blanket.

Feeding. Feed your baby whenever he/she is showing hunger cues and until content, at least every 3-4 hours. It is best to breastfeed your baby if you are able to.

Sucking. If your baby still wants to suck after a good feeding, offer a clean finger or pacifier to suck on. This can be very comforting for your baby.

Rhythmic Movement. Use slow, gentle "up and down," rocking, or swaying movements when holding your baby. Pause or stop the movement if your baby becomes upset.

Calm Room & Soothing Sounds. Keep your baby's space calm and quiet with the lights low. Use a quiet voice when talking/singing to your baby. Remember, loud noises and bright lights may upset your baby.

Limit Visitors. Try to have only 1-2 visitors in your room at a time as more may make your baby fussy or not feed or sleep as well. Encourage your visitors to use quiet voices.

Undisturbed Sleep & Clustering Care. Allow your baby to rest undisturbed between feedings. Ask the nurse to assess your baby when he/she is awake and has fed first.

Safe Sleep & Fall Prevention. Make sure you are wide awake when you are holding your baby. If you feel sleepy, ask for someone else to hold your baby. If you are on your own, ask a staff member to hold or place your baby in the bassinet.

Extra Help & Support. Be well rested so you can safely take care of your baby. Ask for another parent, friend, or family member to help with your baby so you can rest. Tell us if you need help finding someone to care for your baby while you rest.

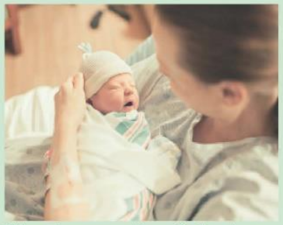

What happens if my baby does need medicine to treat NOWS?

- Some babies may need just one dose of medicine while others may need to be treated for several days. Some babies may need medicine for even longer. It is very important that you stay with your baby as much as possible during this time because you are still the most important treatment for your baby. Please make a plan for how you will be able to be here in case this happens. If you need help making a plan, a social worker may be able to help you.
- If possible, plan to have at least one family member or friend here with you to help care for your baby in your room.
- Bring enough clothes and personal items with you to last at least one week.
- Plan to have someone watch your other children and/or pets while you are away.
- Sometimes it is hard to talk to your family about why your baby might need to stay in the hospital. If this is true for you, ask your OB or Pediatric provider to help. We also have a social worker who can help you with this or any other difficult conversations.

When can I take my baby home?

Your baby's care team will help decide when it is safe for your baby to go home. We will need to watch your baby in the hospital for several days. It is best to have your baby stay in the hospital until most of the symptoms of NOWS are over.

Your baby is usually ready to go home when he/she:

- Is feeding and sleeping well.
- Is easy to console (calm down).
- Has not lost too much weight or is gaining weight.
- Maintains a healthy temperature, heart rate, and breathing.
- Has received all routine newborn care.
- No longer needs medicine (if started) or a plan has been made to continue medicine at home.
- Has follow-up plans in place (primary care physician, visiting nurse, etc.). These visits are an important part of normal newborn care. They are also important to watch your baby's weight, watch for NOWS symptoms, and talk about any concerns you may have.

Original content developed by Dr. Bonny Whalen and the staff at Children's Hospital at Dartmouth-Hitchcock in Lebanon, NH. Modified by the ESC-NOW study team for use by sites participating in the ESC-NOW trial.

NIH HEAL INITIATIVE
NICHD Neonatal Research Network
IDeA States Pediatric Network
ECHO • A program supported by the NIH

ACT NOW ESC Parent Guide V-01 [29-July-2020] cIRB #239729

APPENDIX G: NEWBORN CARE DIARY SAMPLE PAGES

Today's Date June 16, 2020

**Eating**				
Time of Baby's Feeding (start and finish times)	Start 12:40 p.m. Finish 12:15 p.m.	Start ____ Finish ____	Start ____ Finish ____	Start ____ Finish ____
Breast Feeding (total minutes on each side)	Left 15 mins. Right 10 mins.	Left ____ Right ____	Left ____ Right ____	Left ____ Right ____
Bottle Feeding (total number of mililiters)				
Did your baby feed well?	Yes No	Yes No	Yes No	Yes No
If no, please describe.	Last feed was 4 hours ago. Will do skin-to-skin and feed sooner next time.			
**Sleeping**				
Time baby fell asleep	8:00 a.m.			
Time baby woke up	12:15 p.m.			
Did your baby sleep an hour or more?	Yes No	Yes No	Yes No	Yes No
If no, please describe.				
**Consoling**				
Did your baby console in 10 minutes or less?	Yes No	Yes No	Yes No	Yes No
If no, please describe. Add other notes if needed.				
**Diapering**				
Did your baby pee or poop?	Pee Poop	Pee Poop	Pee Poop	Pee Poop
Please describe poop.	Normal Loose Watery	Normal Loose Watery	Normal Loose Watery	Normal Loose Watery
Add any notes.				

**Eating**					
Start ____ Finish____	Start ____ Finish____	Start ____ Finish____	Start ____ Finish____	Start ____ Finish____	Start ____ Finish____
Left ____ Right ____	Left Right	Left Right	Left Right	Left Right	Left Right
Yes No	Yes No	Yes No	Yes No	Yes No	Yes No
**Sleeping**					
Yes No	Yes No	Yes No	Yes No	Yes No	Yes No
**Consoling**					
Yes No	Yes No	Yes No	Yes No	Yes No	Yes No
**Diapering**					
Pee Poop	Pee Poop	Pee Poop	Pee Poop	Pee Poop	Pee Poop
Normal Loose Watery	Normal Loose Watery	Normal Loose Watery	Normal Loose Watery	Normal Loose Watery	Normal Loose Watery

ACT NOW ESC Newborn Care Diary V-01 (29-July-2020) cIRB #239729

Page 1 of 8

APPENDIX H: IMPLEMENTATION GUIDE

Establish Team

- Include key stakeholders: consider inviting nurse managers, nurse educators, nurse champions, outpatient and inpatient provider champions, parent representative, home visiting, recovery or other community supports, etc.
- Schedule regular meetings to plan and implement ESC Care Tool and ESC care approach.
- Review “Potential Challenges and Possible Solutions”.
- Plan for standardized documentation (paper vs EMR flowsheets and Smart Phrases).
- Create timeline for education and implementation.

Education Plan

	Participants	Goals	Methods
Provider	OB<br>Pediatrics<br>Family Medicine<br>Treatment Specialists<br>Inpatient Providers<br>Outpatient Providers	Establish provider buy-in<br>Familiarize providers with ESC Care Tool<br>Share timeline<br>Share educational resources<br>Discuss concerns<br>Answer questions	Email introduction<br>Live education (grand rounds, trainee education, lunch-and-learns, etc.)
Staff	Nurses<br>Pharmacists<br>Transport teams<br>Providers	Establish staff buy-in<br>Teach staff to use ESC Care Tool with >80% interrater reliability<br>Share implementation timeline and educational resources<br>Discuss concerns and answer questions	Live education<br>(offer multiple sessions near change of shift at varying times to capture all staff)
Parents	Parents<br>Other family<br>Friends	Parents understand and expect ESC approach<br>Parents plan for presence in the hospital and respite supports	Update prenatal messaging<br>Educate prenatal providers<br>Share standard parent education pamphlet

Enhance Staff Support

- Create ESC Resource Binder and make available in a common space or via intranet.
- Establish gold-star rater team and schedule at least one gold-star rater on each shift if possible.
- Debrief and review cases regularly with implementation team and bedside staff.
- Maintain Independent Study Tool for ongoing staff training.

APPENDIX I: ENSURING FIDELITY OF IRR SCORES

Please use Video Case 2 or Written Case 5 (links below) to complete the IRRs. These cases will function like Written Cases 5-8 that were completed during the 3-day Site Champion Training. Sites may also complete IRRs using the paper ESC Care Tool or IRR Tool and Written Case 5. If a site chooses to complete IRRs on paper, please upload the completed IRRs here: ****

Video and Written Cases

1. Select 10 nurses and 3 gold-star raters to assess and provide them one of the case links below. Nurses and gold-star raters can complete one of the two cases, either Video Case 2 or Written Case 5.

    a. Video Case 2: ****

    b. Written Case 5: ****

2. The link will open to this screen. Complete the questions. If you need to save and return to the survey later, scroll to the bottom of the screen and click “Save & Return Later” (see screen shot on next page).

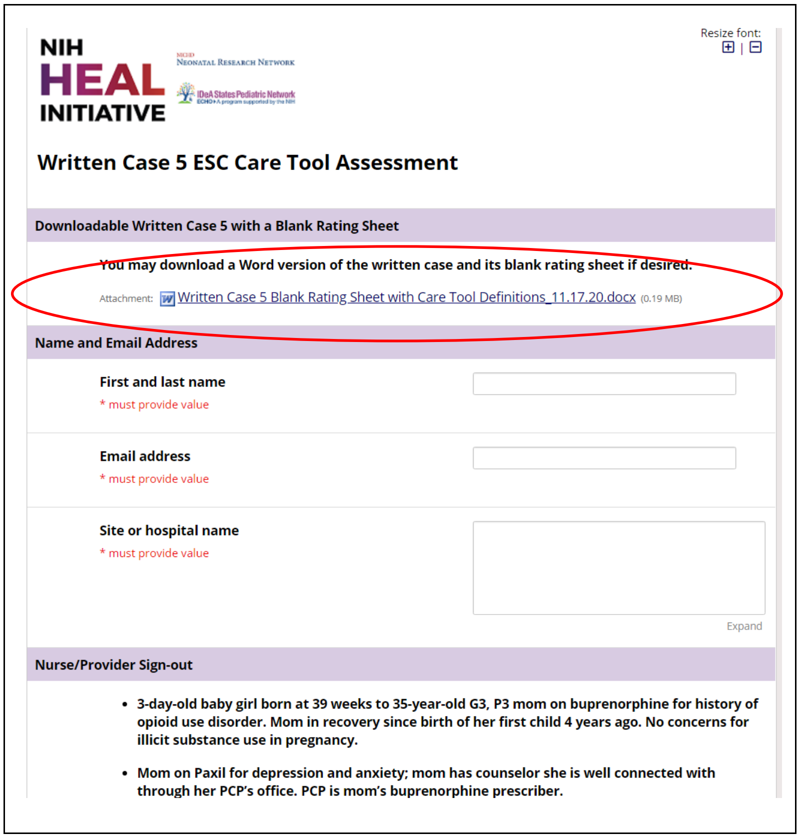

If desired, you can download a Word version of the case with a blank ESC Care Tool.

3. Once you complete the questions and are ready to submit your answers, click “Yes” by “Are you ready to score your full ESC Care Tool.”

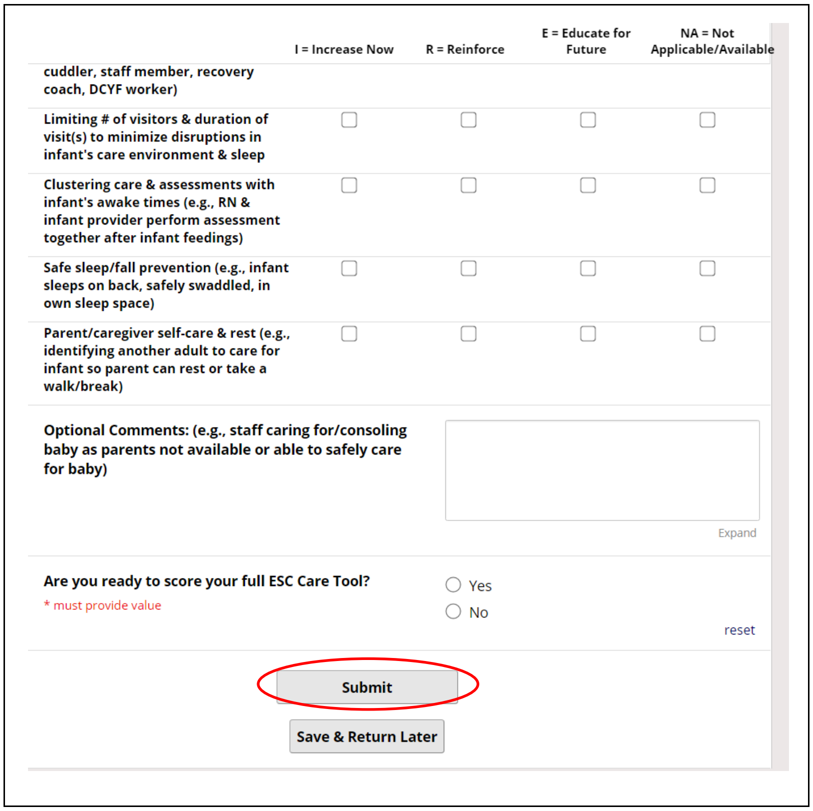

4. When you click “Submit”, you will be taken to this screen where you will find your IRR score for the case.
5. For written cases, you may download the case rating-key and algorithm.
6. For all cases, download your survey responses and **record the IRR score on the response sheet**.
7. Submit the response sheet with the recorded IRR score to the research coordinator or the person responsible for collecting completed IRRs.
8. Keep responses and scores at site for monitoring/auditing visits.

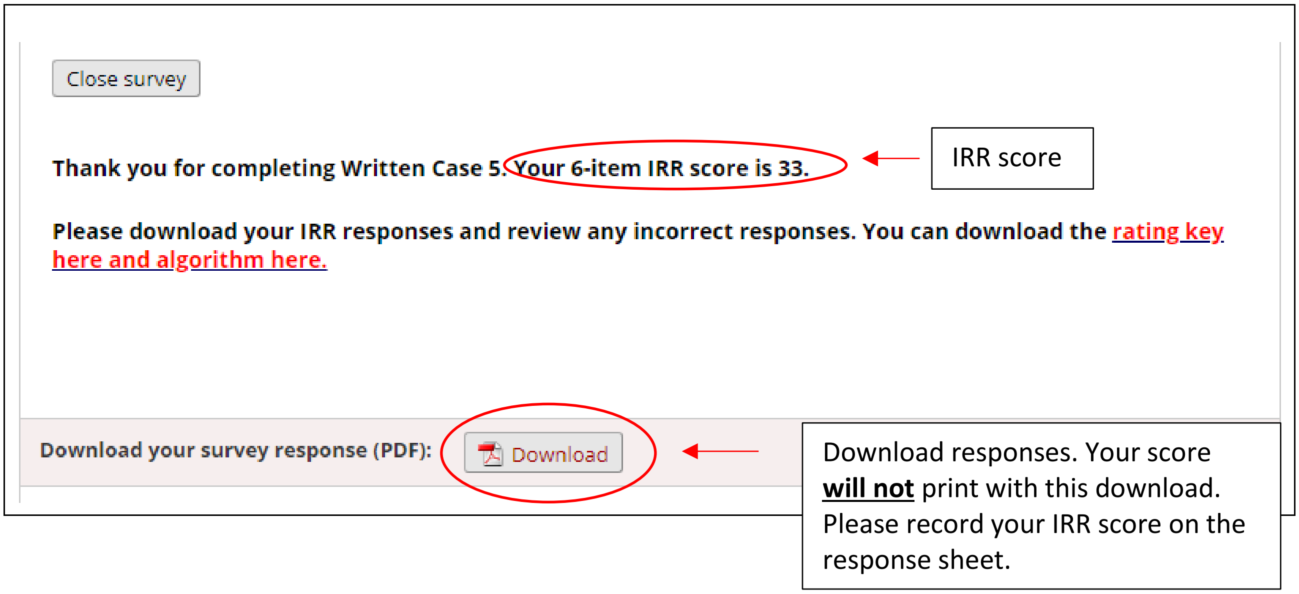

Completing IRR on Paper

If completing IRRs on paper, please use Written Case 5 and complete the following steps.

1. Score the case using the Written Case 5 Rating Key
2. Document the score on the paper case form
3. Confirm the following is on the form for the person completing the case
    a. first and last name
    b. Role – gold-star rater or nurse
    c. Date case completed
    d. Site name
1. Upload paper IRRs here: ****

APPENDIX J: ESC IMPLEMENTATION PROCESS EVALUATION FORM

IMPLEMENTATION PROCESS EVALUATION FORM

Team Member being evaluated: ______________________

Gold Star Rater: ___________________________

Date of Evaluation: __ __/__ __ __/__ __ __ __
DD MON YYYYY

Instructions: The Gold Star Rater will evaluate the team member listed above for ESC Care Tool implementation.
Note: This evaluation should be performed during an ESC assessment that the parent/caregiver is present for.

EVALUATION CRITERIA	Gold Star Rater
The nurse reviewed ESC behaviors with the parent/caregiver(s) since the last assessment using the Newborn Care Diary? or via conversation?	❒Yes ❒ No ❒N/A*
The nurse reviewed signs of withdrawal with the parent/caregiver(s) since the last assessment using the Newborn Care Diary? or via conversation?	❒Yes ❒ No ❒N/A*
If during this assessment the infant had 'Yes' for any ESC item or '3' for 'Consoling Support Needed' a 'Formal Parent/Caregiver Huddle' was performed.	❒Yes ❒No
If the infant had 'Yes' for any ESC item or '3' for 'Consoling Support Needed' on the previous assessment and the infant continued to have a 'Yes' for any of the ESC item or '3' for 'Consoling Support Needed' (or other significant concerns were present) on the current assessment, a 'Full Care Team Huddle' was performed.	❒Yes ❒No
NPIs were maximized to fullest extent possible in infant's clinical setting	❒Yes ❒No ❒N/A*
Rooming-In	❒Yes ❒No ❒N/A*
Parent/caregiver presence	❒Yes ❒No ❒N/A*
Skin-to-skin contact	❒Yes ❒No ❒N/A*
Holding by parent/caregiver/cuddler	❒Yes ❒No ❒N/A*
Safe & effective swaddling	❒Yes ❒No ❒N/A*
Optimal feeding	❒Yes ❒No ❒N/A*
Non-nutritive sucking	❒Yes ❒No ❒N/A*
Quiet, low light environment	❒Yes ❒No ❒N/A*
Rhythmic movement	❒Yes ❒No ❒N/A*
Additional help/support in the room	❒Yes ❒No ❒N/A*
Limiting # of visitors and duration of visit(s)	❒Yes ❒No ❒N/A*
Clustering care	❒Yes ❒No ❒N/A*
Safe sleep/fall prevention	❒Yes ❒No ❒N/A*
Parent/caregiver self-care & rest	❒Yes ❒No ❒N/A*

*Not Applicable/Attainable.

_____________________________________ __ __/__ __ __/__ __ __ __
Printed Name of Gold Star Rater Date

_____________________________________ __ __/__ __ __/__ __ __ __
Signature of Gold Star Rater Date

NIH HEAL INITIATIVE

NICHD Neonatal Research Network

IDeA States Pediatric Network
ECHO • A program supported by the NIH

APPENDIX K: ESC TRIAL SCHEDULE

The figure below is an overall snapshot of study dates and blocks. See the next page for a table of study dates by block.

	Period 1**	Period 2	Period 3	Period 4	Period 5	Period 6	Period 7	Period 8
Block 1*	Usual Care 9/8/20-10/19/20 6 weeks	Transition 10/20/20-1/11/21 12 weeks	ESC 1/12/21-2/22/21 6 Weeks	ESC 2/23/21-4/19/21 8 weeks	ESC 4/20/21-6/14/21 8 Weeks	ESC 6/15/21-8/9/21 8 Weeks	ESC 8/10/21-10/4/21 8 Weeks	ESC 10/5/21-11/[illegible] 8 Weeks
Block 2	Usual Care 9/8/20-11/2/20 8 Weeks	Usual Care 11/3/20-12/14/20 6 Weeks	Transition 12/15/20-3/8/21 12 Weeks	ESC 3/9/21-4/19/21 6 Weeks	ESC 4/20/21-6/14/21 8 Weeks	ESC 6/15/21-8/9/21 8 Weeks	ESC 8/10/21-10/4/21 8 Weeks	ESC 10/5/21-11/[illegible] 8 Weeks
Block 3	Usual Care 9/8/20-11/2/20 8 Weeks	Usual Care 11/3/20-12/28/20 8 Weeks	Usual Care 12/29/20-2/8/21 6 Weeks	Transition 2/9/21-5/3/21 12 Weeks	ESC 5/4/21-6/14/21 6 Weeks	ESC 6/15/21-8/9/21 8 Weeks	ESC 8/10/21-10/4/21 8 Weeks	ESC 10/5/21-11/[illegible] 8 Weeks
Block 4	Usual Care 9/8/20-11/2/20 8 Weeks	Usual Care 11/3/20-12/28/20 8 Weeks	Usual Care 12/29/20-2/22/21 8 Weeks	Usual Care 2/23/21-4/5/21 6 Weeks	Transition 4/6/21-6/28/21 12 Weeks	ESC 6/29/21-8/9/21 6 Weeks	ESC 8/10/21-10/4/21 8 Weeks	ESC 10/5/21-11/[illegible] 8 Weeks
Block 5	Usual Care 9/8/20-11/2/20 8 Weeks	Usual Care 11/3/20-12/28/20 8 Weeks	Usual Care 12/29/20-2/22/21 8 Weeks	Usual Care 2/23/21-4/19/21 8 Weeks	Usual Care 4/20/21-5/31/21 6 Weeks	Transition 6/1/21-8/23/21 12 Weeks	ESC 8/24/21-10/4/21 6 Weeks	ESC 10/5/21-11/[illegible] 8 Weeks
Block 6	Usual Care 9/8/20-11/2/20 8 Weeks	Usual Care 11/3/20-12/28/20 8 Weeks	Usual Care 12/29/20-2/22/21 8 Weeks	Usual Care 2/23/21-4/19/21 8 Weeks	Usual Care 4/20/21-6/14/21 8 Weeks	Usual Care 6/15/21-7/26/21 6 Weeks	Transition 7/27/21-10/18/21 12 Weeks	[illegible] 10/19/2[illegible] 6 [illegible]
Block 7	Usual Care 9/8/20-11/2/20 8 Weeks	Usual Care 11/3/20-12/28/20 8 Weeks	Usual Care 12/29/20-2/22/21 8 Weeks	Usual Care 2/23/21-4/19/21 8 Weeks	Usual Care 4/20/21-6/14/21 8 Weeks	Usual Care 6/15/21-8/9/21 8 Weeks	Usual Care 8/10/21-9/20/21 6 Weeks	Transitio[illegible] 9/21/21-12/[illegible] 12 Wee[illegible]
Block 8	Usual Care 9/8/20-11/2/20 8 Weeks	Usual Care 11/3/20-12/28/20 8 Weeks	Usual Care 12/29/20-2/22/21 8 Weeks	Usual Care 2/23/21-4/19/21 8 Weeks	Usual Care 4/20/21-6/14/21 8 Weeks	Usual Care 6/15/21-8/9/21 8 Weeks	Usual Care 8/10/21-10/4/21 8 Weeks	Usual Care 10/5/21-11/15/21 6 Weeks
